# A general aqueous synthetic strategy towards 1-benzylTHIQs enabled by umpolung hydrazone

**DOI:** 10.1039/d5sc08310a

**Published:** 2026-01-09

**Authors:** Manpreet Kaur, Evan F. W. Chen, Jan Michael Salgado, Ruofei Cheng, Chao-Jun Li

**Affiliations:** a Department of Chemistry, and FRQNT Centre for Green Chemistry and Catalysis, McGill University 801 Sherbrooke Street West Montreal QC H3A 0B8 Canada cj.li@mcgill.ca

## Abstract

Primarily found in plants, 1-benzyltetrahydroisoquinoline (1-benzylTHIQ) alkaloids are a diverse class of N-heterocyclic natural products with biological activity against various infectious diseases and neurodegenerative pathologies. Traditionally, 1-benzylTHIQs are synthesized using commercially inaccessible pre-functionalized materials or hazardous organometallic reagents, making their synthesis challenging. Herein, we developed an environmentally benign synthetic strategy to synthesize 1-benzylTHIQs, which aligns with green chemistry principles. This method utilizes abundant, renewable aldehydes as sustainable alkyl carbanion equivalents, thereby eliminating the use of highly reactive or hazardous organometallic reagents. This reaction is catalyzed by ruthenium, using water as a greener solvent, eliminating the need for organic solvents, thereby reducing its environmental impact. Moreover, only N_2_ and H_2_O are produced as by-products, which minimizes waste generation. A diverse array of substituted 1-benzylTHIQs was synthesized, showing good functional group tolerance (without the need to protect the functional groups) and resulting in moderate to excellent yields. The sustainability of our method was further demonstrated through the synthesis of natural 1-benzylTHIQ based alkaloids and late-stage functionalization of pharmacologically relevant molecules.

## Abstract 2

Ruthenium-catalyzed aqueous umpolung addition of hydrazones provides efficient access to 1-benzylTHIQ scaffolds relevant to natural alkaloid synthesis and drug modification.

## Graphical abstract

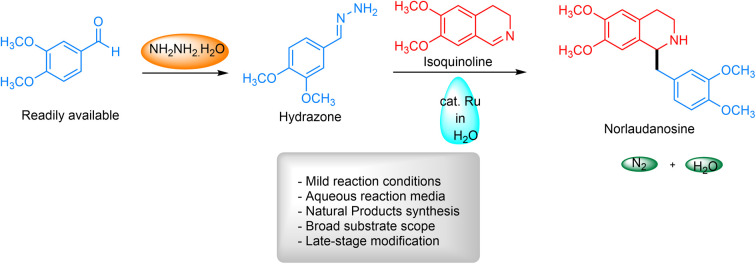

## Introduction

**Fig. 1:**
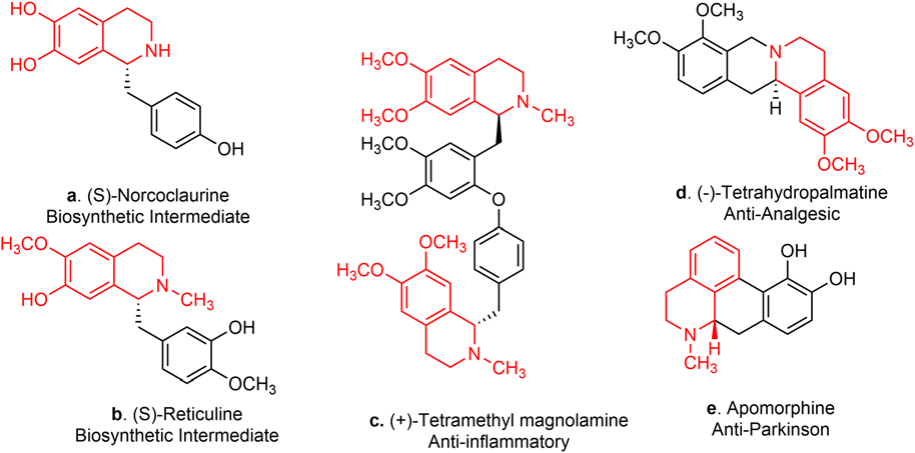
THIQ containing alkaloids.

Tetrahydroisoquinolines (THIQ) are defined as the saturated derivatives of isoquinoline, which are structurally distinguished by a fused piperidine ring. Their derivatives have been the subject of extensive research for decades due to their wide therapeutic potential and pharmacological applications.^1–4^ Notably, C1-functionalization of THIQs serves as a highly effective and versatile technique for the synthesis of polycyclic and multi-substituted derivatives, such as 1-benzylTHIQs.^5^ These important motifs are commonly found in medicinally relevant bioactive compounds and naturally derived alkaloids.^3,6,7^ Well-known examples include tetrahydropalmatine (Fig. 1d) and morphine, which serve as analgesics, and berberine, valued for its antiseptic activity.^8,9^ Other 1-benzylTHIQs, such as norcoclaurine and reticuline, serve as significant precursors in the biosynthesis of morphine and codeine (Fig. 1a and b).^7^

**Fig. 2:**
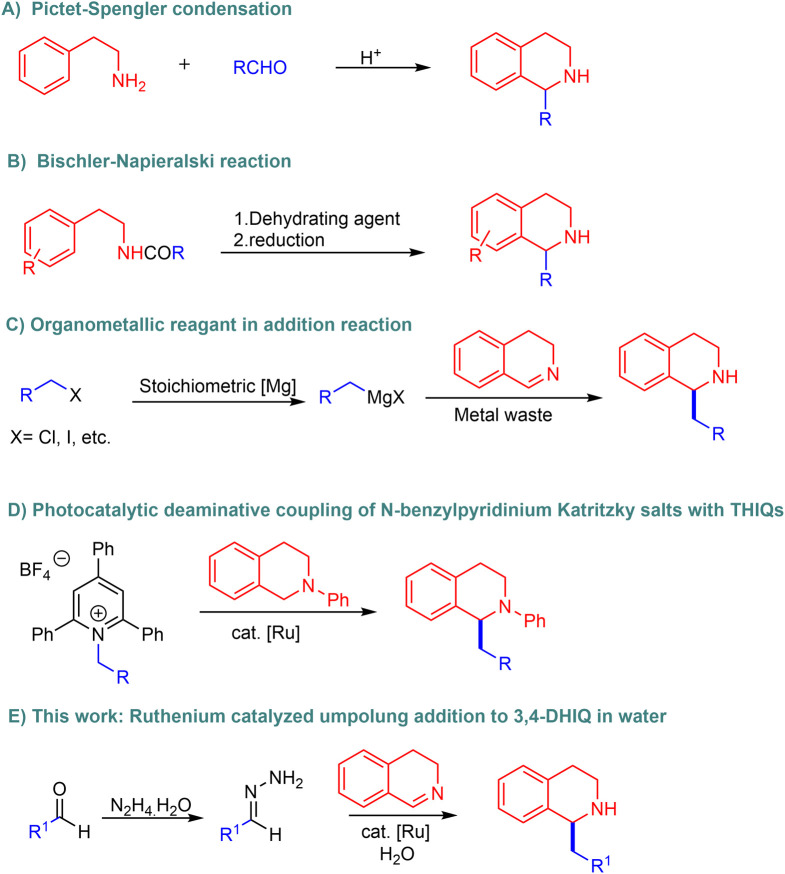
Diverse synthetic strategies for the preparation of 1-benzylTHIQs.

Owing to the pharmaceutical relevance of C1-functionalized compounds and their utility as precursors in alkaloid biosynthesis, over the past decades, synthetic chemists have shown considerable interest in developing complex THIQs by functionalizing simple isoquinolines. Traditional approaches, such as the Pictet–Spengler condensation^10^ (Fig. 2A) and the Bischler–Napieralski reaction (Fig. 2B), are commonly used to synthesize 1-benzylTHIQs.^11,12^ However, these methods have notable drawbacks, including low yields, the need for higher temperatures and extended reaction times, and the use of hazardous reagents such as strong acids.

Alternatively, other pathways to access 1-benzylTHIQs include the Grignard addition of benzyl organometallic reagents to 3,4-dihydroisoquinolines (3,4-DHIQs) (Fig. 2C).^13^ However, most organometallic reagents are synthesized using stoichiometric amounts of the corresponding metals and organic halides. Their use also generates copious amounts of metal halide waste while exhibiting high moisture sensitivity and requires special handling under inert conditions. Moreover, as reported in previous literature, catalytic hydrogenation of isoquinolines has also been explored for the synthesis of THIQs.^14–16^ In recent years, photocatalytic strategies have emerged as promising alternatives. For example, visible-light driven functionalization of *N*-aryl tetrahydroisoquinolines has been reported, providing access to structurally diverse derivatives.^17–19^ Additionally, a Ru-photocatalyzed coupling of *N*-aryl THIQs with Katritzky salts has recently been developed to produce 1-benzylTHIQs (Fig. 2D).^20^ Despite this advancement, the method suffers from several limitations, including poor reaction efficiency, a narrow substrate scope, and limited functional group tolerance. Furthermore, commercially inaccessible pyridinium salts hinder the accessibility of this methodology. Given the significant limitations in current methods, a simple and sustainable methodology for the synthesis of 1-benzylTHIQs is highly desired.

Recently, our research group pioneered methodologies for using hydrazones as organometallic equivalents (HOME) in nucleophilic addition and cross-coupling reactions.^21,22^ In this approach, carbonyl-derived hydrazones act as alkyl carbanion equivalents, coupling with diverse electrophilic partners in the presence of a catalytic amount of transition metals. This strategy involves preparing hydrazones from naturally abundant alcohols and carbonyl compounds derived from renewable feedstocks, generating water and nitrogen gas as innocuous by-products under mild reaction conditions. Previously, our group reported the Ru and Fe-catalyzed umpolung addition of hydrazones to imines to synthesize secondary amines.^23,24^ In addition, ruthenium has been shown to tolerate aqueous environments.^25^ Recent efforts have focused on developing green solvents to replace petroleum-derived alternatives, with emphasis on low toxicity, abundance, safety, cost-effectiveness, and ease of separation. Water, an inexpensive and abundant solvent, aligns with global demands for sustainable and green chemistry.^26–29^ However, the application of this system in THIQ synthesis remains unexplored. Herein, we report a Ru-catalyzed umpolung addition of aryl aldehyde hydrazones onto 3,4-DHIQs in water (Fig. 2E).

## Results and discussion

At the beginning of our investigation, benzaldehyde hydrazone 1a (2 equiv.) was chosen as the model nucleophile reagent, reacting with 3,4-DHIQ 2a (0.2 mmol) as the model electrophile under an atmosphere of N_2_ in THF as solvent. Based on previous literature,^23,24,30^ we initially screened Ru, Mn, and Fe catalysts. We observed that [Ru(*p*-cymene)Cl_2_]_2_ in combination with bis(dimethylphosphino)ethane (dmpe) afforded 3aa in 20% yield (Table 1, entry 1), while no product was observed for the other metals. Then, we explored a variety of monodentate and bidentate phosphine ligands. Monodentate phosphines such as PMe_3_ gave 3aa in 23% yield (entry 2), whereas no product 3aa was observed when 1,2-bis(diphenylphosphino)ethane (dppe) was employed (entry 3). Moreover, amine-based bidentate ligands such as *N*,*N*,*N*′,*N*′-tetramethyl ethylenediamine (TMEDA) also yielded 3aa in 16% yield (entry 4). These results indicate that the reaction was favored by electron-rich and non-bulky phosphine and nitrogen ligands. When the reaction was carried out in other organic solvents such as dioxane and toluene, no improvement in the yield of 3aa was observed (entries 5 and 6). To our surprise, switching the solvent from organic to H_2_O improved the yield of 3aa to 70% (entry 7). This improvement in yield could be due to the hydrophobic effect in water. This effect increases the reaction rate, even when the reactants have poor or no solubility in the solvent.^31–34^ To further improve the yield, catalytic amounts of different phase transfer catalysts were added. Tetrabutylammonium iodide (TBAI) and TPGS-750M were added in catalytic amounts to give 3aa in 86% and 71% yield, respectively (entries 8 and 9). Different bases were also screened for this reaction, with K_3_PO_4_ being the most efficient (see SI and Table 1). Control experiments showed that both catalyst and ligand are required for the reaction (entries 10 and 11). Without surfactant, the reaction gave a 70% yield of product 3aa (entry 7). When carried out under air, the reaction yielded 73% of 3aa (entry 12).

**Table 1:** Optimization of the addition of benzaldehyde hydrazone with 3,4-DHIQ in water

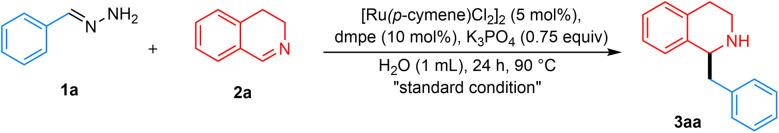		
Entry	Deviation from standard conditions^a^	3aa (yield%)
1	THF as solvent	20
2	PMe_3_ as ligand and THF as solvent	23
3	dppe as ligand and THF as solvent	0
4	TMEDA as ligand and THF as solvent	16
5	Dioxane as solvent	20
6	Toluene as solvent	18
7	None	70
**8**	**TBAI as surfactant**	**86**
9	TPGS-750M as surfactant	71
10	Without [Ru(*p*-cymene)Cl_2_]_2_	3
11	Without dmpe	0
12	Under air	73
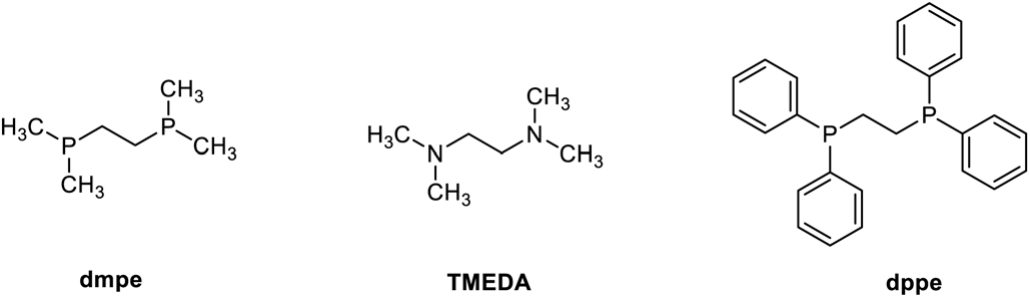		

a Standard conditions: benzaldehyde hydrazone 1a (0.4 mmol, 2 equiv.), 3,4-DHIQ 2 (0.2 mmol), [Ru(*p*-cymene)Cl_2_]_2_ (5 mol%), ligand (10 mol% for bidentate, 20 mol% for monodentate), surfactant (20 mol%), and K_3_PO_4_ (0.15 mmol, 0.75 equiv.) in 1 mL H_2_O for 24 h at 90 °C under Ar. Yields were determined by ^1^H NMR with 1,3,5-trimethoxybenzene as an internal standard.

**Fig. 3:**
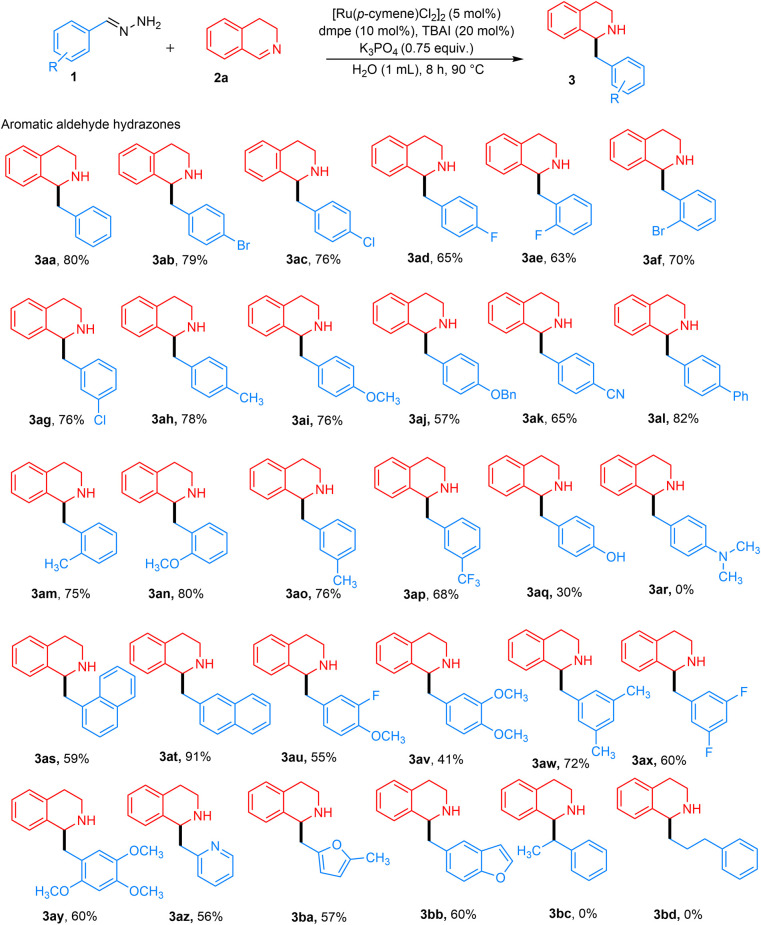
Aryl aldehyde hydrazone substrate scope. Reaction conditions: hydrazone, 1 (0.4 mmol, 2 equiv.), 3,4-DHIQ, 2a (0.2 mmol), [Ru(*p*-cymene)Cl_2_]_2_ (5 mol%), dmpe (10 mol%), TBAI (20 mol%) and K_3_PO_4_ (0.15 mmol, 0.75 equiv.) in 1 mL H_2_O for 8 h at 90 °C under Ar. Isolated yields are reported.

With our optimized conditions from Table 1, entry 8, we next investigated the substrate scope. Various aryl-substituted hydrazones were explored (Fig. 3). Although complete conversion was achieved with benzaldehyde hydrazone 1 and 3,4-DHIQ 2a within 4 h, other substituted hydrazones led to incomplete conversion of 2a. Therefore, the reaction time was extended to 8 h to achieve complete conversion. In general, aryl hydrazones with various substitution patterns afforded moderate to excellent yields.

Hydrazones containing different *para*-substituted halogens, such as chloro, bromo, and fluoro-groups, gave the desired products 3ab–3ad with 65–79% yields. Similarly, *meta*- and *ortho*-halogenated substrates yielded products 3ae–3ag with 63–76% yields. *para*-Substituted hydrazones with electron-donating groups, such as methyl, methoxy, and benzyloxy, produced desired products 3ah–3aj in moderate to high yields of 57–78%. Hydrazones containing *para*-substituted electron-withdrawing groups, such as cyano and phenyl, yielded the desired products 3ak and 3al in high yields ranging from 65–82%. These results indicate that electron-withdrawing groups contribute to improved reaction efficiency, likely due to carbanion stabilization, while both electron-donating and withdrawing groups enable successful product formation. *ortho*-Substituted electron-donating groups such as methoxy and methyl performed well, resulting in the desired products 3am and 3an with yields of 75–80%. *meta*-Substituted benzaldehyde hydrazones were also investigated and gave desired products 3ao–3ap in moderately high yields of 68–76%. In the case of a strongly electron-donating group such as a *para*-substituted hydroxy group, product 3aq was obtained in a 30% NMR yield. In contrast, no product 3ar was detected for the NMe_2_ group, due to the unfavorable formation of the carbanion. 1- and 2-Naphthaldehyde hydrazones worked well, giving the desired products 3as and 3at in yields of 59% and 91%, respectively. The lower yield for 1-naphthaldehyde hydrazone might be due to steric hindrance. Benzaldehyde hydrazones with multiple substituents also proved to be effective substrates, yielding products 3au–3ay in moderate yields of 41–72%.

Heteroaryl aldehyde hydrazones also worked well, affording products 3az–3bb in moderate yields of 56–60%. Acetophenone hydrazones were also investigated, but no product 3bc was observed. The reason could be due to increased bulkiness around the benzylic position of the generated carbanion, as well as the steric hindrance from 3,4-DHIQ, which resulted in no reaction. Consequently, aliphatic hydrazones like 3-phenylpropanal hydrazone did not yield product 3bd due to the absence of an aromatic ring to stabilize the carbanion intermediate.

**Fig. 4:**
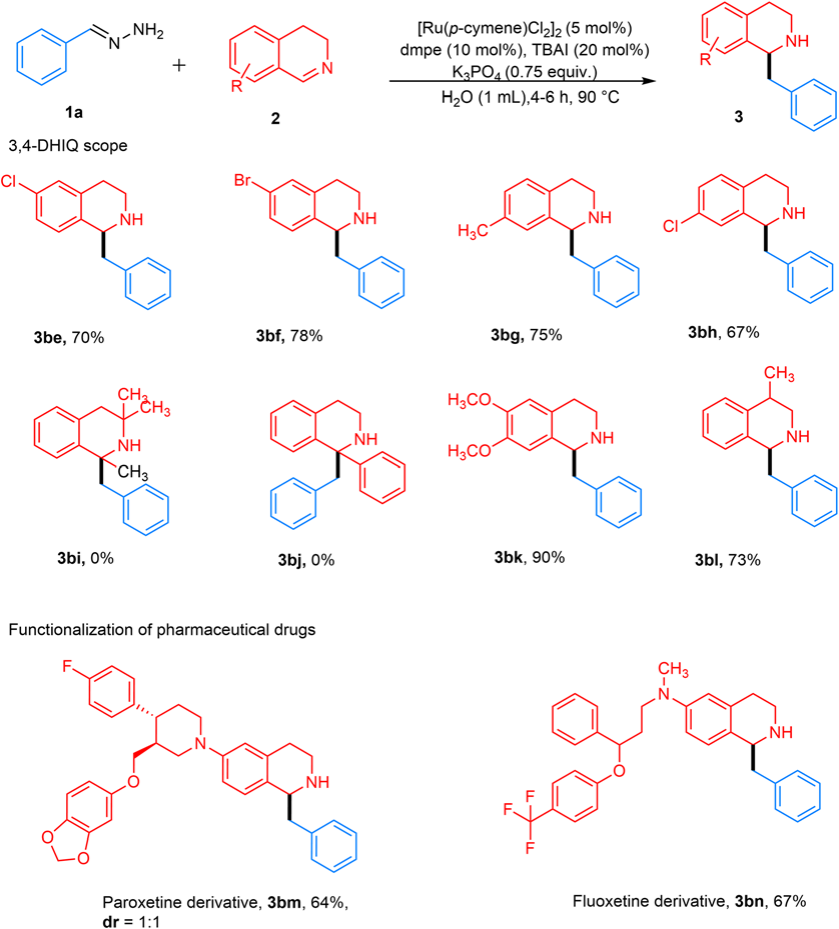
3,4-DHIQ substrate scope. Reaction conditions: hydrazone 1a (0.4 mmol, 2 equiv.), 3,4-DHIQ 2 (0.2 mmol), [Ru(*p*-cymene)Cl_2_]_2_ (5 mol%), dmpe (10 mol%), TBAI (20 mol%), and K_3_PO_4_ (0.15 mmol, 0.75 equiv.) in 1 mL H_2_O for 4–6 h at 90 °C under Ar. Isolated yields are reported.

**Fig. 5:**
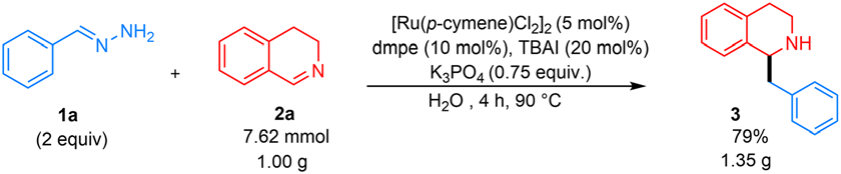
Gram scale synthesis.

Subsequently, various substituted 3,4-DHIQs were investigated (Fig. 4). 6-Substituted chloro and bromo 3,4-DHIQs gave desired products 3be–3bf in high yields of 70–78%. Likewise, 7-substituted chloro and methyl 3,4-DHIQs afforded the desired products 3bg and 3bh in high yields of 67–75%. We also tested our conditions with 1-substituted 3,4-DHIQs, which did not give product 3bi and 3bj. This substitution pattern introduced significant steric hindrance around the 1-position, possibly preventing nucleophilic addition. 6,7-Disubstituted dimethoxy 3,4-DHIQ gave 3bk with an excellent yield of 90%. The reason for this high yield could be that the chelation of methoxy groups with either the ruthenium catalyst or the K cation increases the electrophile's reactivity, making it more susceptible to a carbanion attack. 4-Substituted methyl 3,4-DHIQ also gave a good yield of around 73%. Late-stage functionalization of drugs was then investigated. The reaction with paroxetine and fluoxetine derivatives proceeded well, affording 3bm and 3bn in 64% and 67% yields, respectively. Finally, this method was assessed on a larger scale, and gratifyingly, a 79% yield of 3 was obtained from 1.00 g (7.62 mmol) of 3,4-DHIQ (Fig. 5).

**Fig. 6:**
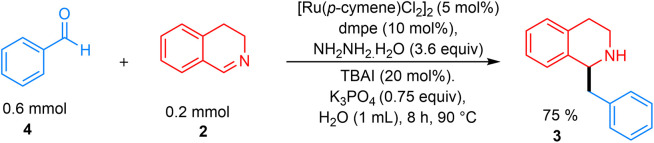
Direct addition of benzaldehyde to 3,4-DHIQ with hydrazine.

**Fig. 7:**
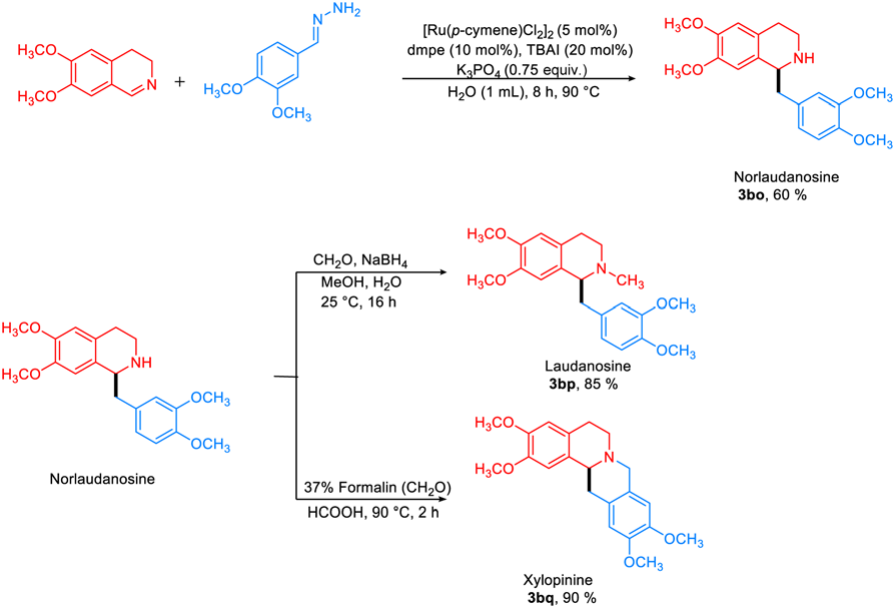
Synthesis of THIQ-containing natural products.

The hydrazine-mediated addition of benzaldehyde to 3,4-DHIQ gave the desired product in 75% yield (Fig. 6). To demonstrate the synthetic applicability of this approach, norlaudanosine (3bo) was synthesized in 60% yield, which was subsequently converted to laudanosine 3bp (85%) and xylopinine 3bq (90%), respectively (Fig. 7).

**Fig. 8:**
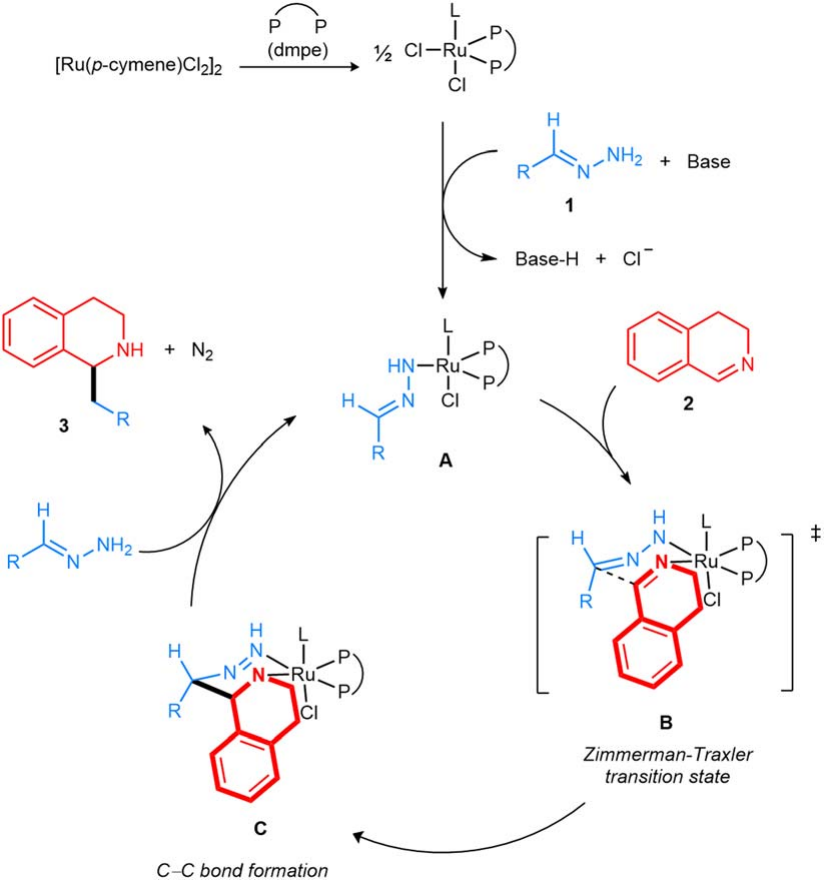
Proposed mechanism for the Ru-catalyzed addition of hydrazones to 3,4-DHIQs.

Based on the previous literature^23–25^ we have proposed the following mechanism (Fig. 8). The Ru complex was generated from [Ru(*p*-cymene)Cl_2_]_2_ with the dmpe ligand. Then, the coordination of deprotonated hydrazone 1a to the dmpe-Ru catalyst formed complex A. The resulting intermediate would combine with 3,4-DHIQ to form a Zimmerman–Traxler chair-like transition state B. Next, intramolecular nucleophilic attack of the carbanion generated from the hydrazone forms the C–C bond, leading to complex C. Finally, by the release of N_2_ under the assistance of the base and coordination of another deprotonated hydrazone, the addition product 3aa was generated, and complex A was reformed.

## Conclusions

In summary, we have developed a method for the umpolung addition of hydrazones to 3,4-DHIQs mediated by a Ru(dmpe) catalytic system. This method facilitates the synthesis of 1-benzylTHIQs through the functionalization of simple 3,4-DHIQs using water as the solvent. The synthetic potential of this method is demonstrated by its broad substrate scope, excellent functional group tolerance, and its applicability to the synthesis of natural alkaloids and the late-stage functionalization of drugs. Earlier methods use DMA, acetonitrile, or THF as solvents, whereas this approach employs a greener solvent. Our method shows a significant improvement over previously reported protocols by adhering to several green chemistry principles, such as waste prevention, the use of renewable feedstocks, safer solvents, and catalysis. Consequently, the method provides a convenient and sustainable route for the functionalization of 3,4-DHIQ.

## Author contributions

MK optimized the reaction, synthesized and purified the substrate scope, and prepared the manuscript. EFWC contributed to the purification of the compounds, general guidance, and manuscript editing. RC provided general guidance and manuscript editing. JMS assisted in the substrate scope and helped in manuscript editing. CJL conceptualized the idea, provided general guidance and edited the manuscript.

## Conflicts of interest

The authors declare no conflict of interest.

## Supplementary Material

SC-017-D5SC08310A-s001

## Data Availability

Supplementary information (SI): all experimental procedures, optimization data and characterisation data of all synthesized compounds. See DOI: https://doi.org/10.1039/d5sc08310a.
