# Supplementary material for: A general aqueous synthetic strategy towards 1-benzylTHIQs enabled by umpolung hydrazone
Source: Chem Sci. 2026 Jan 9. Online ahead of print. doi: 10.1039/d5sc08310a (PMC12805586; doi:10.1039/d5sc08310a)

Supplementary Information (SI) for Chemical Science.
This journal is © The Royal Society of Chemistry 2026

SUPPORTING INFORMATION

A general aqueous synthetic strategy towards 1-benzylTHIQs enabled by HOME chemistry

Manpreet Kaur, Evan F. W. Chen, Jan Michael Salgado, Ruofei Cheng, and Chao-Jun Li

Department of Chemistry, and FRQNT Centre for Green Chemistry and Catalysis, McGill University, 801 Sherbrooke St. W., Montreal, Quebec H3A 0B8 (Canada)

E-mail: cj.li@mcgill.ca

Contents:

1. General Experimental Information: 2

2. Experimental Procedures 2

2.1 General procedure for preparation of hydrazone 2

2.2 General procedure A: addition of 1a to 2a in water 3

2.3. General procedure for the synthesis of natural products 3

3. General Procedure and Characterization Data for Starting Material 5

4. Additional Reaction Optimization Data 8

5. Characterization Data of Compounds 12

6. References: 23

Spectra Collection: 23

1. General Experimental Information.

All solvents and reagents were purchased from Ambeed, TCI chemicals, Combi-blocks, and Oakwood and were used without further purification unless otherwise specified. Reactions in organic solvents were carried out in oven-dried vials sealed with aluminum caps and PTFE-faced silicone septa. Procedures involving organic solvents were conducted under a nitrogen atmosphere, with vials prepared and sealed in a glovebox unless otherwise stated. In contrast, reactions in water were performed in 10 mL oven-dried Schlenk tubes sealed under an argon atmosphere. Air-sensitive ligands, catalysts, and reagents were stored in an MBRAUN UNIlab Pro Glove Box Workstation unless otherwise stated.

All NMR experiments (^1H: 500 MHz; ^{13}C: 100, 125 MHz) were recorded on either a Bruker AV500 or Varian MERCURY plus-500 spectrometer at room temperature. ^1H and ^{13}C NMR chemical shifts (δ) were reported in parts per million (ppm) using the residual solvent signal of $CDCl_3$ as internal standard ($CDCl_3$: δ 7.28 for ^1H; δ 77.00 for ^{13}C). Data are described by δ, multiplicity (s = singlet; d = doublet; dd = doublet of doublets; t = triplet; t = triplet; td = triplet of doublets; q = quartet; quin = quintet; sep = septet; m = multiplet; br = broad), J (Hz), and integration. High-resolution mass spectrometry (HRMS) was performed at McGill University using a maXis Impact™ QTOF mass spectrometer equipped with electrospray ionization (ESI) and atmospheric Pressure Chemical Ionization (APCI). All solvents were purified and dried using established laboratory protocols or were used directly from a solvent purification system without further treatment.

Reagent-grade solvents were used for all work-up and purification procedures. Short-packed column chromatography was performed using Silicycle SiliaFlash silica gel F60 (230–400 mesh) or Biotage Sfär silica HC D 20 µm and a hand-packed neutral alumina column. The Isolera One Prime advanced automatic flash purification system was used for Flash column Chromatography. Thin layer chromatography (TLC) was performed using Silicycle 60Å 250 µm glass-backed plates. Visualization was accomplished with UV light.

2. Experimental Procedures

2.1 General procedure for the preparation of hydrazone

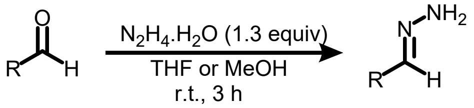

Based on reported literature[1], hydrazones were prepared: to a 25 mL round-bottom flask equipped with a magnetic stir bar, hydrazine monohydrate (3.12 mmol, 1.3 equiv.) and solvent (THF or MeOH, 0.5 mL) were added. For polar aldehydes, methanol (MeOH) was used as a solvent. While stirring, the aldehyde (2.40 mmol, 1.0 equiv) was dissolved in solvent (THF or MeOH, 10 mL) and added dropwise to the solution of hydrazine over 10 minutes. An appropriate amount of

anhydrous Na_2SO_4 was added to remove water. After stirring for another 2-3 h, Na_2SO_4 was filtered off, and the organic layer was extracted with H_2O (15 mL) and CH_2Cl_2 (15 mL). The organic layer was washed with brine (15 mL) and dried over Na_2SO_4. Under reduced pressure, the solvent was removed at room temperature to isolate the hydrazone. For polar aldehydes, no extraction was performed. The solvent was removed under reduced pressure at room temperature, and the hydrazone was isolated directly.

2.2 General procedure for the synthesis of 1-benzyl substituted THIQ

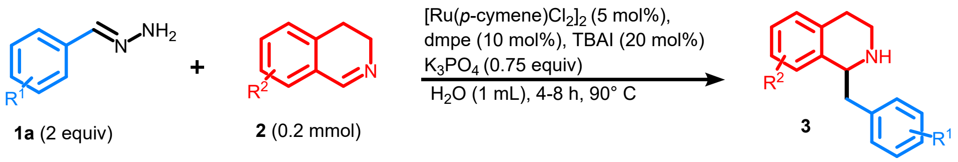

To an oven-dried 10 mL Schlenk tube with a magnetic stir bar, [Ru(*p*-cymene)Cl_2]$_2$ (6.12 mg, 0.01 mmol), K_3PO_4 (31.8 mg, 0.75 equiv, 0.15 mmol), 3,4-DHIQ (26.2 mg, 0.2 mmol), and TBAI (14.77 mg, 0.04 mmol, 0.2 equiv) were added. The mixture was transferred into a glovebox, where dmpe (3 µL, 0.02 mmol) was added, and the tube was removed from the glovebox. Afterwards, hydrazone (0.4 mmol, 2 equiv) was added, followed by 1 mL of degassed water. For 5 min, air was exchanged with an argon atmosphere, the lid was tightened, and the reaction was sealed. Then the reaction mixture was sonicated for 3-4 min and then stirred at 90 °C for 4 h. Upon completion, the reaction mixture was extracted with ethyl acetate (2 x 15 mL) and concentrated under reduced pressure. The residue was purified by flash chromatography using a hand-packed neutral alumina (Al_2O_3) column with a co-solvent system (ethyl acetate and hexane, 1:1 and then 2–4% methanol/DCM gradient).

2.3- Procedure for synthesis of natural products.

Synthesis of Norlaudanosine (3bo)

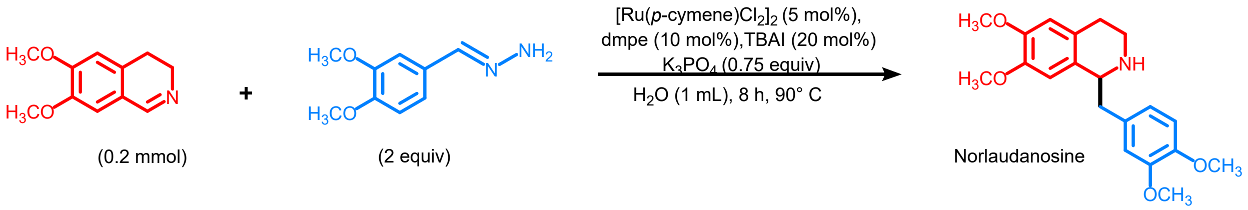

To an oven-dried 10 mL Schlenk tube with a magnetic stir bar, [Ru(*p*-cymene)Cl_2]$_2$ (6.12 mg, 0.01 mmol), TBAI (14.77 mg, 0.04 mmol), K_3PO_4 (31.8 mg, 0.75 equiv), and 6,7-dimethoxy-3,4-DHIQ (38.24 mg, 0.2 mmol) were introduced. The mixture was transferred into a glovebox, where dmpe (3 µL, 0.02 mmol) was added, and the tube was removed from the glovebox. Afterwards, hydrazone (2 equiv) was added, followed by 1 mL of degassed water. For 5 min, air was exchanged with an argon atmosphere, the lid was tightened, and the tube was sealed. The reaction mixture was sonicated for 3-4 min and then stirred at 90 °C for 8 h. Upon completion, the mixture was

extracted with ethyl acetate (2 x 15 mL) and concentrated under vacuum. The residue was purified by Flash chromatography over a hand-packed neutral alumina column (Al_2O_3) using a cosolvent system (ethyl acetate: hexane, 1:1 and 2-4% methanol/DCM gradient), red oil. ^{1}H NMR (500 MHz, $CDCl_3$) δ 6.84 – 6.80 (m, 1H), 6.80 – 6.74 (m, 2H), 6.62 (s, 1H), 6.58 (s, 1H), 4.16 (dd, J = 8.9, 4.7 Hz, 1H), 3.86 (s, 3H), 3.85 (s, 3H), 3.84 (s, 3H), 3.81 (s, 3H), 3.26 – 3.12 (m, 2H), 2.97 – 2.86 (m, 2H), 2.74 (tdd, J = 16.1, 11.3, 5.8 Hz, 2H). ^{13}C NMR (126 MHz, $CDCl_3$) δ 148.9, 147.7, 147.5, 147.0, 131.2, 129.9, 127.2, 121.4, 112.4, 111.8, 111.3, 109.4, 56.7, 55.9, 55.9, 55.8, 55.8, 42.1, 40.8, 29.0.

Synthesis of Laudanosine (3bp)

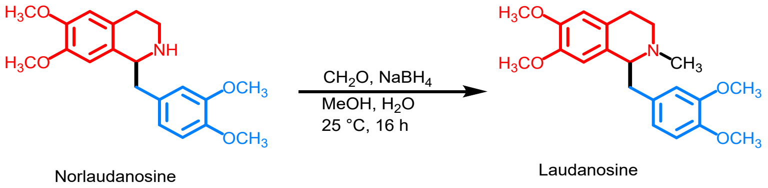

To a solution of Norlaudanosine (70 mg, 0.2 mmol) in MeOH (1.28 mL), aqueous CH_2O (0.42 mL, c = 37 %, 0.0056 mmol) was added. After stirring the reaction for 3 h at 25 °C, $NaBH_4$ (85 mg, 2.24 mmol) was added slowly. The reaction mixture was stirred for an additional 16 h at 25 °C. Saturated aqueous NH_4Cl (5.3 mL) was added, and the mixture was extracted with CH_2Cl_2 (3 × 7 mL). The combined organic layers were dried over Na_2SO_4, and the solvent was concentrated under reduced pressure. The crude product was purified by flash chromatography over neutral Al_2O_3 as a cream-colored solid, giving 85% yield. ^{1}H NMR (500 MHz, $CDCl_3$) δ 6.76 (d, J = 8.1 Hz, 1H), 6.66 – 6.59 (m, 2H), 6.56 (s, 1H), 6.03 (s, 1H), 3.84 (s, 3H), 3.84 (s, 3H), 3.79 (s, 3H), 3.75 – 3.71 (m, 1H), 3.56 (s, 3H), 3.26 – 3.15 (m, 2H), 2.88 – 2.74 (m, 3H), 2.65 – 2.58 (m, 1H), 2.56 (s, 3H). δ^{13}C NMR (126 MHz, $CDCl_3$) δ 148.6, 147.4, 147.4, 146.3, 132.1, 128.6, 125.6, 121.9, 113.0, 111.1, 111.1, 111.0, 64.8, 55.9, 55.8, 55.7, 55.5, 46.7, 42.4, 40.8, 25.2. Analytical data were consistent with literature values.[6]

Synthesis of Xylopinine (3bq)

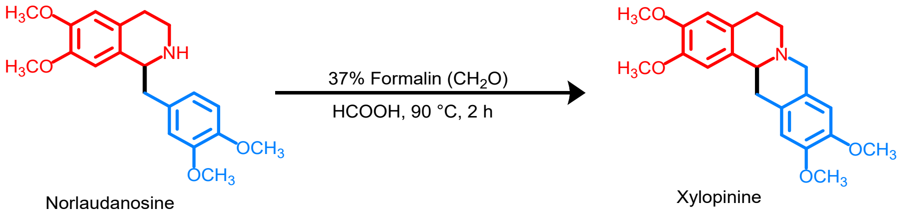

In a 10 mL Schlenk tube, Norlaudanosine (80 mg, 0.23 mmol), 0.33 mL of 88% formic acid, and 0.22 mL of 37% formalin solution were combined and heated at 90 °C for 2 h under a nitrogen atmosphere. After the reaction mixture was cooled to room temperature, it was basified with saturated $NaHCO_3$ and extracted with dichloromethane (3 × 10 mL). The combined organic layers

were dried over Na_2SO_4, filtered, and concentrated under reduced pressure. The crude product was purified by column chromatography on neutral alumina. (Al_2O_3), yielding 70%, yellow solid. ^1H NMR (500 MHz, $CDCl_3$) δ 6.73 (s, 1H), 6.66 (s, 1H), 6.61 (s, 1H), 6.57 (s, 1H), 3.94 (d, *J* = 14.5 Hz, 1H), 3.88 (s, 3H), 3.86 (s, 3H), 3.85 (s, 3H), 3.84 (s, 3H), 3.67 (d, *J* = 14.5 Hz, 1H), 3.58 (dd, *J* = 11.3, 3.9 Hz, 1H), 3.24 (dd, *J* = 15.7, 3.9 Hz, 1H), 3.19 – 3.09 (m, 2H), 2.83 (dd, *J* = 15.9, 12.0 Hz, 1H), 2.69 – 2.57 (m, 2H). ^{13}C NMR (126 MHz, $CDCl_3$) δ 147.6, 147.4, 147.4, 147.4, 129.7, 126.7, 126.3, 126.2, 111.3, 111.3, 109.0, 108.5, 59.6, 58.2, 56.0, 55.9, 55.9, 55.8, 51.3, 36.4, 29.0. Analytical data were consistent with literature values.[5,6]

3. General Procedure and Characterization Data for Starting Compounds 2a-2f

General procedure: *N*-bromosuccinimide (NBS) (4.0 mmol, 4.0 equiv) was added to a solution of 1,2,3,4-tetrahydroisoquinoline (1mmol, 1.0 equiv) in anhydrous CH_2Cl_2 at room temperature under a nitrogen atmosphere. The reaction mixture was stirred at room temperature for 1 h. A 30% v/v NaOH aqueous solution was then added, and the mixture was stirred for an additional hour at room temperature. Then, the aqueous phase was extracted with CH_2Cl_2 (3 x 15 mL), and the combined organic layers were dried over anhydrous Na_2SO_4 and concentrated under reduced pressure.[1]

6-Chloro-3,4-dihydroisoquinoline (2a)

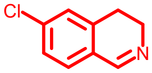

6-Chloro-1,2,3,4-tetrahydroisoquinoline (75%) was synthesized according to general procedure 3 as a yellow oil without further purification. : ^1H NMR (500 MHz, $CDCl_3$): δ 8.29 (1H, t, J=2.1 Hz), 7.25 (1H, m), 7.18 (1H, d, J=8.0 Hz), 7.14 (1H, s), 3.75 (2H, ddd, J=2.3, 7.3, 8.2 Hz), 2.71 (2H, dd, J=8.0, 7.6 Hz).). ^{13}C NMR (126 MHz, $CDCl_3$): δ 159.1, 138.1, 136.6, 128.3, 127.6, 127.2, 126.8, 46.9, 24.8. Data is consistent with literature.[2,3]

6-Bromo-3,4-dihydroisoquinoline (2b)

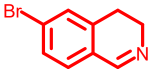

6-Bromo-1,2,3,4-tetrahydroisoquinoline (80%) was synthesized according to general procedure 3 as a yellow oil without further purification. : ^1H NMR (400 MHz, $CDCl_3$) δ 8.29 (s, 1H), 7.43 (dd, J = 8.0, 1.6 Hz, 1H), 7.31 (s, 1H), 7.13 (d, J = 8.0 Hz, 1H), 3.77–3.73 (m, 2H), 2.72 (t, J = 8.0 Hz, 2H); ^{13}C NMR (100 MHz, $CDCl_3$) δ 159.3, 138.3, 130.5, 130.2, 128.5, 127.1, 125.1, 46.9, 24.7, Data is consistent with literature.[2]

7-Methyl-3,4-dihydroisoquinoline (2c)

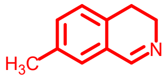

7-Methyl-3,4-dihydroisoquinoline (60%) was synthesized according to General Procedure 3 as a yellow oil and purified using a mixture of ethyl acetate and hexane. ^{1}H-NMR (400 MHz, $CDCl_3$): δ 8.27 (s, 1H), 7.14 (d, J = 7.7 Hz; 1H), 7.05 (s, 1H), 7.01 (d, J = 7.7 Hz; 1H), 3.72 (t, J = 7.8 Hz; 2H), 2.67 (t, J =7.8 Hz; 2H), 2.33 (s, 3H); ^{13}C-NMR (100 MHz, $CDCl_3$): δ 160.3, 136.5, 133.1, 131.5, 128.2, 127.7, 127.1, 47.4, 24.5, 20.8. Data is consistent with literature.[4]

4-methyl-3,4-dihydroisoquinoline (2d)

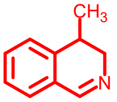

4-methyl-3,4-dihydroisoquinoline (65%) was synthesized according to general procedure 3 as a yellow solid and purified by using ethyl acetate and hexane.^{1}H NMR (500 MHz, $CDCl_3$) δ 8.37 (s, 1H), 7.41 (td, J = 7.3, 1.8 Hz, 1H), 7.34 – 7.27 (m, 2H), 7.27 – 7.23 (m, 1H), 3.84 (ddd, J = 16.1, 6.2, 2.2 Hz, 1H), 3.55 (ddd, J = 16.2, 8.8, 2.3 Hz, 1H), 2.94 – 2.86 (m, 1H), 1.26 (d, J = 7.1 Hz, 3H). ^{13}C NMR (126 MHz, $CDCl_3$) δ 160.3, 141.3, 131.4, 127.6, 127.3, 126.8, 125.5, 54.5, 29.1, 17.5. HRMS (ESI) m/z calculated for $C_{10}H_{12}N([M+H]^+)$: 146.0964, found: 146.0964.

General procedure for the synthesis of 2e and 2f

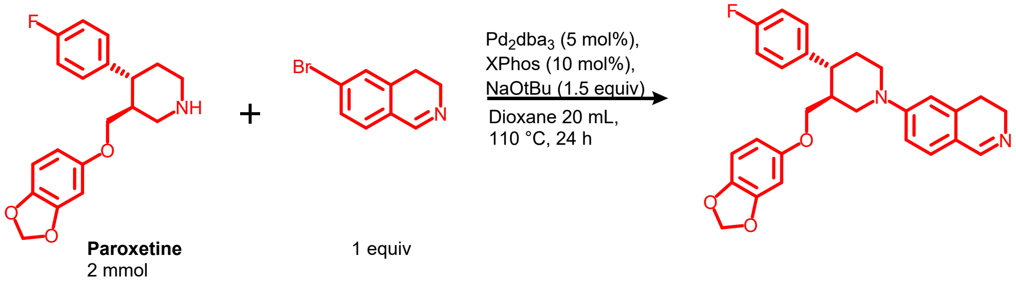

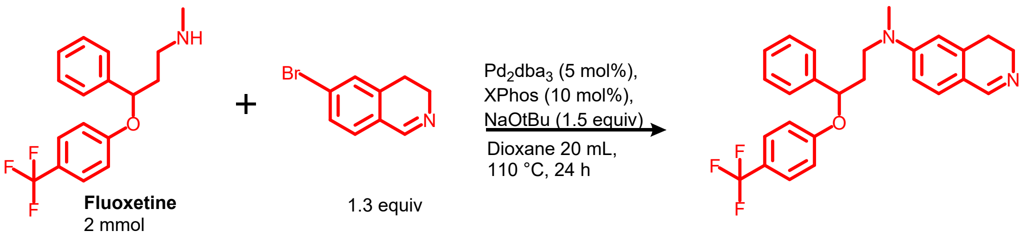

In a 25 mL Schlenk tube equipped with a magnetic stir bar, Pd_2dba_3 (5 mol%), Xphos (10 mol%), NaOtBu (1.5 equiv), Fluoxetine or Paroxetine (2.0 mmol), and 6-bromoisoquinoline (2.0 mmol,

1.0 equiv for Paroxetine or 2.6 mmol, 1.3 equiv for Fluoxetine) were taken in 20 mL dioxane. The reaction mixture was stirred at 110 °C under an argon atmosphere for 24 h. After completion, the product was isolated as a yellow solid by silica gel column chromatography using 10-20 % methanol and DCM.

2-((3S,4R)-3-((benzo[d][1,3]dioxol-5-yloxy)methyl)-4-(4-fluorophenyl)piperidin-1-yl)-3,4-dihydroisoquinoline (2e)

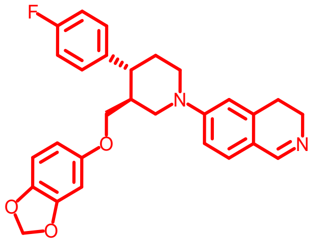

2-((3S,4R)-3-((benzo[d][1,3]dioxol-5-yloxy)methyl)-4-(4-fluorophenyl)piperidin-1-yl)-3,4-dihydroisoquinoline (2e) was prepared according to general procedure. Yield, 60%, yellow solid**.** ^{1}H NMR (500 MHz, $CDCl_3$) δ 8.22 (s, 1H), 7.18 (d, *J* = 8.4 Hz, 1H), 7.16 – 7.09 (m, 2H), 7.00 – 6.92 (m, 2H), 6.81 (dd, *J* = 8.5, 2.5 Hz, 1H), 6.69 (d, *J* = 2.6 Hz, 1H), 6.61 (d, *J* = 8.5 Hz, 1H), 6.37 (d, *J* = 2.5 Hz, 1H), 6.15 (dd, *J* = 8.5, 2.5 Hz, 1H), 5.84 (s, 2H), 4.17 (dd, *J* = 12.9, 2.1 Hz, 1H), 3.96 (dd, *J* = 14.7, 2.1 Hz, 1H), 3.71 (td, *J* = 7.9, 2.0 Hz, 2H), 3.64 (dd, *J* = 9.5, 3.0 Hz, 1H), 3.57 – 3.48 (m, 1H), 2.99 – 2.93 (m, 1H), 2.89 (t, 1H), 2.72 (t, 2H), 2.66 (td, 1H), 2.28 – 2.15 (m, 1H), 1.90 – 1.81 (m, 2H). ^{13}C NMR (126 MHz, $CDCl_3$) δ 161.6 (d, *J* = 244.8 Hz), 159.9, 154.1, 153.1, 148.2, 141.7, 138.9 (d, *J* = 3.2 Hz), 138.3, 129.8, 128.7 (d, *J* = 7.7 Hz), 119.3, 115.5 (d, *J* = 21.0 Hz), 113.5, 113.0, 107.9, 105.6, 101.1, 98.0, 69.1, 67.0, 49.0, 46.5, 44.1, 41.4, 33.6, 26.0. ^{19}F NMR (471 MHz, $CDCl_3$) δ -115.64. HRMS (ESI) m/z calculated for $C_{28}H_{28}FN_2O_3$([M+H]$^+$): 459.2078, found: 459.2080.

N-methyl-N-(3-phenyl-3-(4-(trifluoromethyl)phenoxy)propyl)-3,4-dihydroisoquinolin-6-amine (2f)

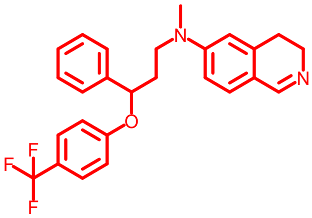

N-methyl-N-(3-phenyl-3-(4-(trifluoromethyl)phenoxy)propyl)-3,4-dihydroisoquinolin-6-amine (2f) was prepared according to the general procedure. Yield, 51%, Yellow solid. ^{1}H NMR (500 MHz, $CDCl_3$) δ 8.19 (s, 1H), 7.44 (d, *J* = 8.7 Hz, 2H), 7.36 – 7.30 (m, 4H), 7.30 – 7.26 (m, 1H), 7.13 (d, *J* = 8.5 Hz, 1H), 6.90 (d, *J* = 8.3 Hz, 2H), 6.52 (dd, *J* = 8.5, 2.6 Hz, 1H), 6.36 (d, *J* = 2.5 Hz, 1H), 5.20 (dd, *J* = 8.3, 4.2 Hz, 1H), 3.75 – 3.61 (m, 3H), 3.60 – 3.51 (m, 1H), 3.00 (s, 3H),

2.67 – 2.57 (m, 1H), 2.57 – 2.42 (m, 1H), 2.27 – 2.13 (m, 2H). ^{13}C NMR (126 MHz, $CDCl_3$) δ 160.1, 160.0, 151.7, 140.3, 138.5, 130.2, 128.9, 128.1, 126.8 (q, J = 3.6 Hz), 125.6, 124.3 (q, J = 272.2 Hz), 123.1 (q, J = 32.5 Hz), 117.2, 115.7, 110.1, 109.6, 77.5, 48.6, 46.0, 38.4, 36.2, 26.0. ^{19}F NMR (471 MHz, $CDCl_3$) δ -61.56. HRMS (ESI) m/z calculated for $C_{26}H_{26}F_3N_2O([M+H]^+)$: 439.1992, found: 439.1992.

4. Optimization Data of Reaction Conditions

Table S1. Optimization of pre-catalyst

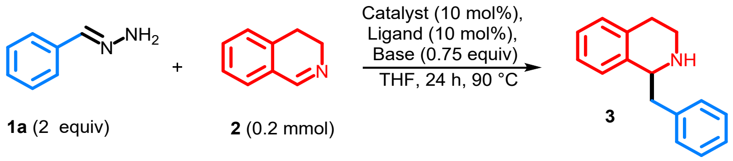

Entry	Catalyst	Ligand	Base	Yield 3 (%)
1	[Ru(p-cymene)Cl_2]$_2$	dmpe	K_3PO_4	20
2	[Ru(PPh_3)$_3$$Cl_2$]	dycpb	K_3PO_4	7
3	[Ru(COD)Cl_2]$_n$	dmpe	K_3PO_4	9
4	Ru(PNP^{ph})(PMe_3)Cl_2		LiOtBu	5
5	Mesityl copper(I)	dppe	LiOtBu	0
6	[Ir(OCH_3)(C_8H_{12})]$_2$	dmpe	K_3PO_4	0
7	$FeCl_2$	dmpe	K_3PO_4	0
8	$Mn_2(CO)_{10}$	PNP^{ph}	K_3PO_4	0

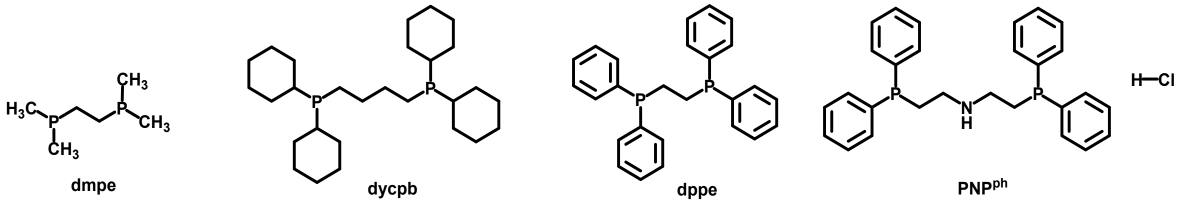

a) Reaction conditions: Hydrazone **1a** (0.4 mmol, 2 equiv), 3,4-DHIQ **2** (0.2 mmol), catalyst (10 mol%), ligand (monodentate, 20 mol%, bidentate, 10 mol%), and base (0.15 mmol, 0.75 equiv) in 1.0 mL THF at 90 °C for 24 h under N_2. ^1H NMR determined yields with 1,3,5-trimethoxybenzene as an internal standard.

Table S2. Optimization of ligand

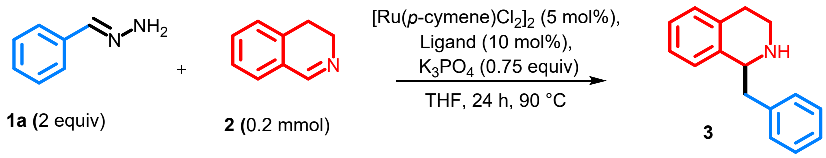

Entry	Ligand	Yield 3 (%)
1	dmpe	20
2	dppe	0

3	PMe_3	23
4	PCy_3	20
5	PNP	0
6	dppf	0
7	$H_2N(CH_2)_2NH_2$	20
8	TMEDA	16

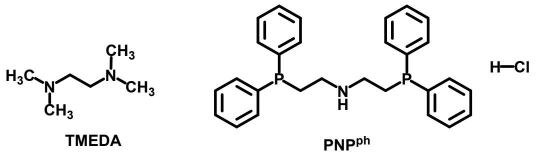

a) Reaction conditions: Hydrazone **1a** (0.4 mmol, 2 equiv), 3,4-DHIQ **2** (0.2 mmol), [Ru(*p*-cymene)Cl_2]$_2$ (10 mol%), ligand (monodentate, 20 mol%, bidentate, 10 mol%), and base (0.15 mmol, 0.75 equiv) in 1.0 mL THF at 90 °C for 24 h under N_2. ^{1}H NMR determined yields with 1,3,5-trimethoxybenzene as an internal standard.

Table S3. Optimization of base loading

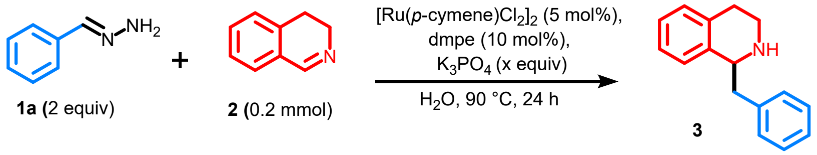

Entry	x equiv	Yield (%)
1	1.00	57
2	0.75	70
3	0.50	61

a) Reaction conditions: Hydrazone **1a** (0.4 mmol, 2 equiv), 3,4-DHIQ **2** (0.2 mmol), [Ru(*p*-cymene)Cl_2]$_2$ (10 mol%), dmpe (10 mol%) and K_3PO_4 (x equiv) in 1.0 mL H_2O at 90 °C for 24 h under Ar. ^{1}H NMR determined yields with 1,3,5-trimethoxybenzene as an internal standard.

Table S4. Optimization of solvent

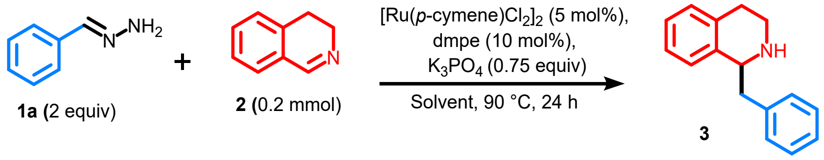

Entry	Solvent	Yield (%)
1	THF	20
2	1,4-dioxane	20
3	Toulene	18

4	H_2O	70

a) Reaction conditions: Hydrazone **1a** (0.4 mmol, 2 equiv), 3,4-DHIQ **2** (0.2 mmol), [Ru(*p*-cymene)Cl_2]$_2$ (10 mol%), dmpe (10 mol%), and K_3PO_4 (0.15 mmol, 0.75 equiv) in 1.0 mL solvent at 90 °C for 24 h under Ar. ^{1}H NMR-determined yields using 1,3,5-trimethoxybenzene as an internal standard.

Table S5. Optimization of surfactant and ligand

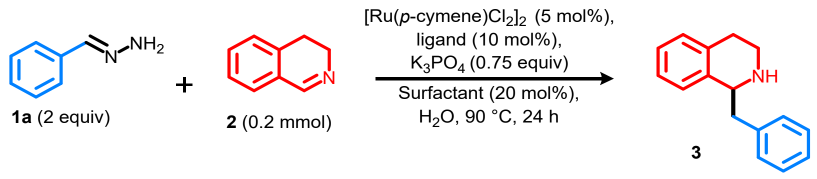

Entry	Surfactant	Ligand	Yield 3 (%)
1	18-crown-6	dmpe	60
2	TPGS-750M	TMEDA	75
3	TPGS-750M	dmpe	71
4	Triton-X-100	dmpe	61
5	Brij C10	dmpe	62
6	TBAI	dmpe	86(80)
7	TBACl	PMe_3	64
8	TBACl	dmpe	67
9	TBACl	dycpb	54
10	TBACl	TMEDA	62
11	TBACl	$H_2N(CH_2)_6NH_2$	70
12	TBAH	dmpe	28
13	TBAB	dmpe	74

a) Reaction conditions: Hydrazone **1a** (0.4 mmol, 2 equiv), 3,4-DHIQ **2** (0.2 mmol), [Ru(*p*-cymene)Cl_2]$_2$ (10 mol%), ligand (10 mol%), K_3PO_4 (0.15 mmol, 0.75 equiv), and surfactant (20 mol%) in 1.0 mL H_2O at 90 °C for 24 h under Ar. ^{1}H NMR determined yields with 1,3,5-trimethoxybenzene as an internal standard.

Table S6. Optimization of catalyst and ligand loading

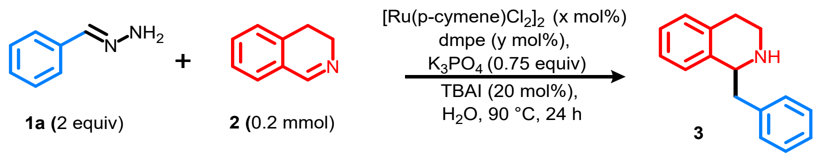

Entry	Catalyst (mol%)	Ligand (mol%)	Yield 3 (%)
1	1.5	3	57
2	4	8	63
3	5	10	86

a) Reaction conditions: Hydrazone **1a** (0.4 mmol, 2 equiv), 3,4-DHIQ **2** (0.2 mmol), [Ru(*p*-cymene)Cl_2]$_2$ (x mol%), dmpe (y mol%), K_3PO_4 (0.15 mmol, 0.75 equiv), and surfactant (20 mol%) in 1.0 mL H_2O at 90 °C for 24 h under Ar. ^{1}H NMR determined yields with 1,3,5-trimethoxybenzene as an internal standard.

Table S7. Optimization of Base

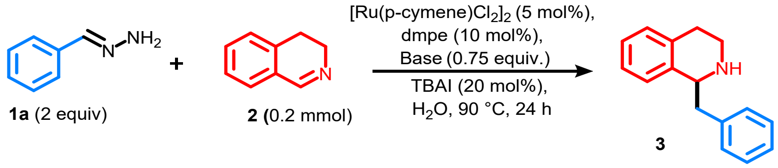

Entry	Base	Yield 3 (%)
1	K_3PO_4	86
2	LiO^{t}Bu	80
3	Cs_2CO_3	n.d
4	K_2CO_3	53
5	DBU	n.d

a) Reaction conditions: Hydrazone **1a** (0.4 mmol, 2 equiv), 3,4-DHIQ **2** (0.2 mmol), [Ru(*p*-cymene)Cl_2]$_2$ (x mol%), dmpe (y mol%), K_3PO_4 (0.15 mmol, 0.75 equiv), and surfactant (20 mol%) in 1.0 mL H_2O at 90 °C for 24 h under Ar. ^{1}H NMR determined yields with 1,3,5-trimethoxybenzene as an internal standard.

Table S8. Optimization of reaction time

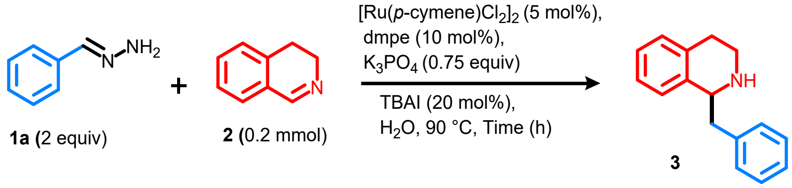

Entry	Time (h)	Yield 3 (%)
1	48 h	81
2	12 h	80
3	6 h	83
4	4 h	86

a) Reaction conditions: Hydrazone **1a** (0.4 mmol, 2 equiv), 3,4-DHIQ **2** (0.2 mmol), [Ru(*p*-cymene)Cl_2]$_2$ (x mol%), dmpe (y mol%), K_3PO_4 (0.15 mmol, 0.75 equiv), and TBAI (20 mol%) in 1.0 mL H_2O at 90 °C for time (h) under Ar. ^{1}H NMR determined yields with 1,3,5-trimethoxybenzene as an internal standard.

Table S9. Control Experiments

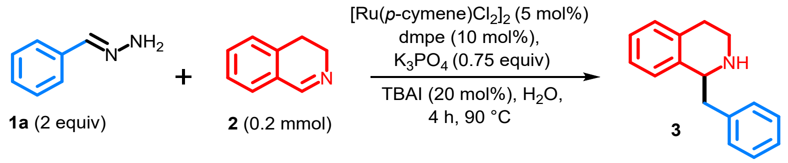

Entry	Condition	Yield 3 (%)
1	Without catalyst	3
2	Without ligand	0
3	Without catalyst +ligand	2.5
4	Without surfactant	70
5	Under air	73

a) Reaction conditions: Hydrazone **1a** (0.4 mmol, 2 equiv), 3,4-DHIQ **2** (0.2 mmol), [Ru(*p*-cymene)Cl_2]$_2$ (5 mol%), dmpe (10 mol%), K_3PO_4 (0.15 mmol, 0.75 equiv), and TBAI (20 mol%) in 1.0 mL H_2O at 90 °C for 4 h under Ar. ^{1}H NMR determined yields with 1,3,5-trimethoxybenzene as an internal standard.

Characterization data of compounds 3aa-3bn

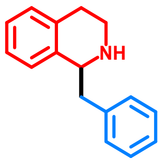

1-benzyl-1,2,3,4-tetrahydroisoquinoline (3aa) was prepared according to General Procedure (2.2), and the reaction was heated for 4 h. Yield, 80%, red oil. ^{1}H NMR (500 MHz, $CDCl_3$) δ 7.34 – 7.29 (m, 2H), 7.28 – 7.19 (m, 4H), 7.19 – 7.11 (m, 2H), 7.11 – 7.07 (m, 1H), 4.20 (dd, *J* = 10.2, 3.8 Hz, 1H), 3.30 – 3.16 (m, 2H), 2.96 – 2.73 (m, 4H), 2.04 (s, 1H). ^{13}C NMR (126 MHz, $CDCl_3$) δ 139.2, 138.6, 135.3, 129.4, 128.7, 126.6, 126.2, 125.8, 57.3, 42.6, 40.7, 30.0. HRMS (APCI) m/z calculated for $C_{16}H_{18}N$([M+H]$^+$): 224.1434, found: 224.1441.

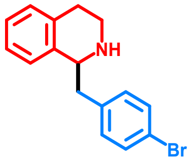

1-(4-bromobenzyl)-1,2,3,4-tetrahydroisoquinoline (3ab) was prepared according to General Procedure (2.2), and the reaction was heated for 8 h. Yield, 79%, red oil ^{1}H NMR (500 MHz, $CDCl_3$) δ 7.47 – 7.42 (m, 2H), 7.23 – 7.09 (m, 6H), 4.22 (dd, *J* = 9.8, 4.0 Hz, 1H), 3.25 – 3.17 (m, 2H), 2.99 – 2.74 (m, 4H), 2.22 (s, 1H). ^{13}C NMR (126 MHz, $CDCl_3$) δ 138.0, 137.9, 135.1 131.6, 131.1, 129.4, 126.3, 126.1, 125.8, 120.4, 57.0, 41.9, 40.6, 29.7. HRMS (ESI) m/z calculated for $C_{16}H_{17}BrN$([M+H]$^+$): 302.0539, found: 302.0534.

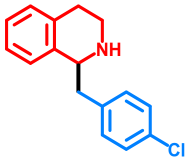

1-(4-chlorobenzyl)-1,2,3,4-tetrahydroisoquinoline (3ac) was prepared according to General Procedure (2.2), and the reaction was heated for 8 h. Yield, 76%, red oil. ^{1}H NMR (500 MHz, $CDCl_3$) δ 7.33 – 7.28 (m, 2H), 7.24 – 7.14 (m, 5H), 7.14 – 7.08 (m, 1H), 4.20 (dd, 1H), 3.28 – 3.17 (m, 2H), 3.00 – 2.71 (m, 4H), 1.98 (s, 1H). ^{13}C NMR (126 MHz, $CDCl_3$) δ 138.3, 137.6, 135.3, 132.3, 130.7, 129.4, 128.7, 126.3, 126.1, 125.7, 57.1, 41.9, 40.6, 29.9. HRMS (ESI) m/z calculated for $C_{16}H_{17}ClN([M+H]^+)$: 258.1044, found: 258.1039.

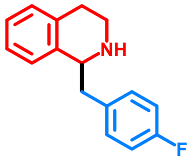

1-(4-fluorobenzyl)-1,2,3,4-tetrahydroisoquinoline (3ad) was prepared according to General Procedure (2.2), and the reaction was heated for 8 h. Yield, 65%, red oil. ^{1}H NMR (500 MHz, $CDCl_3$) δ 7.25 – 7.20 (m, 3H), 7.20 – 7.14 (m, 2H), 7.14 – 7.10 (m, 1H), 7.05 – 6.99 (m, 2H), 4.20 (dd, J = 10.0, 3.9 Hz, 1H), 3.27 – 3.17 (m, 2H), 2.97 – 2.74 (m, 4H), 1.89 (s, 1H). ^{13}C NMR (126 MHz, $CDCl_3$) δ 161. (d, J = 244.6 Hz), 138.2, 135.2, 134.7 (d, J = 3.2 Hz), 130.7(d, J = 7.8 Hz), 129.4, 126.2, 126.1, 125.7, 115.4(d, J = 21.1 Hz), 57.2, 41.7, 40.6, 29.8. ^{19}F NMR (471 MHz, $CDCl_3$) δ -116.63. HRMS (ESI) m/z calculated for $C_{16}H_{17}NF([M+H]^+)$: 242.1339, found: 242.1338.

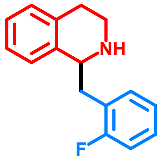

1-(2-fluorobenzyl)-1,2,3,4-tetrahydroisoquinoline (3ae) was prepared according to General Procedure (2.2), and the reaction was heated for 8 h, red oil. Yield, 63%. ^{1}H NMR (500 MHz, $CDCl_3$) δ 7.32 – 7.21 (m, 3H), 7.22 – 7.14 (m, 2H), 7.14 – 7.05 (m, 3H), 4.27 (dd, J = 10.4, 3.6 Hz, 1H), 3.34 – 3.18 (m, 2H), 3.03 – 2.69 (m, 4H), 1.83 (s, 1H). ^{13}C NMR (126 MHz, $CDCl_3$) δ 161.5 (d, J = 245.0 Hz), 138.6, 135.1, 131.7 (d, J = 5.0 Hz), 129.3, 128.2 (d, J = 8.2 Hz), 126.5, 126.4 (d, J = 15.7 Hz), 126.2, 125.7, 124.1 (d, J = 3.7 Hz), 115.4 (d, J = 22.4 Hz), 56.0, 40.0, 36.2, 29.8. ^{19}F NMR (471 MHz, $CDCl_3$) δ -117.79. HRMS (ESI) m/z calculated for $C_{16}H_{17}FN([M+H]^+)$: 242.1340, found: 242.1350.

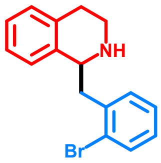

1-(2-bromobenzyl)-1,2,3,4-tetrahydroisoquinoline (3af) was prepared according to General Procedure (2.2), and the reaction was heated for 8 h. Yield, 70%, red solid. ^{1}H NMR (500 MHz, $CDCl_3$) δ 7.61 (d, *J* = 7.5 Hz, 1H), 7.37 – 7.33 (m, 1H), 7.31 – 7.26 (m, 2H), 7.22 – 7.09 (m, 4H), 4.36 (dd, *J* = 10.5, 3.5 Hz, 1H), 3.50 – 3.36 (m, 1H), 3.35 – 3.23 (m, 1H), 3.08 – 2.77 (m, 4H), 1.87 (s, 1H). ^{13}C NMR (126 MHz, $CDCl_3$) δ 138.7, 138.6, 135.1, 133.1, 131.9, 129.3, 128.2, 127.4, 126.5, 126.2, 125.8, 124.9, 55.1 42.9, 40.1, 29.9. HRMS (ESI) m/z calculated for $C_{16}H_{17}BrN([M+H]^+)$: 302.0539, Found: 302.0536.

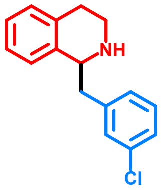

1-(3-chlorobenzyl)-1,2,3,4-tetrahydroisoquinoline (3ag) was prepared according to General Procedure (2.2), and the reaction was heated for 8 h. Yield, 76%, red oil. ^{1}H NMR (500 MHz, $CDCl_3$) δ 7.29 – 7.27 (m, 1H), 7.26 – 7.14 (m, 6H), 7.13 – 7.10 (m, 1H), 4.22 (dd, *J* = 10.1, 3.9 Hz, 1H), 3.31 – 3.17 (m, 2H), 3.00 – 2.71 (m, 4H), 2.05 (s, 1H). ^{13}C NMR (126 MHz, $CDCl_3$) δ 141.2, 138.1, 135.2, 134.4, 129.8, 129.4, 129.4, 127.5, 126.7, 126.3, 126.1, 125.8, 57.0, 42.3, 40.6, 29.8. HRMS (ESI) m/z calculated for $C_{16}H_{17}ClN([M+H]^+)$: 258.1044, found: 258.1037.

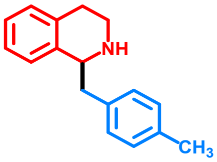

1-(4-methylbenzyl)-1,2,3,4-tetrahydroisoquinoline (3ah) was prepared according to General Procedure (2.2), and the reaction was heated for 8 h. Yield, 78%, red oil. ^{1}H NMR (500 MHz, $CDCl_3$) δ 7.29 – 7.27 (m, 1H), 7.22 – 7.09 (m, 7H), 4.20 (dd, *J* = 10.4, 3.7 Hz, 1H), 3.28 – 3.17 (m, 2H), 2.97 – 2.75 (m, 4H), 2.36 (s, 3H), 1.93 (s, 1H). ^{13}C NMR (126 MHz, $CDCl_3$) δ 138.7, 136.1, 136.0, 135.3, 129.4, 129.3, 129.2, 126.2, 126.1, 125.7, 57.3, 42.0, 40.7, 30.0, 21.0. HRMS (ESI) m/z calculated for $C_{17}H_{20}N([M+H]^+)$: 238.1590, found 238.1596.

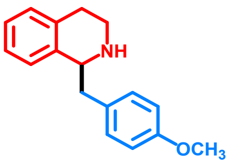

1-(4-methoxybenzyl)-1,2,3,4-tetrahydroisoquinoline (3ai) was prepared according to General Procedure (2.2), and the reaction was heated for 8 h. Yield, 76%, red oil. ^{1}H NMR (500 MHz, $CDCl_3$) δ 7.25 – 7.09 (m, 6H), 6.91 – 6.85 (m, 2H), 4.19 (dd, *J* = 10.0, 3.9 Hz, 1H), 3.81 (s, 3H), 3.27 – 3.18 (m, 2H), 2.98 – 2.75 (m, 4H), 2.13 (s, 1H). ^{13}C NMR (126 MHz, $CDCl_3$) δ 158.3, 138.4, 135.1, 130.9, 130.3, 129.3, 128.6, 126.2, 125.7, 114.0, 57.3, 55.3, 41.4, 40.6, 29.8. HRMS (APCI) m/z calculated for $C_{17}H_{20}ON([M+H]^+)$: 254.1539, found: 254.1536.

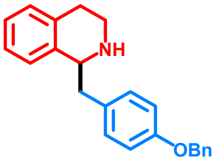

1-(4-(benzyloxy)benzyl)-1,2,3,4-tetrahydroisoquinoline (3aj) was prepared according to General Procedure (2.2), and the reaction was heated for 8 h. Yield, 57%, red oil. ^{1}H NMR (500 MHz, $CDCl_3$) δ 7.48 – 7.36 (m, 4H), 7.34 (d, *J* = 7.2 Hz, 1H), 7.26 – 7.22 (m, 1H), 7.21 – 7.14 (m, 4H), 7.13 – 7.09 (m, 1H), 6.95 (d, *J* = 8.8 Hz, 2H), 5.06 (s, 2H), 4.18 (dd, *J* = 10.1, 4.0 Hz, 1H), 3.26 – 3.16 (m, 2H), 2.98 – 2.73 (m, 4H), 1.83 (s, 1H). ^{13}C NMR (126 MHz, $CDCl_3$) δ 157.6, 137.0, 134.7, 132.8, 130.7, 130.4, 129.3, 128.6, 127.9, 127.5, 126.4, 126.3, 125.8, 115.0, 70.0, 57.0, 41.3, 40.4, 29.3. HRMS (APCI) m/z calculated for $C_{23}H_{24}ON([M+H]^+)$: 330.1852, found: 330.1858.

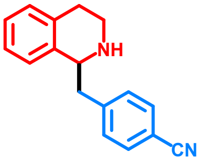

4-((1,2,3,4-tetrahydroisoquinolin-1-yl)methyl)benzonitrile (3ak) was prepared according to General Procedure (2.2), and the reaction was heated for 24 h. Yield, 65 %, red solid. ^{1}H NMR (500 MHz, $CDCl_3$) δ 7.62 (d, *J* = 8.4 Hz, 2H), 7.38 (d, *J* = 8.3 Hz, 2H), 7.22 – 7.15 (m, 3H), 7.14 – 7.09 (m, 1H), 4.25 (dd, *J* = 10.0, 4.0 Hz, 1H), 3.32 – 3.17 (m, 2H), 3.04 – 2.90 (m, 2H), 2.87 – 2.69 (m, 2H), 1.58 (s, 1H). ^{13}C NMR (126 MHz, $CDCl_3$) δ 145.1, 138.0, 135.3, 132.3, 130.2, 129.5, 126.4, 126.1, 125.8, 118.9, 110.3, 56.9, 42.9, 40.6, 29.8. HRMS (ESI) m/z calculated for $C_{17}H_{17}N_2([M+H]^+)$: 249.1386, found: 249.1384.

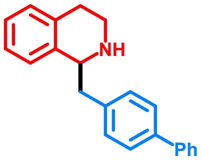

1-([1,1'-biphenyl]-4-ylmethyl)-1,2,3,4-tetrahydroisoquinoline (3al) was prepared according to General Procedure (2.2), and the reaction was heated for 8 h. Yield, 82%, red oil. ^{1}H NMR (500 MHz, $CDCl_3$) δ 7.63 – 7.54 (m, 4H), 7.44 (t, *J* = 7.8 Hz, 2H), 7.39 – 7.31 (m, 3H), 7.25 – 7.21 (m,

1H), 7.21 – 7.16 (m, 2H), 7.15 – 7.10 (m, 1H), 4.34 (dd, *J* = 9.7, 4.2 Hz, 1H), 3.37 – 3.23 (m, 2H), 3.08 – 2.95 (m, 2H), 2.96 – 2.79 (m, 2H), 2.51 (s, 1H). ^{13}C NMR (126 MHz, $CDCl_3$) δ 140.8, 139.5, 137.8, 137.7, 134.8, 129.8, 129.3, 128.7, 127.3, 127.2, 127.0, 126.4, 126.3, 125.8, 57.0, 42.0, 40.5, 29.4. HRMS (ESI) m/z calculated for $C_{22}H_{22}N([M+H]^+)$: 300.1747, found: 300.1737.

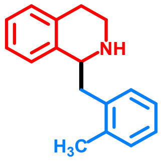

1-(2-methylbenzyl)-1,2,3,4-tetrahydroisoquinoline (3am) was prepared according to General Procedure (2.2), and the reaction was heated for 8 h. Yield, 75%, red oil.^{1}H NMR (500 MHz, $CDCl_3$) δ 7.34 – 7.26 (m, 5H), 7.26 – 7.19 (m, 3H), 4.30 (dd, *J* = 10.5, 3.7 Hz, 1H), 3.39 – 3.32 (m, 2H), 3.08 – 2.88 (m, 4H), 2.50 (s, 3H), 2.28 (s, 1H). ^{13}C NMR (126 MHz, $CDCl_3$) δ 138.8, 137.5, 136.6, 135.2, 130.6, 130.2, 129.4, 126.6, 126.3, 126.2, 126.1, 125.7, 55.8, 40.4, 39.9, 29.9, 19.7. HRMS (ESI) m/z calculated for $C_{17}H_{20}N([M+H]^+)$: 238.1590, found: 238.1583.

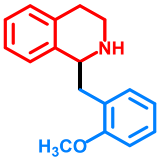

1-(2-methoxybenzyl)-1,2,3,4-tetrahydroisoquinoline (3an) was prepared according to General Procedure (2.2), and the reaction was heated for 8 h. Yield, 91%, red oil. ^{1}H NMR (500 MHz, $CDCl_3$) δ 7.33 – 7.28 (m, 1H), 7.28 – 7.24 (m, 1H), 7.22 – 7.14 (m, 3H), 7.14 – 7.08 (m, 1H), 6.96 – 6.90 (m, 2H), 4.30 (dd, *J* = 10.3, 3.5 Hz, 1H), 3.88 (s, 3H), 3.39 – 3.32 (m, 1H), 3.31 – 3.25 (m, 1H), 2.99 – 2.92 (m, 1H), 2.91 – 2.80 (m, 3H), 2.29 (s, 1H).^{13}C NMR (126 MHz, $CDCl_3$) δ 157.9, 139.2, 135.1, 131.3, 129.2, 127.9, 127.8, 126.8, 126.1, 125.7, 120.6, 110.5, 55.5, 55.4, 40.1, 37.6, 29.9. HRMS (ESI) m/z calculated for $C_{17}H_{20}NO([M+H]^+)$: 254.1539, found: 254.1533.

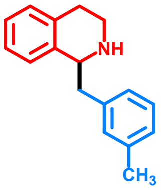

1-(3-methylbenzyl)-1,2,3,4-tetrahydroisoquinoline (3ao) was prepared according to General Procedure (2.2), and the reaction was heated for 8 h. Yield, 76%, red oil. ^{1}H NMR (500 MHz, $CDCl_3$) δ 7.25 – 7.21 (m, 2H), 7.20 – 7.15 (m, 2H), 7.14 – 7.06 (m, 4H), 4.25 (dd, *J* = 10.1, 3.9 Hz, 1H), 3.29 – 3.20 (m, 2H), 2.99 – 2.77 (m, 4H), 2.46 (s, 1H), 2.36 (s, 3H). ^{13}C NMR (126 MHz, $CDCl_3$) δ 138.6, 138.1, 138.0, 134.8, 130.0, 129.1, 128.4, 127.1, 126.2, 126.1, 126.1, 125.6, 57.0, 42.2, 40.4, 29.5, 21.3. HRMS (ESI) m/z calculated for $C_{17}H_{20}N([M+H]^+)$: 238.1590, found: 238.1579.

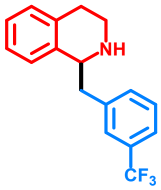

1-(3-(trifluoromethyl)benzyl)-1,2,3,4-tetrahydroisoquinoline (3ap) was prepared according to General Procedure (2.2), and the reaction was heated for 8 h. Yield, 68%, red oil. ^{1}H NMR (500 MHz, $CDCl_3$) δ 7.55 – 7.41 (m, 4H), 7.25 – 7.08 (m, 4H), 4.25 (dd, *J* = 10.1, 3.8 Hz, 1H), 3.36 – 3.18 (m, 2H), 3.06 – 2.74 (m, 4H), 1.79 (s, 1H). ^{13}C NMR (126 MHz, $CDCl_3$) δ 140.2, 138.1, 135.3, 132.8, 130.8 (q, *J* = 32.0 Hz), 129.4, 128.9, 126.3, 126.1, 126.0 (q, *J* = 3.8 Hz), 125.8, 125.2 (q, *J* = 272.3 Hz), 123.4 (q, *J* = 3.9 Hz), 57.0, 42.5, 40.6, 29.8. ^{19}F NMR (471 MHz, $CDCl_3$) δ -62.51. HRMS (APCI) m/z calculated for $C_{17}H_{17}NF_3$([M+H]$^+$): 292.1307, found: 292.1304.

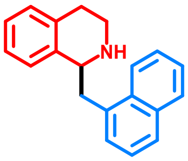

1-(naphthalen-1-ylmethyl)-1,2,3,4-tetrahydroisoquinoline (3as) was prepared according to General Procedure (2.2), and the reaction was heated for 24 h. Yield, 59%, red oil. ^{1}H NMR (500 MHz, $CDCl_3$) δ 8.06 (d, *J* = 8.9 Hz, 1H), 7.73 (dd, *J* = 8.1, 1.6 Hz, 1H), 7.62 (d, *J* = 7.8 Hz, 1H), 7.42 – 7.34 (m, 2H), 7.34 – 7.27 (m, 1H), 7.27 – 7.22 (m, 2H), 7.10 – 7.02 (m, 2H), 7.01 – 6.97 (m, 1H), 4.23 (dd, *J* = 10.4, 3.5 Hz, 1H), 3.69 (dd, *J* = 13.9, 3.6 Hz, 1H), 3.11 – 3.03 (m, 2H), 2.80 – 2.61 (m, 3H), 1.57 (s, 1H). ^{13}C NMR (126 MHz, $CDCl_3$) δ 138.8, 135.2, 135.0, 134.1, 132.0, 129.4, 128.9, 127.8, 127.4, 126.3, 126.2, 126.0, 125.7, 125.7, 125.5, 123.7, 56.1, 40.6, 39.7, 29.9. HRMS (ESI) m/z calculated for $C_{20}H_{20}N$([M+H]$^+$): 274.1430, found: 274.1584.

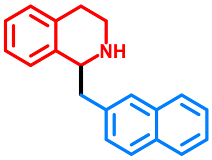

1-(naphthalen-2-ylmethyl)-1,2,3,4-tetrahydroisoquinoline (3at) was prepared according to General Procedure (2.2), and the reaction was heated for 8 h. Yield, 91%, red oil. ^{1}H NMR (500 MHz, $CDCl_3$) δ 7.88 – 7.77 (m, 3H), 7.74 (s, 1H), 7.51 – 7.41 (m, 3H), 7.31 – 7.27 (m, 1H), 7.24 – 7.16 (m, 2H), 7.16 – 7.12 (m, 1H), 4.37 (dd, *J* = 10.0, 4.0 Hz, 1H), 3.47 (dd, *J* = 13.7, 4.0 Hz, 1H), 3.29 – 3.20 (m, 1H), 3.16 – 3.07 (m, 1H), 3.00 – 2.80 (m, 3H), 2.74 (s, 1H). ^{13}C NMR (126 MHz, $CDCl_3$) δ 138.1, 136.4, 135.1, 133.6, 132.3, 129.4, 128.3, 128.0, 127.7, 127.6, 127.5, 126.3, 126.2, 126.1, 125.8, 125.5, 57.0, 42.6, 40.7, 29.6. HRMS (ESI) m/z calculated for $C_{20}H_{20}N$([M+H]$^+$): 274.1590, found: 274.1584.

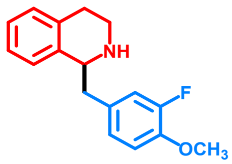

1-(3-fluoro-4-methoxybenzyl)-1,2,3,4-tetrahydroisoquinoline (3au) was prepared according to General Procedure (2.2), and the reaction was heated for 8 h. Yield, 55%, red oil. ^{1}H NMR (500 MHz, $CDCl_3$) δ 7.20 – 7.09 (m, 4H), 7.01 – 6.95 (m, 2H), 6.94 – 6.89 (m, 1H), 4.26 (dd, 1H), 3.88 (s, 3H), 3.27 – 3.17 (m, 2H), 3.06 – 2.91 (m, 2H), 2.90 – 2.78 (m, 2H). δ ^{13}C NMR (126 MHz, $CDCl_3$) δ 152.3 (d, *J* = 245.8 Hz), 146.3 (d, *J* = 10.5 Hz), 137.1, 134.6, 131.4 (d, *J* = 6.0 Hz), 129.4, 126.5, 126.2, 125.9, 125.1 (d, *J* = 3.4 Hz), 117.0 (d, *J* = 18.0 Hz), 113.5 (d, *J* = 2.3 Hz), 56.8, 56.3, 41.2, 40.41, 29.1. ^{19}F NMR (471 MHz, $CDCl_3$) δ -134.95 – -135.01 (m, 1F). HRMS (APCI) m/z calculated for $C_{17}H_{19}FNO([M+H]^+)$: 272.1445, found: 272.1442.

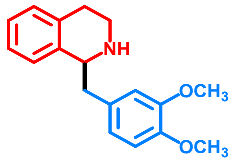

1-(3,4-dimethoxybenzyl)-1,2,3,4-tetrahydroisoquinoline (3av) was prepared according to General Procedure (2.2), and the reaction was heated for 8 h. Yield, 41%, red oil. ^{1}H NMR (500 MHz, $CDCl_3$) δ 7.28 – 7.22 (m, 1H), 7.21 – 7.13 (m, 2H), 7.13 – 7.08 (m, 1H), 6.85 – 6.79 (m, 2H), 6.73 (d, *J* = 1.8 Hz, 1H), 4.23 (dd, *J* = 9.6, 3.9 Hz, 1H), 3.87 (s, 3H), 3.84 (s, 3H), 3.28 – 3.18 (m, 2H), 3.00 – 2.85 (m, 2H), 2.87 – 2.73 (m, 2H), 1.87 (s, 1H).^{13}C NMR (126 MHz, $CDCl_3$) δ 148.9, 147.7, 138.0, 135.2, 131.1, 129.3, 126.2, 126.2, 125.7, 121.4, 112.4, 111.3, 57.1, 55.9, 55.9, 41.9, 40.8, 29.7. HRMS (APCI) m/z calculated for $C_{18}H_{22}O_2N([M+H]^+)$: 284.1645, found: 284.1642.

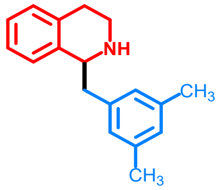

1-(2,4-dimethylbenzyl)-1,2,3,4-tetrahydroisoquinoline (3aw) was prepared according to General Procedure (2.2), and the reaction was heated for 8 h, red oil. Yield, 72%. ^{1}H NMR (500 MHz, $CDCl_3$) δ 7.29 – 7.26 (m, 1H), 7.22 – 7.10 (m, 3H), 6.93 – 6.87 (m, 3H), 4.20 (dd, *J* = 10.4, 3.8 Hz, 1H), 3.28 – 3.18 (m, 2H), 2.98 – 2.74 (m, 4H), 2.32 (s, 6H), 1.92 (s, 1H).^{13}C NMR (126 MHz, $CDCl_3$) δ 139.0, 138.7, 138.1, 135.2, 129.3, 128.1, 127.1, 126.1, 126.1, 125.7, 57.3, 42.3, 40.7, 29.9, 21.3. HRMS (APCI) m/z calculated for $C_{18}H_{22}N([M+H]^+)$: 252.1746, found: 252.1743.

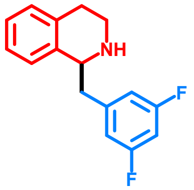

1-(3,5-difluorobenzyl)-1,2,3,4-tetrahydroisoquinoline(3ax) was prepared according to General Procedure (2.2), and the reaction was heated for 8 h. Yield, 60%, red solid. ^{1}H NMR (500 MHz, $CDCl_3$) δ 7.20 – 7.14 (m, 3H), 7.15 – 7.10 (m, 1H), 6.85 – 6.78 (m, 2H), 6.74 – 6.66 (m, 1H), 4.23 (dd, *J* = 10.1, 3.8 Hz, 1H), 3.27 – 3.16 (m, 2H), 3.02 – 2.72 (m, 4H), 1.70 (s, 1H). ^{13}C NMR (126 MHz, $CDCl_3$) δ 163.0 (dd, *J* = 248.4, 13.0 Hz), 143.2 (t, *J* = 9.0 Hz), 137.9, 135.3, 129.5, 126.4, 126.1, 125.8, 112.12 (dd, *J* = 13.3, 5.6 Hz), 102.0 (t, *J* = 25.3 Hz), 56.8, 42.4 (t, *J* = 2.1 Hz), 40.5, 29.8. ^{19}F NMR (471 MHz, $CDCl_3$) δ -62.51 (s, 2F). HRMS (APCI) m/z calculated for $C_{16}H_{16}F_2N([M+H]^+)$: 260.1245, found: 260.1253.

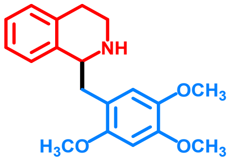

1-(2,4,5-trimethoxybenzyl)-1,2,3,4-tetrahydroisoquinoline (3ay) was prepared according to General Procedure (2.2), and the reaction was heated for 8 h. Yield, 60%, red oil. ^{1}H NMR (500 MHz, $CDCl_3$) δ 7.26 – 7.22 (m, 1H), 7.20 – 7.11 (m, 2H), 7.12 – 7.06 (m, 1H), 6.72 (s, 1H), 6.55 (s, 1H), 4.33 (dd, *J* = 9.8, 3.8 Hz, 1H), 3.89 (s, 3H), 3.83 (s, 3H), 3.81 (s, 3H), 3.32 – 3.25 (m, 2H), 3.02 – 2.93 (m, 1H), 2.88 – 2.81 (m, 3H), 2.47 (s, 1H). ^{13}C NMR (126 MHz, $CDCl_3$) δ 151.9, 148.3, 142.8, 134.7, 132.1, 129.1, 126.6, 126.2, 125.8, 115.2, 97.7, 56.6, 56.3, 56.2, 55.8, 40.3, 36.8, 29.3. HRMS (ESI) m/z calculated for $C_{19}H_{24}O_3N([M+H]^+)$: 314.1750, found: 314.1749.

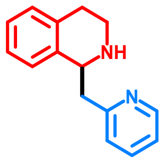

1-(pyridin-2-ylmethyl)-1,2,3,4-tetrahydroisoquinoline (3az) was prepared according to General Procedure (2.2), and the reaction was heated for 24 h. Yield, 56%, red oil. ^{1}H NMR (500 MHz, $CDCl_3$) δ 8.56 (d, *J* = 3.9 Hz, 1H), 7.62 (td, *J* = 7.6, 1.8 Hz, 1H), 7.24 – 7.21 (m, 1H), 7.21 – 7.13 (m, 4H), 7.12 – 7.08 (m, 1H), 4.63 (dd, *J* = 9.7, 3.5 Hz, 1H), 3.48 – 3.40 (m, 1H), 3.35 – 3.27 (m, 1H), 3.24 – 3.13 (m, 1H), 3.10 – 3.01 (m, 1H), 2.95 (s, 1H), 2.91 – 2.78 (m, 2H). ^{13}C NMR (126 MHz, $CDCl_3$) δ 159.2, 149.2, 137.3, 136.7, 134.8, 129.3, 126.4, 126.2, 126.0, 124.2, 121.7, 55.6, 43.4, 40.3, 29.2. HRMS (ESI) m/z calculated for $C_{15}H_{17}N_2([M+H]^+)$: 225.1386, found: 225.1376.

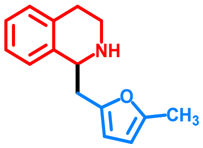

1-((5-methylfuran-2-yl)methyl)-1,2,3,4-tetrahydroisoquinoline (3ba) was prepared according to General Procedure (2.2), and the reaction was heated for 8 h. Yield, 57%, red solid. ^{1}H NMR (500 MHz, $CDCl_3$) δ 7.18 – 7.07 (m, 4H), 5.98 (d, J = 3.0 Hz, 1H), 5.88 (d, J = 1.1 Hz, 1H), 4.31 (dd, J = 9.8, 3.9 Hz, 1H), 3.28 – 3.20 (m, 1H), 3.20 – 3.13 (m, 1H), 3.04 – 2.93 (m, 2H), 2.92 – 2.76 (m, 2H), 2.40 (s, 1H), 2.28 (s, 3H). ^{13}C NMR (126 MHz, $CDCl_3$) δ 151.1, 151.1, 137.7, 135.1, 129.2, 126.3, 126.2, 125.7, 108.0, 106.1, 54.8, 40.3, 35.1, 29.6, 13.6. HRMS (APCI) m/z calculated for $C_{15}H_{18}NO([M+H]^+)$: 228.1383, found; 228.1377.

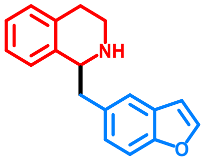

1-(benzofuran-5-ylmethyl)-1,2,3,4-tetrahydroisoquinoline (3bb) was prepared according to General Procedure (2.2), and the reaction was heated for 8 h. Yield, 60%, red oil. ^{1}H NMR (500 MHz, $CDCl_3$) δ 7.62 (d, J = 2.1 Hz, 1H), 7.52 – 7.49 (m, 1H), 7.47 (d, J = 8.4 Hz, 1H), 7.32 – 7.27 (m, 1H), 7.23 – 7.15 (m, 3H), 7.15 – 7.10 (m, 1H), 6.74 (dd, J = 2.2, 0.9 Hz, 1H), 4.25 (dd, J = 10.3, 3.8 Hz, 1H), 3.54 – 3.32 (m, 1H), 3.29 – 3.19 (m, 1H), 3.06 – 2.72 (m, 4H), 1.92 (s, 1H). ^{13}C NMR (126 MHz, $CDCl_3$) δ 153.9, 145.3, 138.6, 135.2, 133.4, 129.3, 127.7, 126.1, 126.1, 125.7, 125.5, 121.6, 111.4, 106.4, 57.6, 42.3, 40.7, 29.9. HRMS (APCI) m/z calculated for $C_{18}H_{18}ON([M+H]^+)$: 264.1382, found: 264.1381.

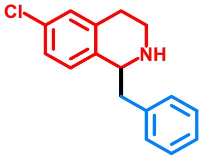

1-benzyl-6-chloro-1,2,3,4-tetrahydroisoquinoline (3be) was prepared according to General Procedure (2.2), and the reaction was heated for 6 h. Yield, 70%, red oil. ^{1}H NMR (500 MHz, $CDCl_3$) δ 7.37 – 7.30 (m, 2H), 7.28 – 7.24 (m, 3H), 7.18 – 7.12 (m, 2H), 7.11 (s, 1H), 4.17 (dd, J = 10.2, 3.9 Hz, 1H), 3.29 – 3.12 (m, 2H), 2.99 – 2.67 (m, 4H), 1.66 (s, 1H). ^{13}C NMR (126 MHz, $CDCl_3$) δ 138.7, 137.3, 137.1, 131.6, 129.3, 129.0, 128.7, 127.6, 126.6, 125.8, 56.9, 42.4, 40.3, 29.9. HRMS (ESI) m/z calculated for $C_{16}H_{17}ClN([M+H]^+)$: 258.1044, found: 258.1046.

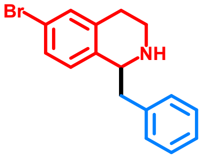

1-benzyl-6-bromo-1,2,3,4-tetrahydroisoquinoline (3bf) was prepared according to General Procedure (2.2), and the reaction was heated for 6 h. Yield, 78%, red oil. ^{1}H NMR (500 MHz, $CDCl_3$) δ 7.36 – 7.31 (m, 2H), 7.30 – 7.27 (m, 2H), 7.27 – 7.23 (m, 3H), 7.04 (d, *J* = 8.7 Hz, 1H), 4.22 (dd, *J* = 9.4, 4.4 Hz, 1H), 3.26 – 3.16 (m, 2H), 3.00 – 2.91 (m, 2H), 2.91 – 2.77 (m, 2H). ^{13}C NMR (126 MHz, $CDCl_3$) δ 138.4, 137.4, 137.1, 132.0, 129.3, 128.8, 128.7, 128.0, 126.7, 119.9, 56.8, 42.2, 40.2, 29.5. HRMS (ESI) m/z calculated for $C_{16}H_{17}NBr([M+H]^+)$: 302.0538, found: 302.0536.

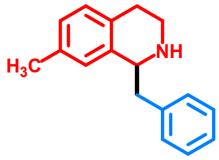

1-benzyl-7-methyl-1,2,3,4-tetrahydroisoquinoline (3bg) was prepared according to General Procedure (2.2), and the reaction was heated to 6 h. Yield, 75%, red oil. ^{1}H NMR (500 MHz, $CDCl_3$) δ 7.38 – 7.32 (m, 2H), 7.30 – 7.22 (m, 3H), 7.06 (s, 1H), 7.03 – 6.97 (m, 2H), 4.19 (dd, *J* = 10.3, 3.7 Hz, 1H), 3.33 – 3.11 (m, 2H), 2.98 – 2.69 (m, 4H), 2.33 (s, 3H), 2.01 (s, 1H). ^{13}C NMR (126 MHz, $CDCl_3$) δ 139.2, 135.1, 132.1, 129.3, 129.2, 128.6, 128.6, 127.0, 126.7, 126.4, 57.2, 42.5, 40.7, 29.5, 21.2. HRMS (ESI) m/z calculated for $C_{17}H_{20}N([M+H]^+)$: 238.1590, found: 238.1583.

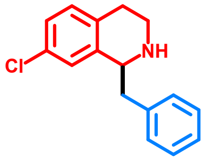

1-benzyl-7-chloro-1,2,3,4-tetrahydroisoquinoline (3bh) was prepared according to General Procedure (2.2), and the reaction was heated for 6 h. Yield, 67%, red oil. ^{1}H NMR (500 MHz, $CDCl_3$) δ 7.38 – 7.32 (m, 2H), 7.28 – 7.25 (m, 2H), 7.24 – 7.21 (m, 2H), 7.15 – 7.11 (m, 1H), 7.05 (d, *J* = 8.1 Hz, 1H), 4.17 (dd, *J* = 10.2, 3.9 Hz, 1H), 3.27 – 3.17 (m, 2H), 2.93 – 2.69 (m, 4H), 1.77 (s, 1H). ^{13}C NMR (126 MHz, $CDCl_3$) δ 140.3, 138.6, 133.7, 131.2, 130.7, 129.3, 128.7, 126.6, 126.3, 126.1, 57.1, 42.3, 40.5, 29.4. HRMS (APCI) m/z calculated for $C_{16}H_{17}ClN([M+H]^+)$: 258.1044, found: 258.1051.

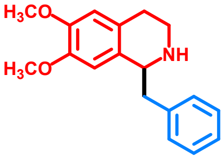

1-benzyl-6,7-dimethoxy-1,2,3,4-tetrahydroisoquinoline (3bk) was prepared according to General Procedure (2.2), and the reaction was heated for 6 h. Yield, 90%, red oil. ^{1}H NMR (500 MHz, $CDCl_3$) δ 7.36 – 7.31 (m, 2H), 7.29 – 7.22 (m, 3H), 6.59 (s, 2H), 4.18 (dd, *J* = 9.3, 4.7 Hz,

1H), 3.86 (s, 3H), 3.79 (s, 3H), 3.27 – 3.17 (m, 2H), 2.99 – 2.90 (m, 2H), 2.84 – 2.66 (m, 2H), 1.81 (s, 1H).^{13}C NMR (126 MHz, $CDCl_3$) δ 147.7, 147.0, 138.5, 129.5, 128.9, 128.6, 126.6, 126.5, 111.7, 109.5, 56.6, 55.8, 42.4, 40.2, 28.6. HRMS (ESI) m/z calculated for $C_{18}H_{22}NO_2$ ([M+H]$^+$): 284.1645, found: 284.1648.

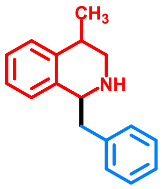

1-benzyl-4-methyl-1,2,3,4-tetrahydroisoquinoline (3bl) was prepared according to General Procedure (2.2), and the reaction was heated for 6 h. Yield, 73%, red oil. ^{1}H NMR (500 MHz, $CDCl_3$) δ 7.34 – 7.30 (m, 2H), 7.29 – 7.22 (m, 5H), 7.19 (ddd, J = 8.7, 4.4, 2.3 Hz, 2H), 4.23 (dd, J = 9.4, 4.4 Hz, 1H), 3.32 – 3.16 (m, 1H), 3.06 – 2.96 (m, 1H), 2.97 – 2.82 (m, 2H), 2.67 – 2.55 (m, 1H), 1.79 (s, 1H), 1.29 – 1.21 (m, 3H). ^{13}C NMR (126 MHz, $CDCl_3$) δ 140.7, 139.0, 138.3, 129.4, 128.5, 128.2, 126.4, 126.2, 126.0, 125.6, 57.4, 47.4, 42.5, 32.9, 20.5. HRMS (ESI) m/z calculated for $C_{17}H_{20}N$([M+H]$^+$): 238.1583, found: 238.1590.

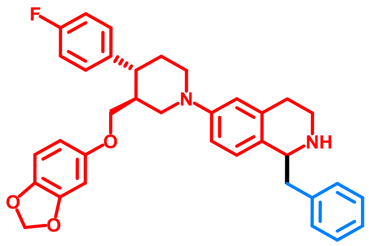

6-((3S,4R)-3-((benzo[d][1,3]dioxol-5-yloxy)methyl)-4-(4-fluorophenyl)piperidin-1-yl)-1-benzyl-1,2,3,4-tetrahydroisoquinoline (Paroxetine derivative) 3bm was prepared according to General Procedure (2.2), and the reaction was heated for 8 h, and 3 equiv of benzaldehyde hydrazone was added. Yield, 64%, red oil, dr value is 1:1 which is calculated by LCMS. ^{1}H NMR (500 MHz, $CDCl_3$) δ 7.33 (t, J = 7.5 Hz, 2H), 7.30 – 7.26 (m, 3H), 7.23 – 7.14 (m, 3H), 7.00 (t, J = 8.5 Hz, 2H), 6.88 (d, J = 9.5 Hz, 1H), 6.74 (s, 1H), 6.64 (d, J = 8.4 Hz, 1H), 6.38 (d, J = 2.5 Hz, 1H), 6.17 (dd, J = 8.6, 2.5 Hz, 1H), 5.89 (s, 2H), 4.17 (dd, J = 10.2, 3.7 Hz, 1H), 4.00 (dd, J = 12.3 Hz, 1H), 3.79 (d, J = 12.4 Hz, 1H), 3.70 – 3.62 (m, 1H), 3.57 – 3.50 (m, 1H), 3.32 – 3.17 (m, 2H), 2.97 – 2.81 (m, 4H), 2.81 – 2.72 (m, 1H), 2.61 (dt, J = 11.6, 4.3 Hz, 1H), 2.37 – 2.27 (m, 1H), 2.06 – 1.90 (m, 3H). ^{13}C NMR (126 MHz, $CDCl_3$) δ 161.6(d, J = 244.4 Hz), 154.3, 149.8, 148.2, 141.6, 139.4(d, J = 3.10 Hz), 139.2, 135.9, 129.4, 128.8(d, J = 7.6 Hz), 128.8, 128.6, 126.8, 126.4, 115.5 (d, J = 21.0 Hz), 115.1, 115.0, 107.8, 105.6, 101.1, 98.0, 69.4, 56.8, 53.8, 50.8, 44.1, 42.5, 41.8, 41.0, 34.1, 30.3. ^{19}F NMR (471 MHz, $CDCl_3$) δ -116.28. HRMS (ESI) m/z calculated for $C_{35}H_{36}FN_2O_3$([M+H]$^+$): 551.2704, found: 551.2716.

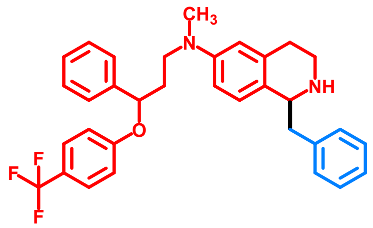

1-benzyl-N-methyl-N-(3-phenyl-3-(4-(trifluoromethyl)phenoxy)propyl)-1,2,3,4-tetrahydroisoquinolin-6-amine (Fluoxetine derivative) 3bn was prepared according to General Procedure (2.2), and the reaction was heated for 8 h, and 3 equiv of benzaldehyde hydrazone was added. Yield, 67%, red oil. ^{1}H NMR (500 MHz, $CDCl_3$) δ 7.44 (d, *J* = 8.5 Hz, 2H), 7.33 (d, *J* = 6.7 Hz, 6H), 7.27 (d, *J* = 6.8 Hz, 2H), 7.25 – 7.22 (m, 2H), 7.05 (dd, *J* = 8.7, 4.4 Hz, 1H), 6.90 (d, *J* = 8.5 Hz, 2H), 6.56 (dd, *J* = 8.6, 2.7 Hz, 1H), 6.37 (s, 1H), 5.22 (dd, *J* = 8.5, 4.2 Hz, 1H), 4.15 – 4.09 (m, 1H), 3.65 – 3.56 (m, 1H), 3.51 – 3.38 (m, 1H), 3.23 (dt, *J* = 13.6, 3.7 Hz, 1H), 3.19 – 3.09 (m, 1H), 3.05 – 2.95 (m, 1H), 2.90 (s, 3H), 2.89 – 2.69 (m, 2H), 2.64 (t, *J* = 5.9 Hz, 1H), 2.22 – 2.11 (m, 2H). ^{13}C NMR (126 MHz, $CDCl_3$) δ 160.4, 147.7, 140.8, 139.1, 135.8, 129.4, 128.9, 128.8, 128.5, 127.9, 126.9, 126.8 (q, *J* = 2.95 Hz), 126.4, 125.7, 125.6 (q, *J* = 270.1 Hz), 123.0 (q, *J* = 32.62 Hz), 115.7, 112.5, 110.5, 77.8, 56.7, 49.1, 42.5, 41.0, 38.3, 36.2, 30.0. ^{19}F NMR (471 MHz, $CDCl_3$) δ -61.61. HRMS (ESI) m/z calculated for $C_{33}H_{34}F_3N_2O([M+H]^+)$: 531.2618, found: 531.2611.

References:

1. C.-C. Li, X.-J. Dai, H. Wang, D. Zhu, J. Gao and C.-J. Li, *Org. Lett.*, **2018**, *20*, 3801–3805.
2. Sangster, J. J.; Ruscoe, R. E.; Cosgrove, S. C.; Mangas-Sanchez, J.; Turner, N. J., *J. Am. Chem. Soc.* **2023**, *145 (8),* 4431–4437.
3. Lalonde, M. P.; McGowan, M. A.; Rajapaksa, N. S.; Jacobsen, E. N., *J. Am. Chem. Soc.* **2013**, *135* (5), 1891–1894.
4. Choudhury, A. R.; Mukherjee, S., *Chem. Sci.* **2016**, *7* (12), 6940–6945.
5. Xie, J. H.; Yan, P. C.; Zhang, Q. Q.; Yuan, K. X.; Zhou, Q. L., *ACS Catal.* **2012**, *2* (4), 561–564.
6. Ooi, T.; Kameda, M.; Taniguchi, M.; Maruoka, K., *Eur. J. Org. Chem.* **2005**, *2005* (6), 1135–1138.

NMR spectra collection for compounds

^{1}H NMR (500 MHz, $CDCl_3$) spectrum of **2d**

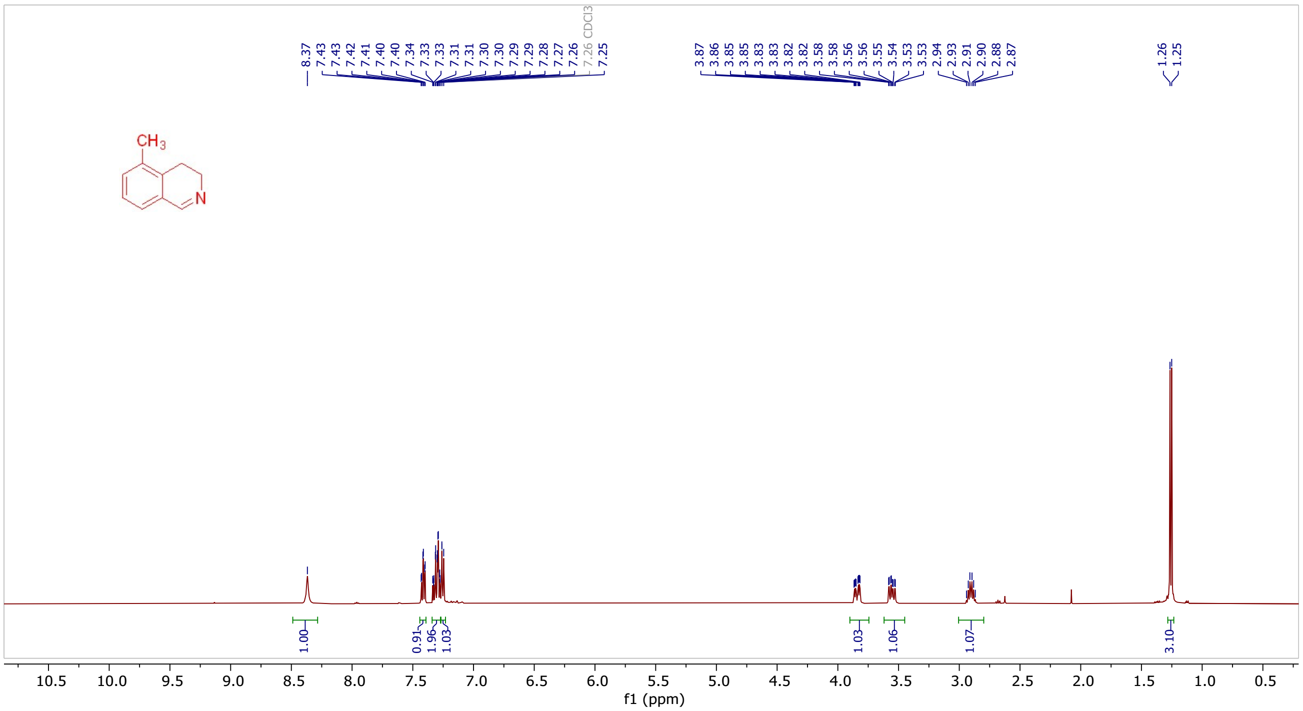

$^{13}C\{^{1}H\}$ NMR (126 MHz, $CDCl_3$) spectrum of **2d**

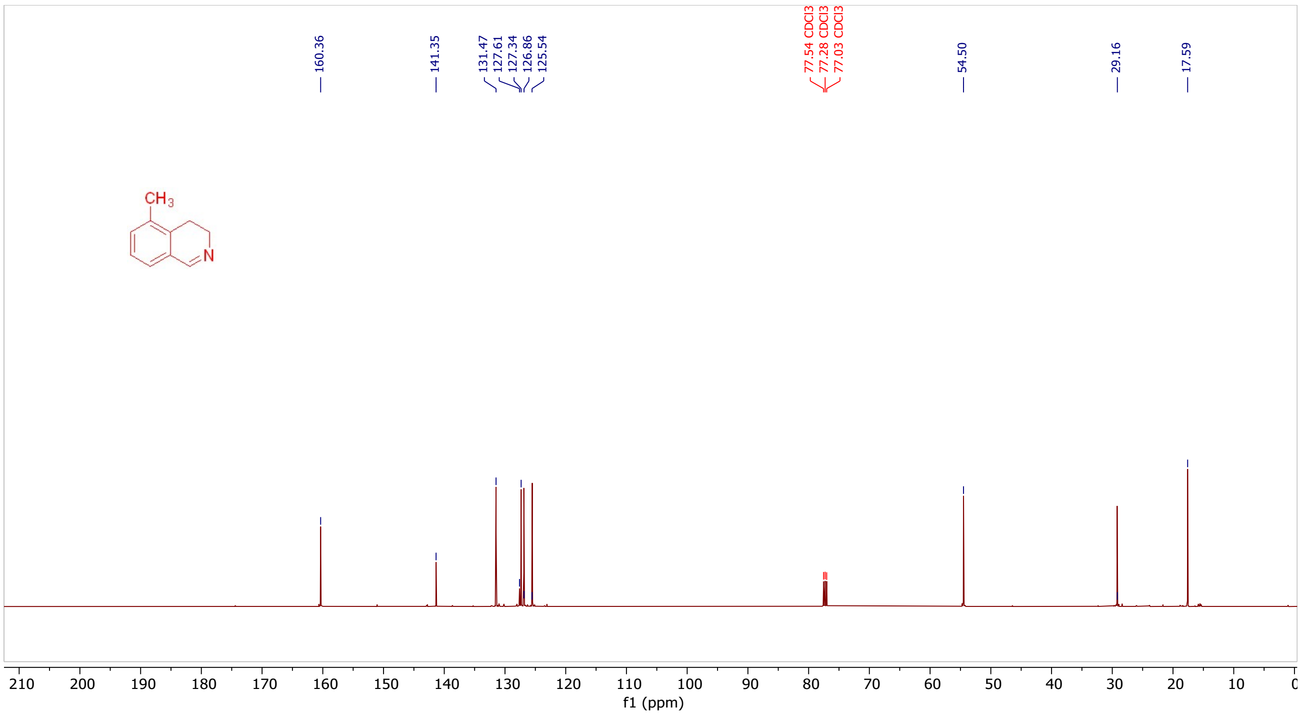

^{1}H NMR (500 MHz, $CDCl_3$) spectrum of **2e**

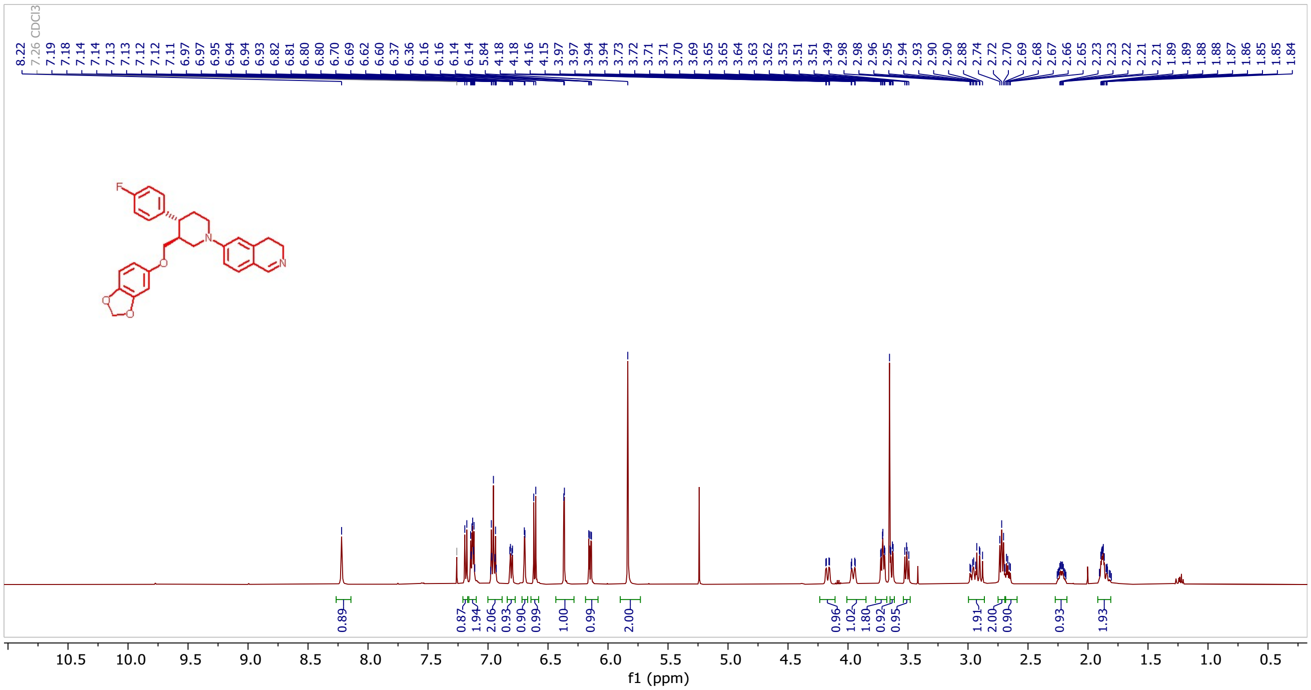

^{13}C{^{1}H} NMR (126 MHz, $CDCl_3$) spectrum of **2e**

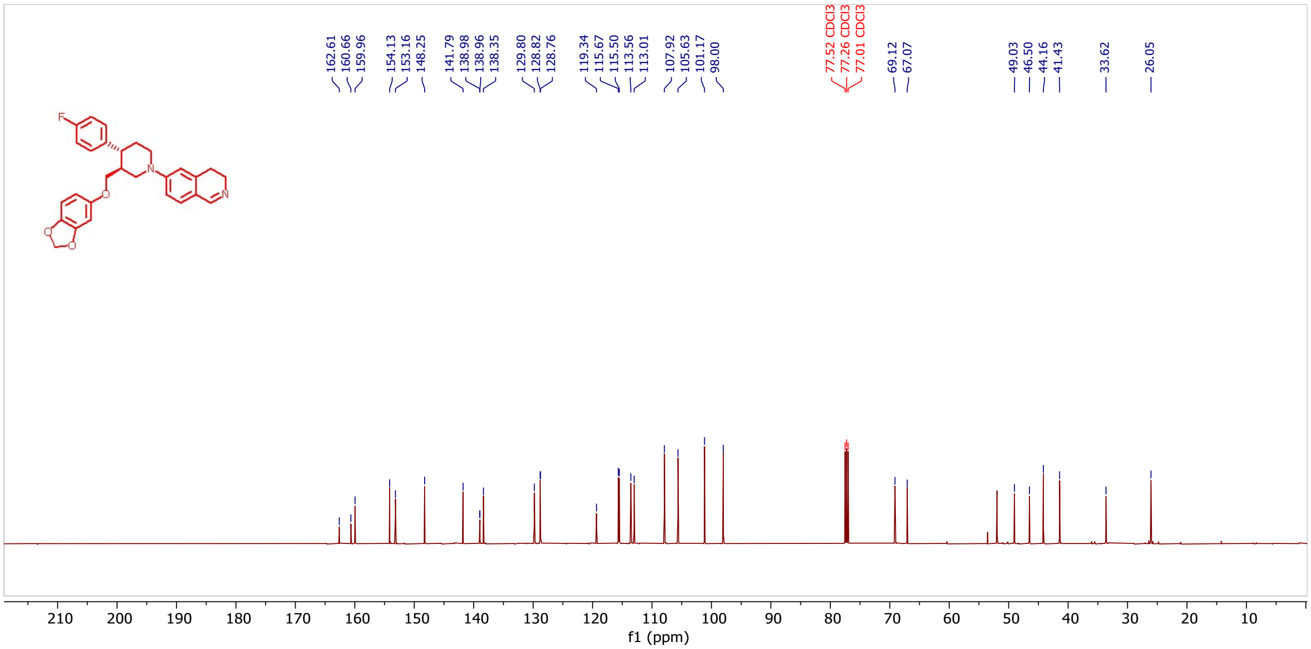

^{19}F NMR (471 MHz, $CDCl_3$) spectrum of **2e**

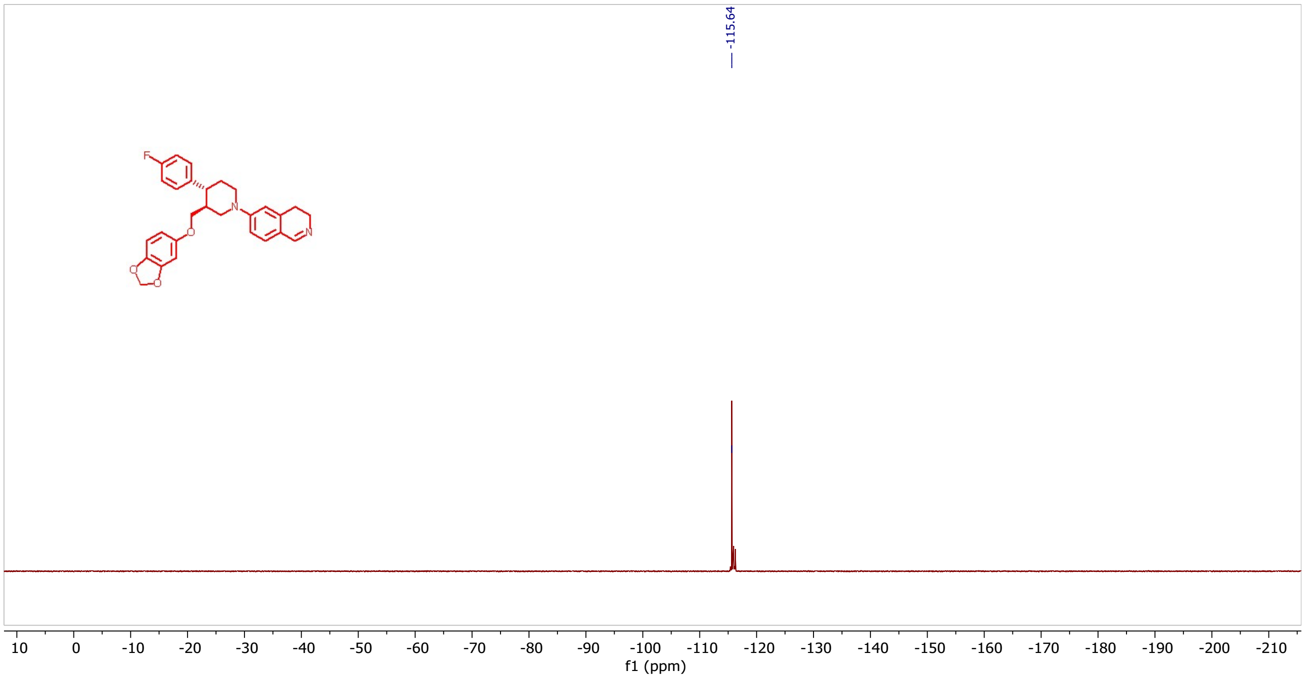

^{1}H NMR (500 MHz, $CDCl_3$) spectrum of **2f**

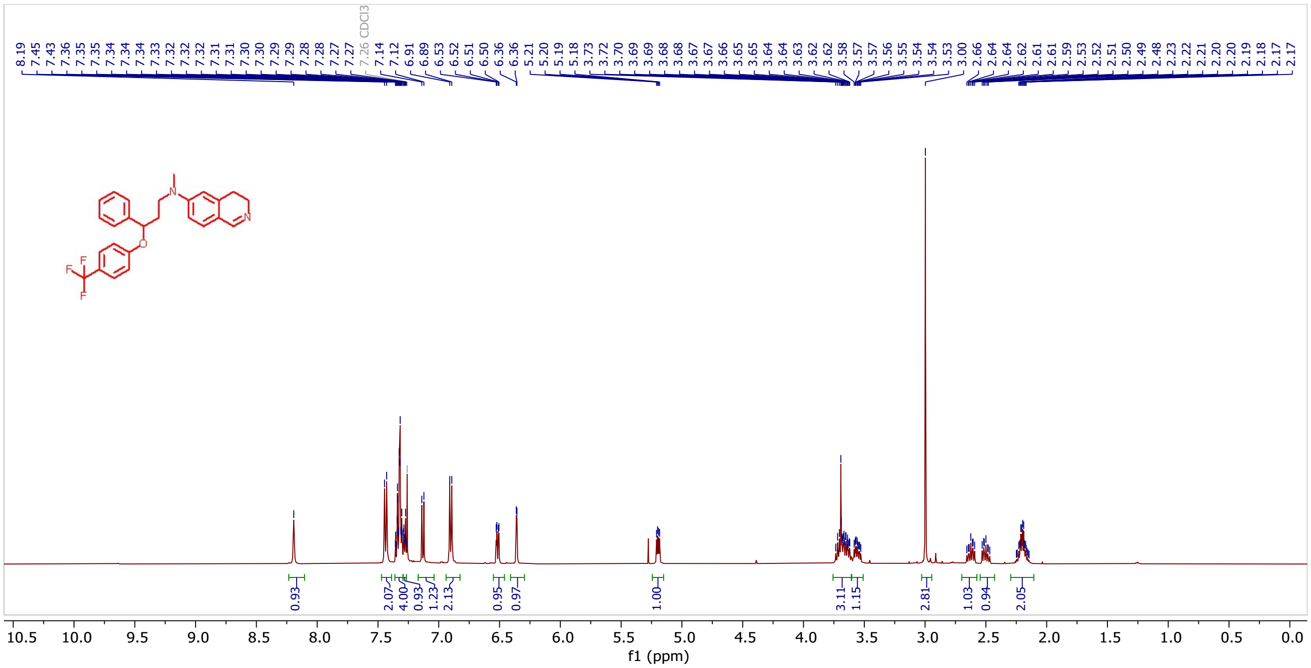

$^{13}C\{^{1}H\}$ NMR (126 MHz, $CDCl_3$) spectrum of **2f**

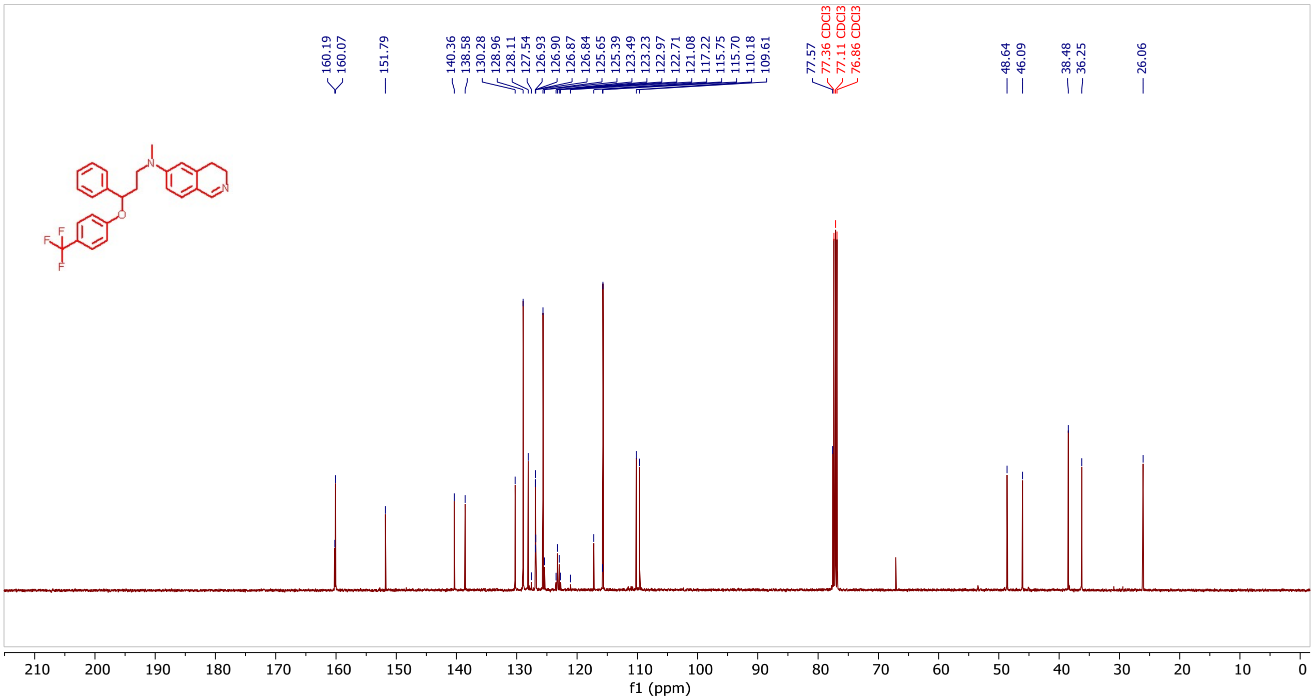

^{19}F NMR (471 MHz, $CDCl_3$) spectrum of **2f**

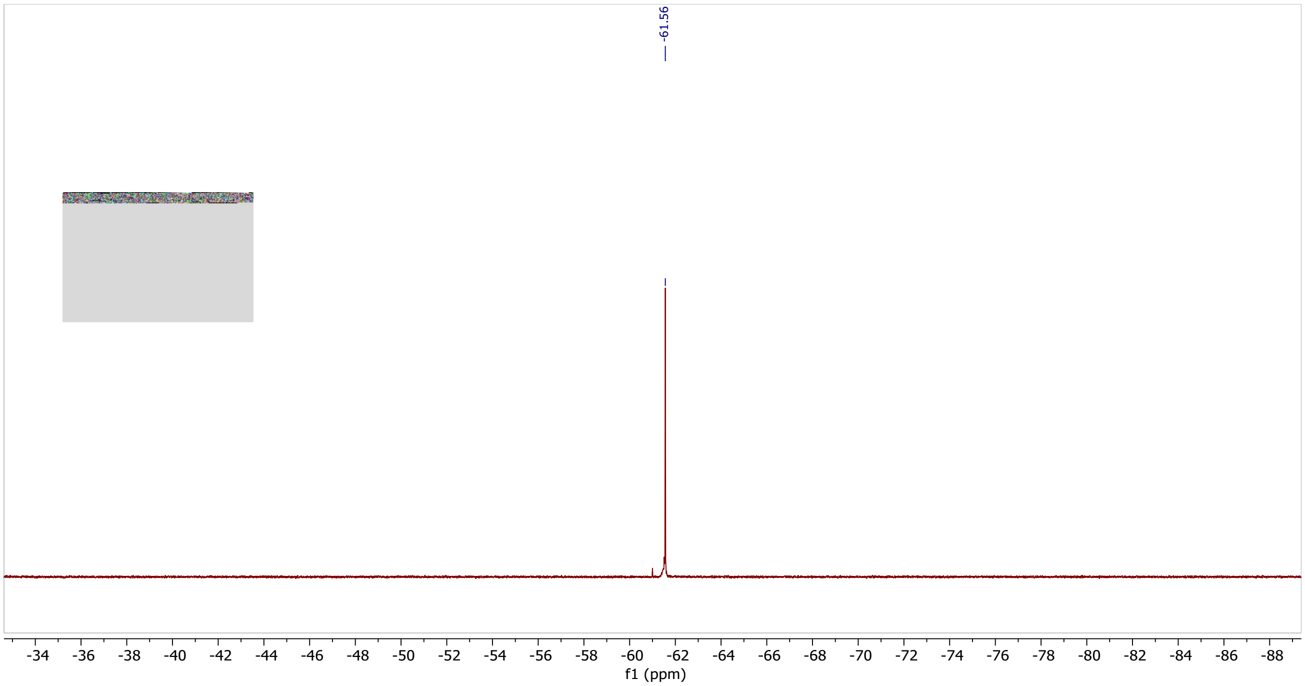

^{1}H NMR (500 MHz, $CDCl_3$) spectrum of **3aa**

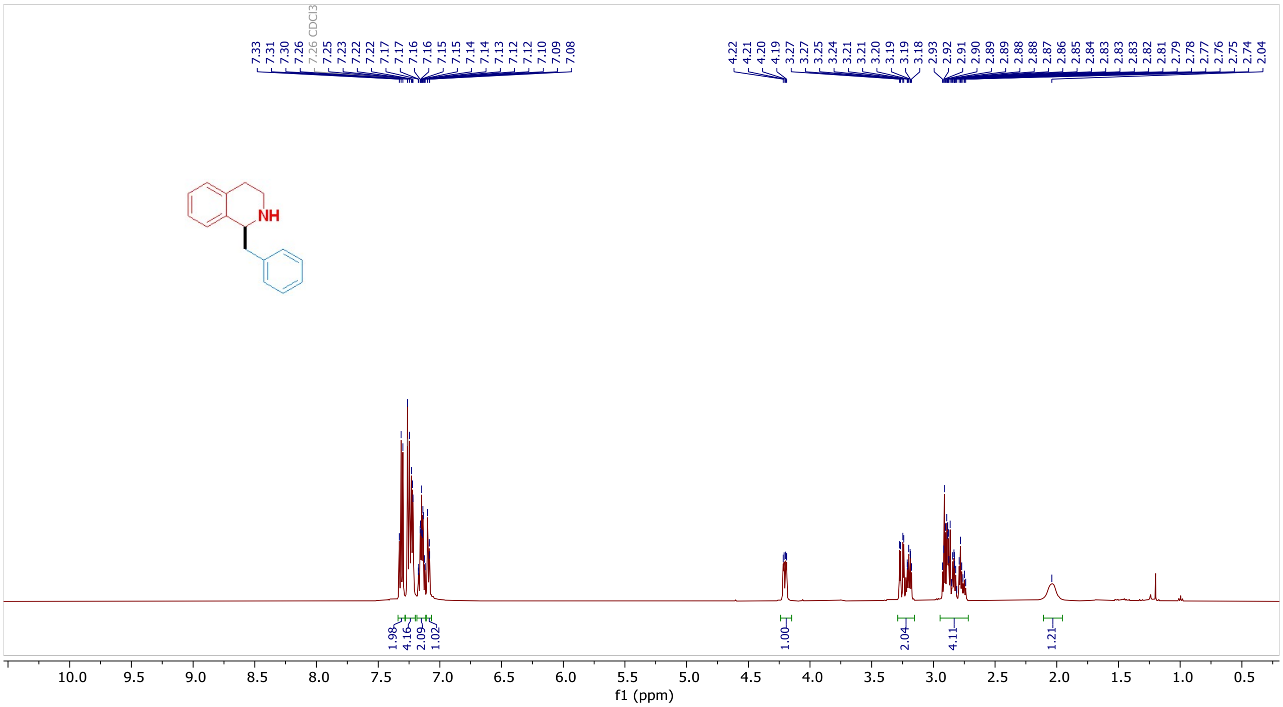

$^{13}C\{^{1}H\}$ NMR (126 MHz, $CDCl_3$) spectrum of **3aa**

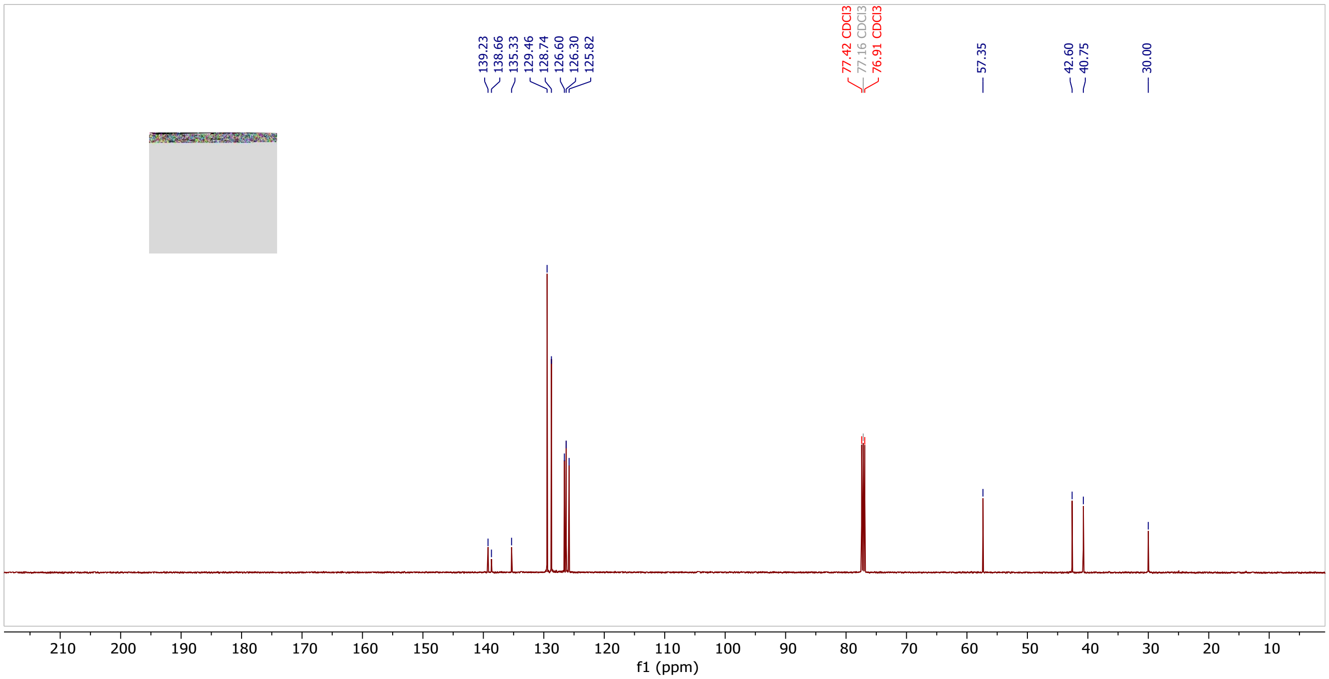

^{1}H NMR (500 MHz, $CDCl_3$) spectrum of **3ab**

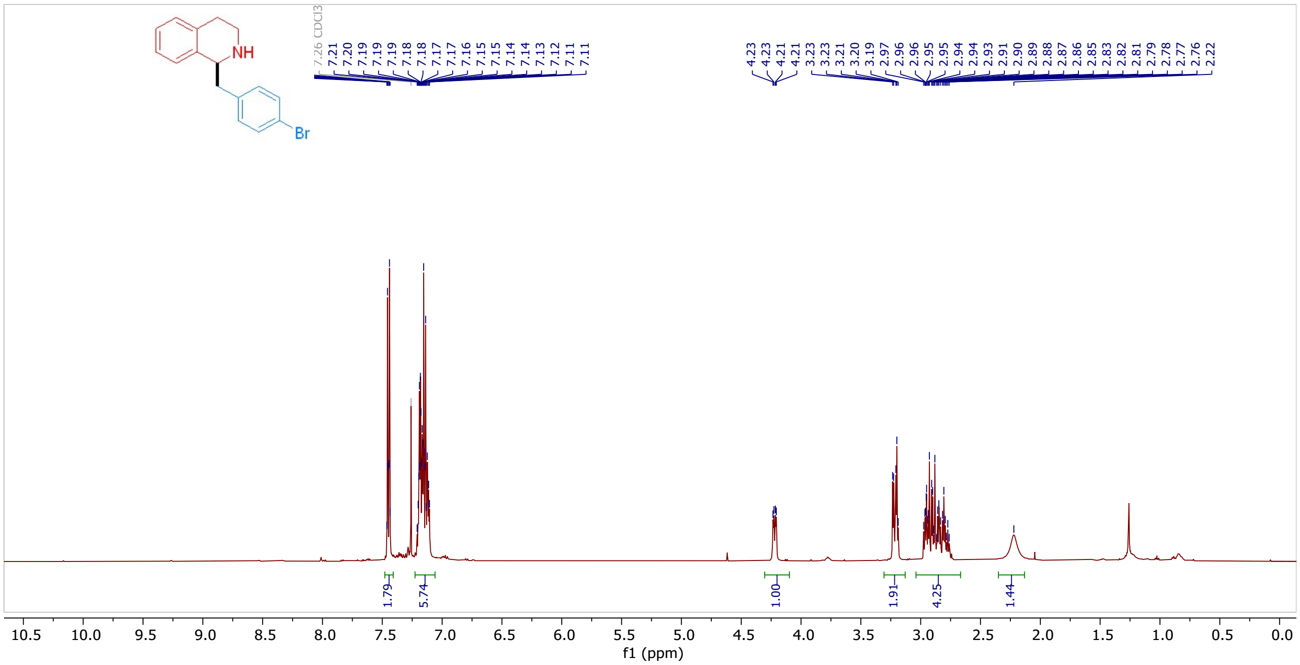

$^{13}C\{^{1}H\}$ NMR (126 MHz, $CDCl_3$) spectrum of **3ab**

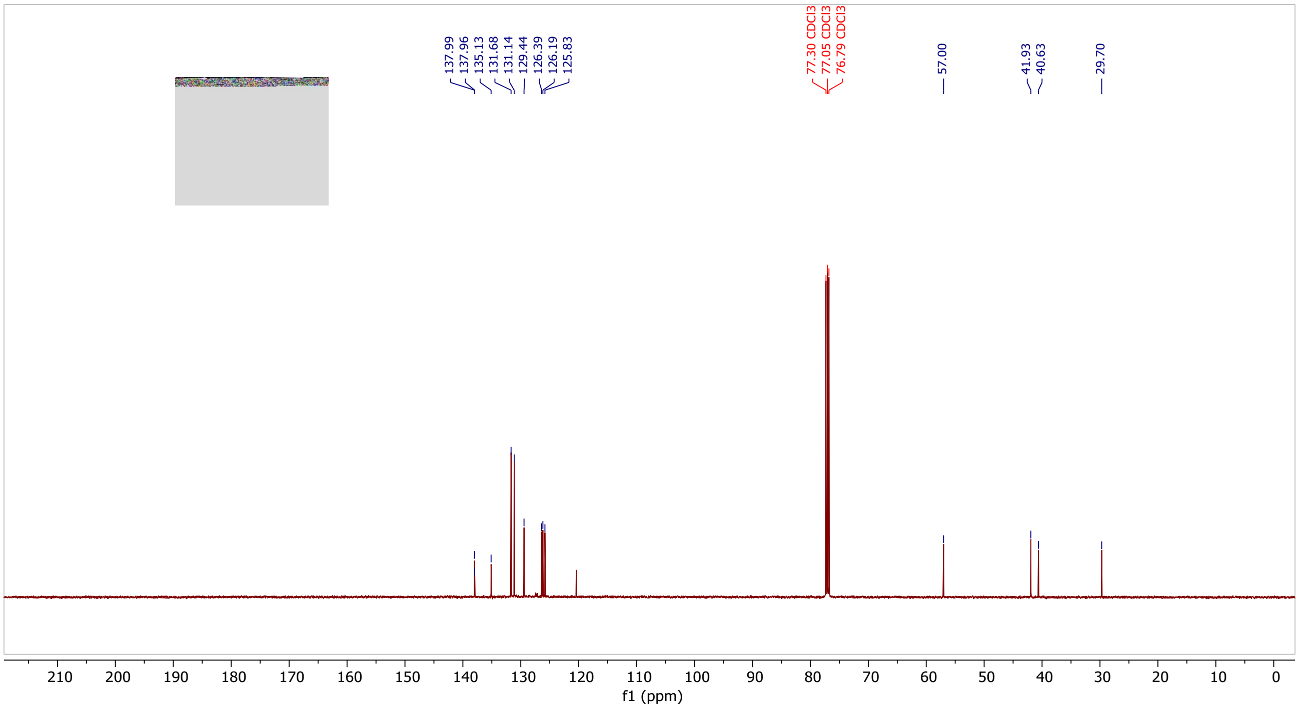

^{1}H NMR (500 MHz, $CDCl_3$) spectrum of **3ac**

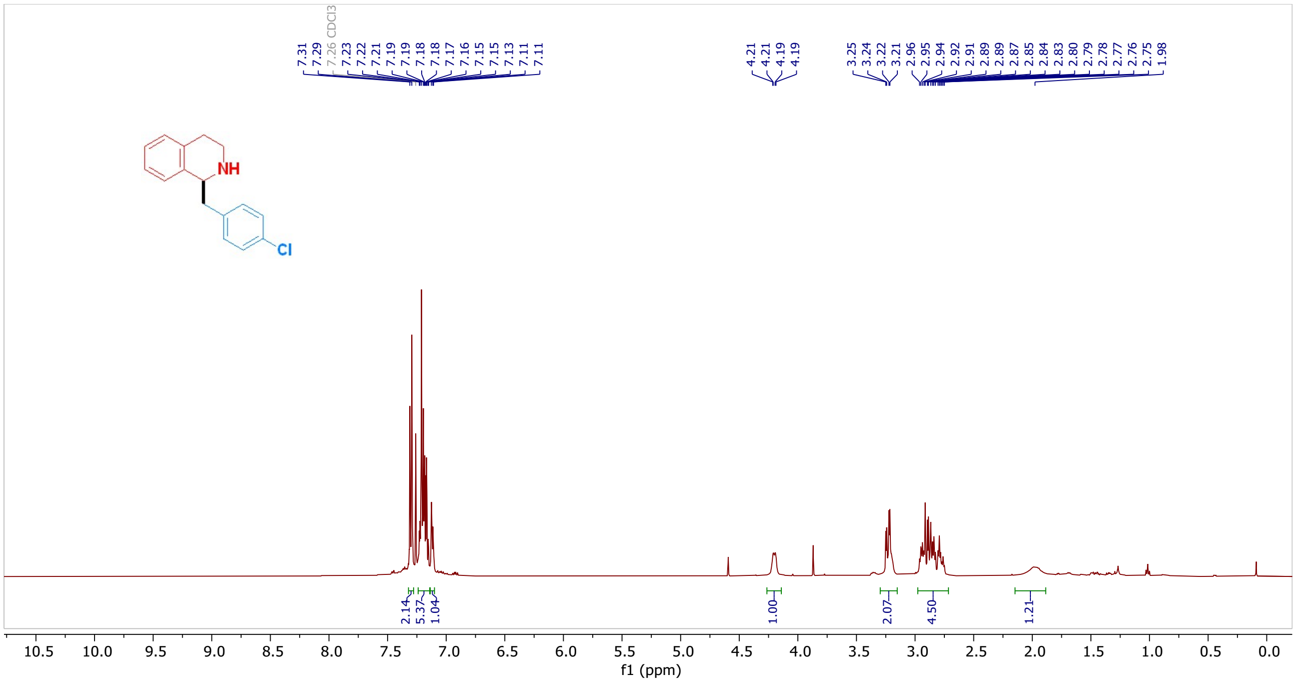

$^{13}C\{^{1}H\}$ NMR (126 MHz, $CDCl_3$) spectrum of **3ac**

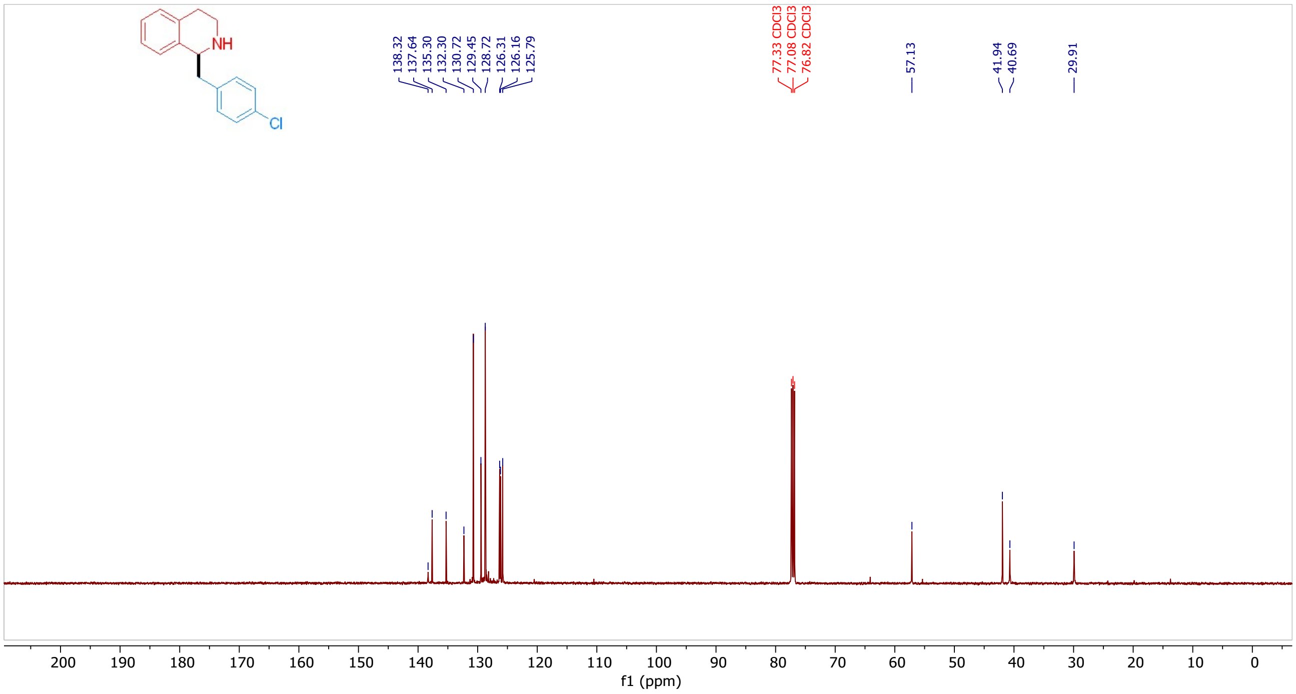

^{1}H NMR (500 MHz, $CDCl_3$) spectrum of **3ad**

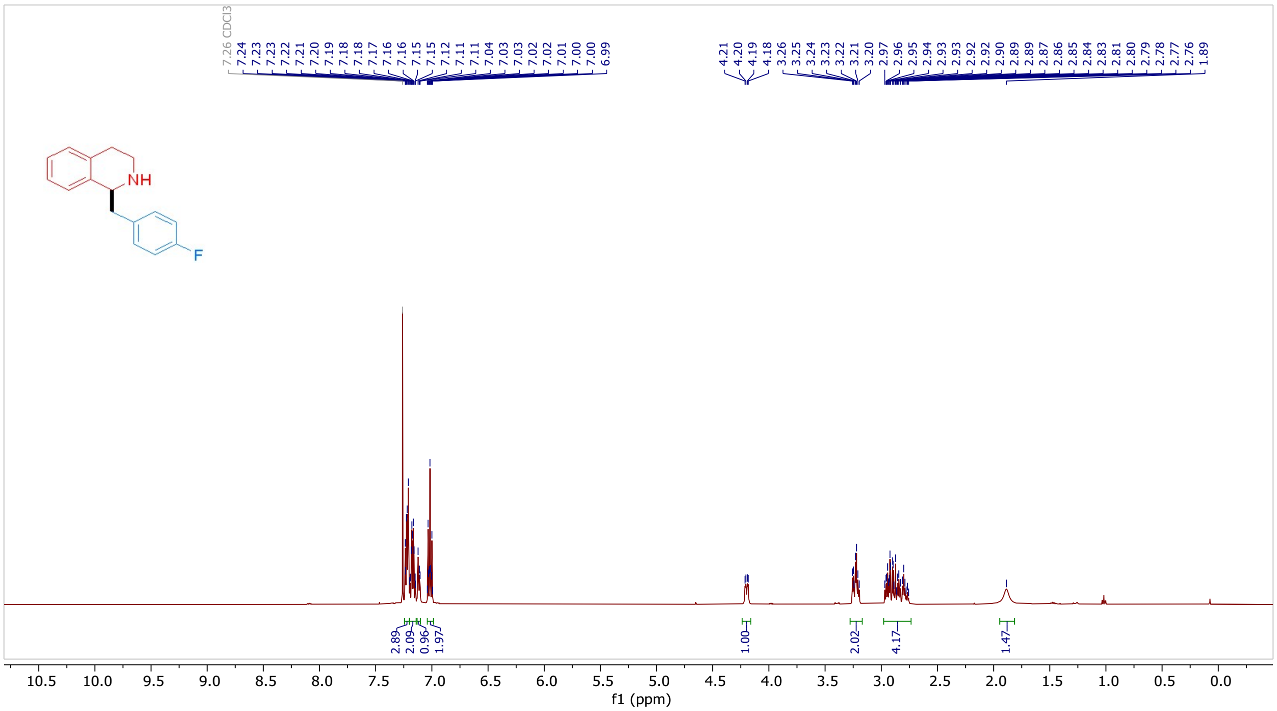

^{13}C{^{1}H} NMR (126 MHz, $CDCl_3$) spectrum of **3ad**

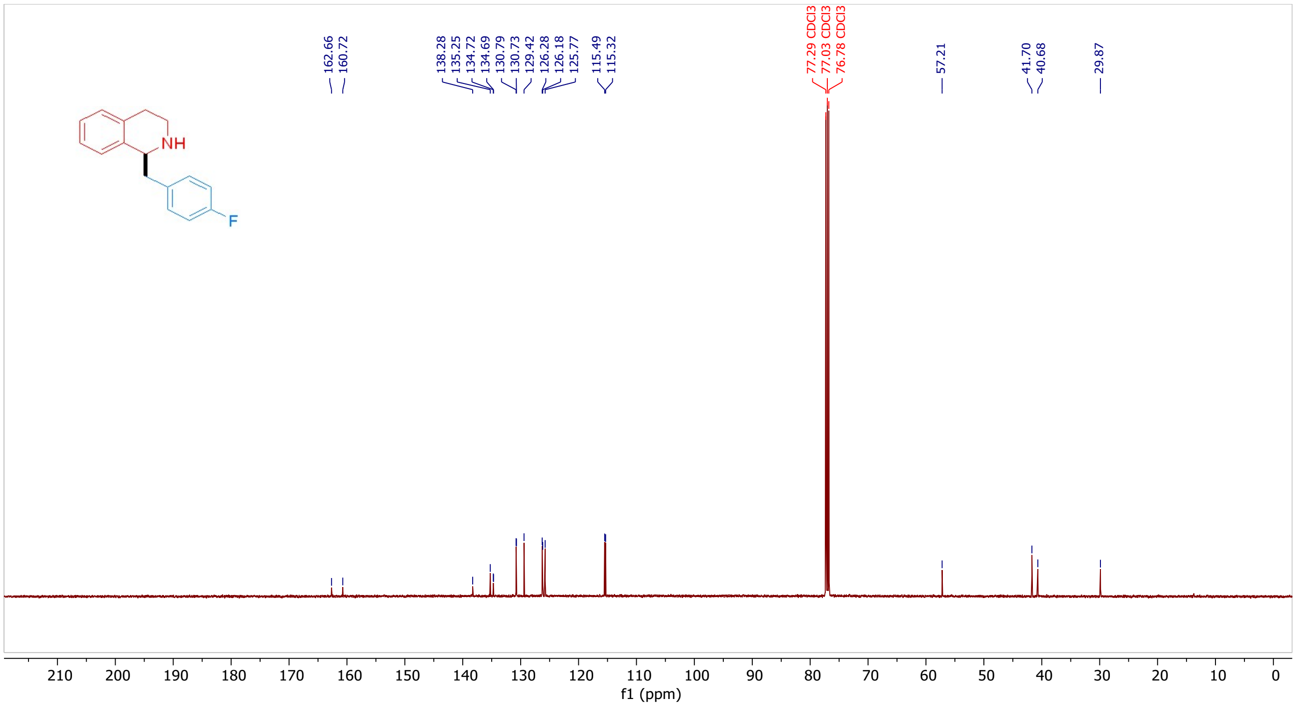

^{19}F NMR (471 MHz, CDCl$_3$) spectrum of **3ad**

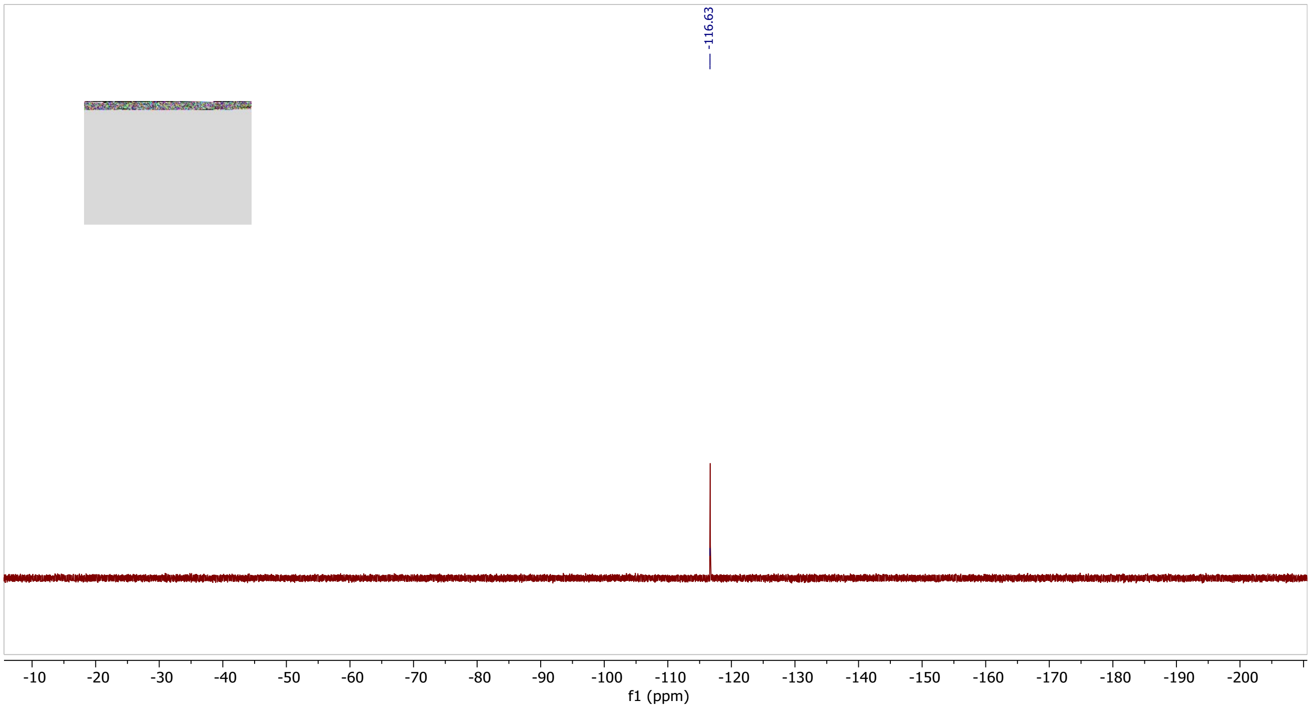

^{1}H NMR (500 MHz, CDCl$_3$) spectrum of **3ae**

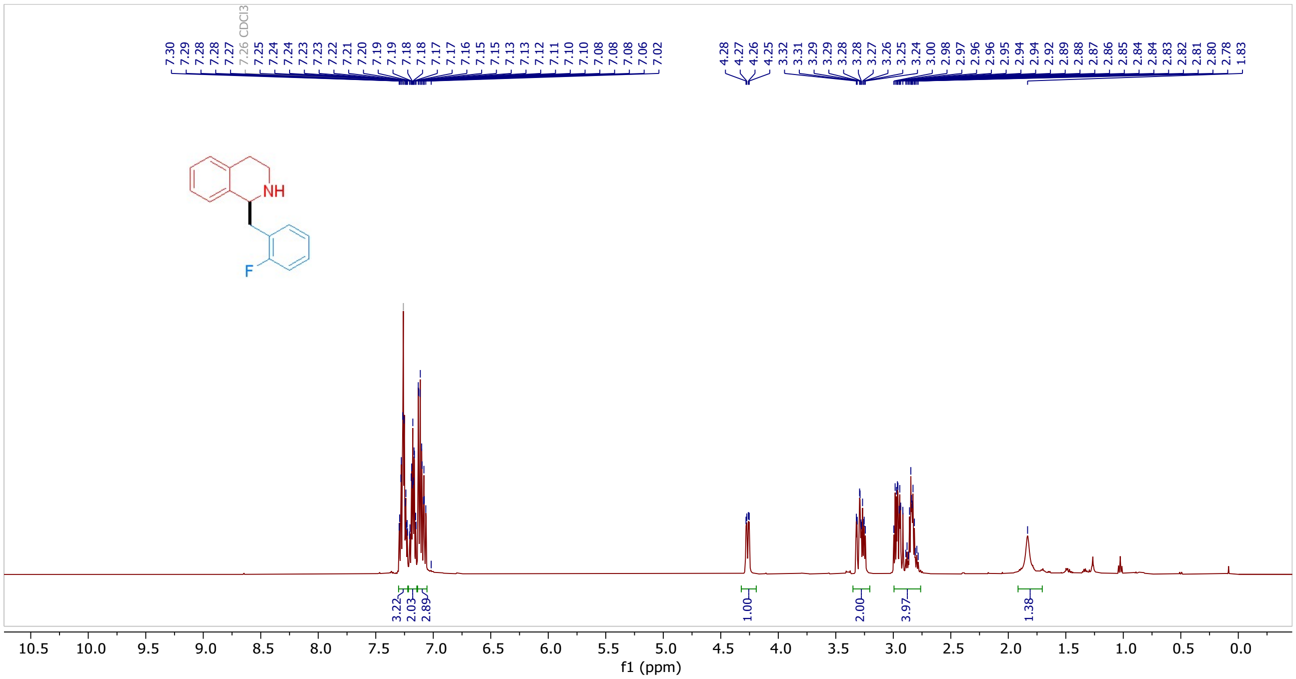

$^{13}C\{^1H\}$ NMR (126 MHz, $CDCl_3$) spectrum of **3ae**

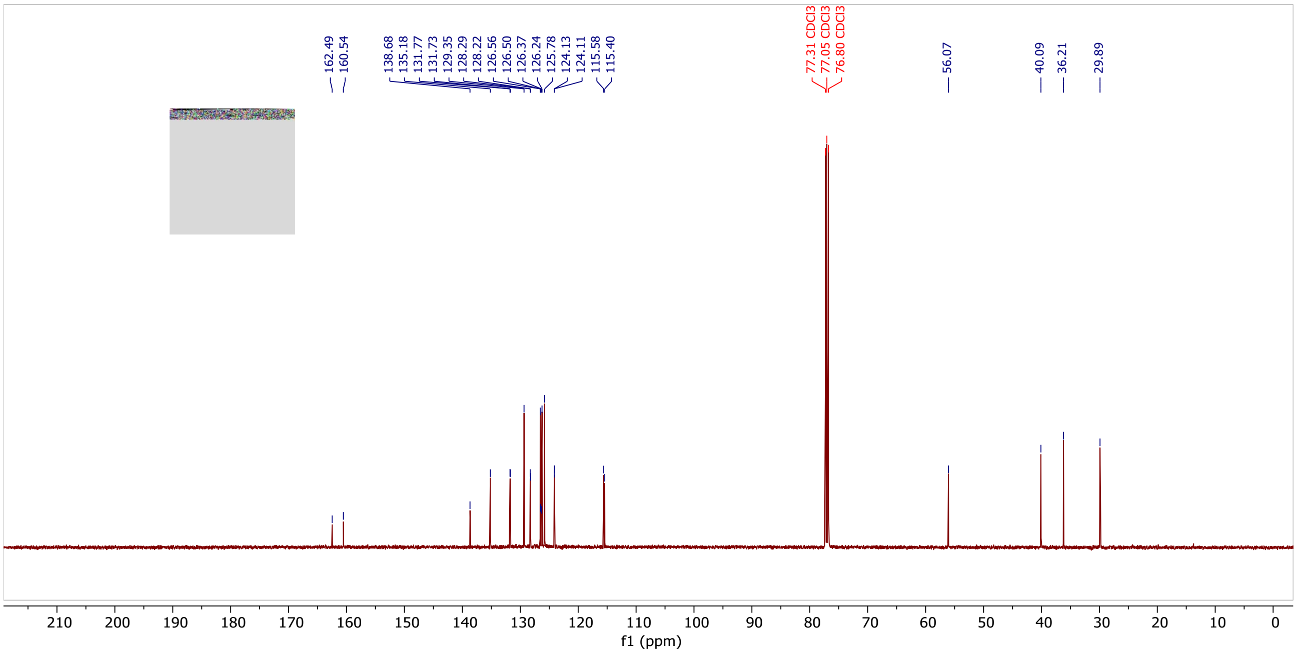

^{19}F NMR (471 MHz, $CDCl_3$) spectrum of **3ae**

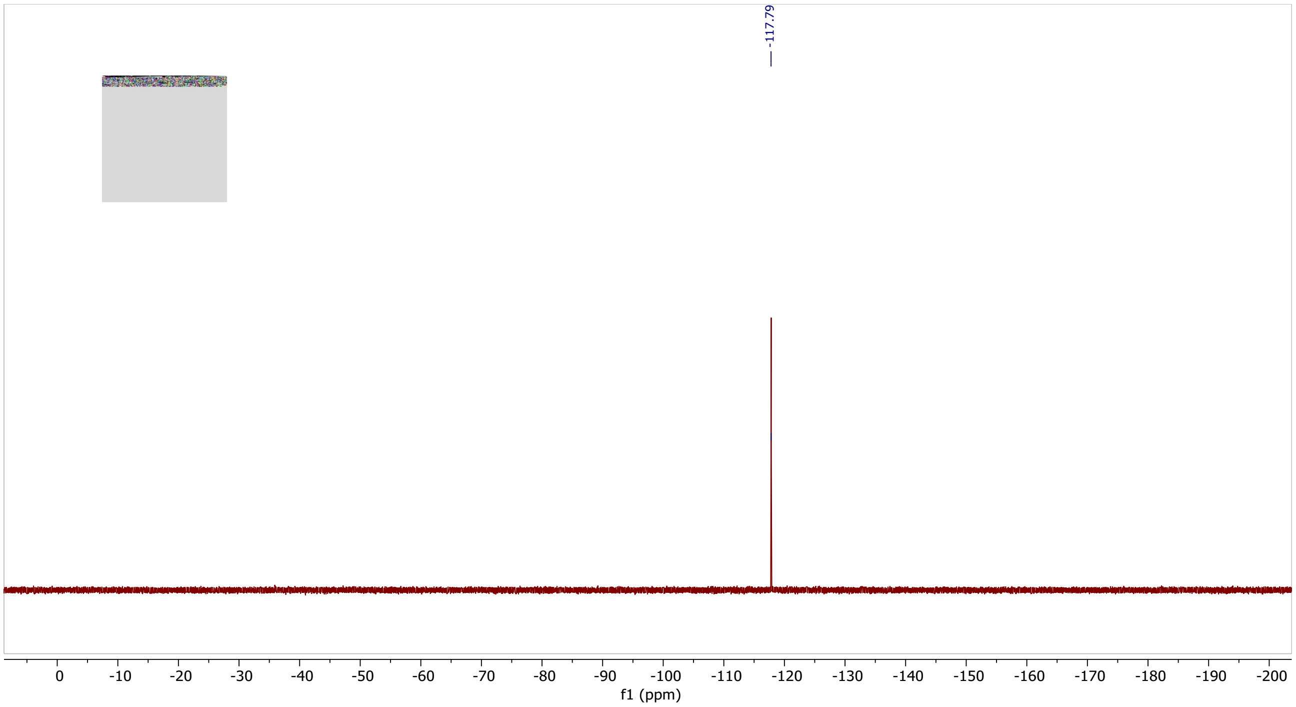

^1H NMR (500 MHz, $CDCl_3$) spectrum of **3af**

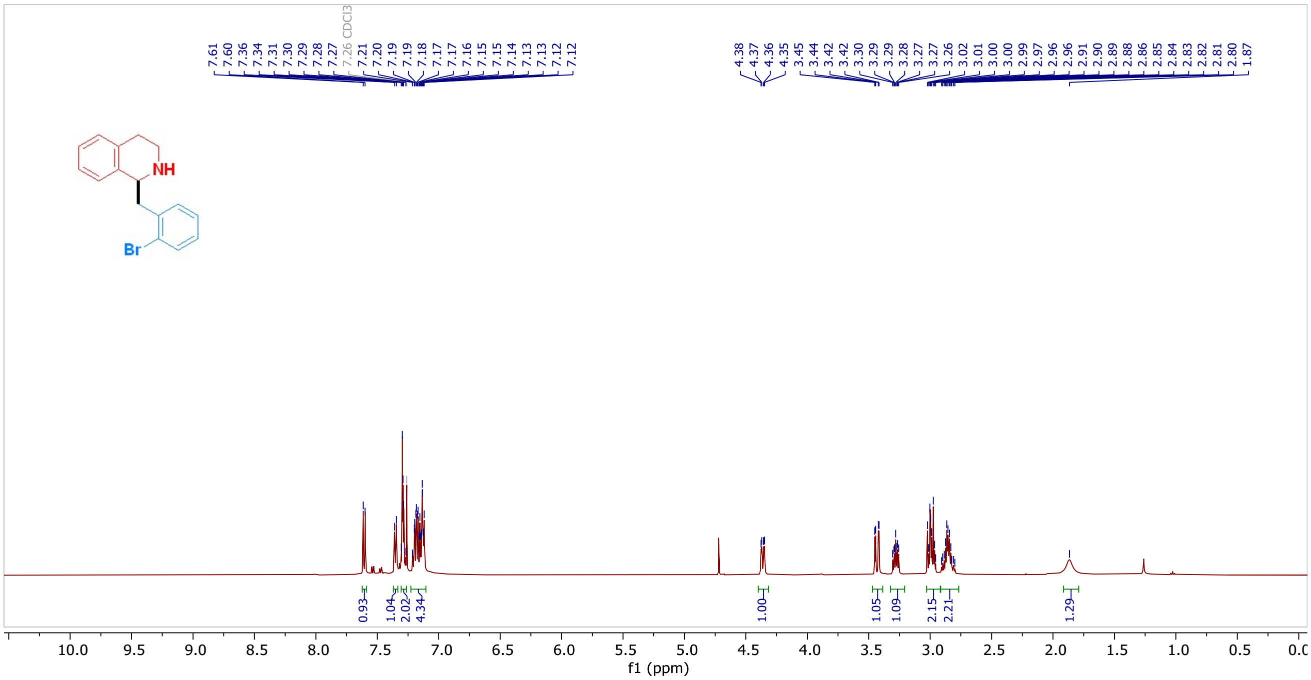

$^{13}C\{^1H\}$ NMR (126 MHz, $CDCl_3$) spectrum of **3af**

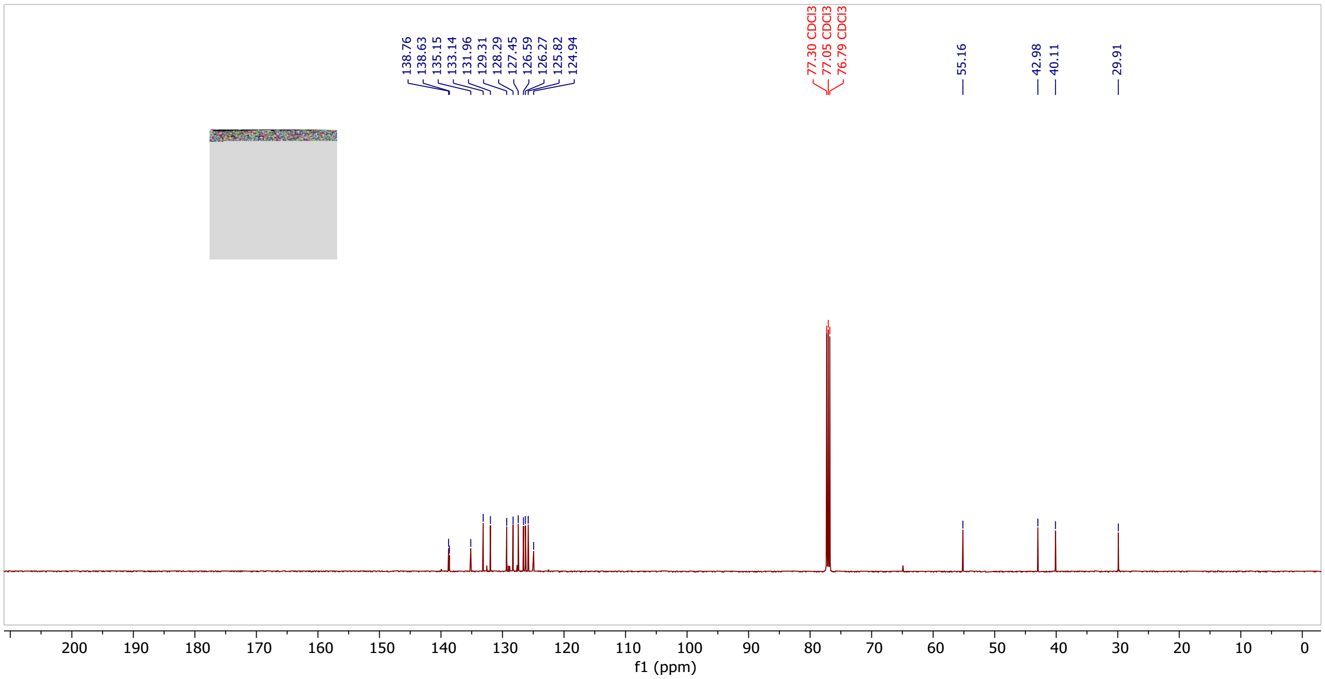

^1H NMR (500 MHz, $CDCl_3$) spectrum of **3ag**

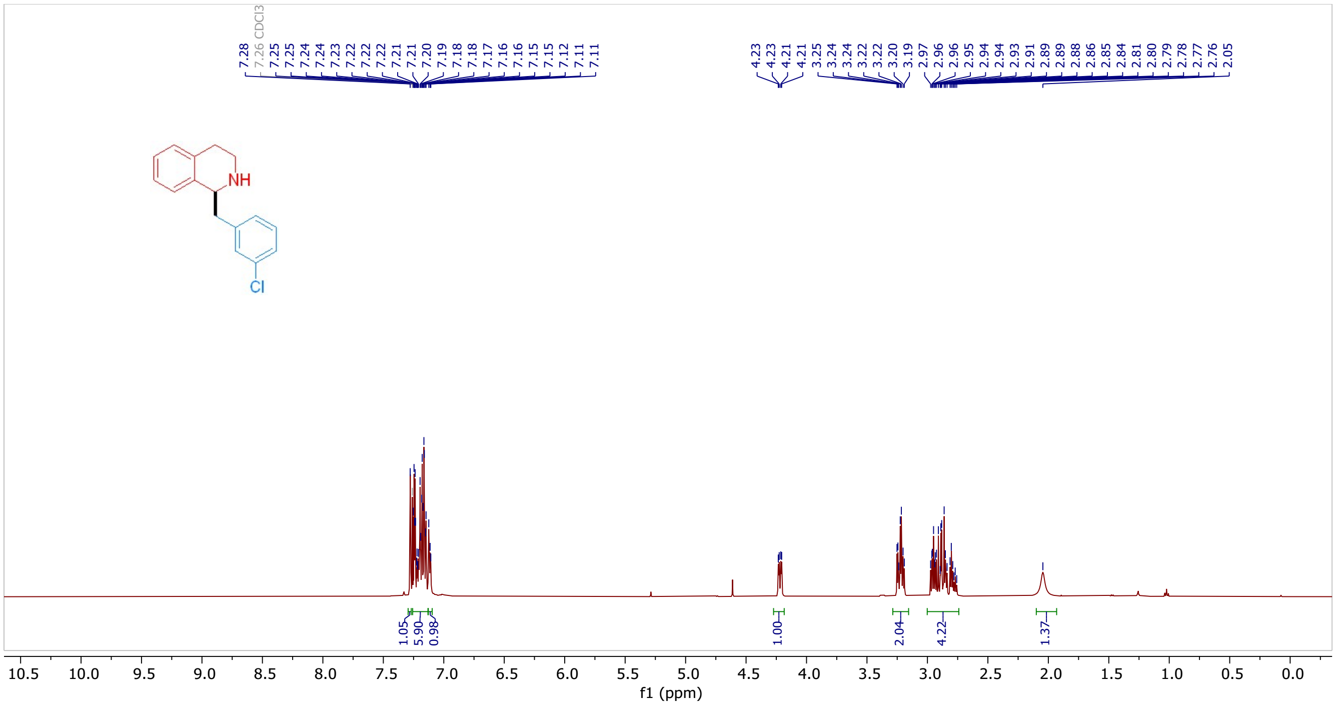

$^{13}C\{^{1}H\}$ NMR (126 MHz, $CDCl_3$) spectrum of **3ag**

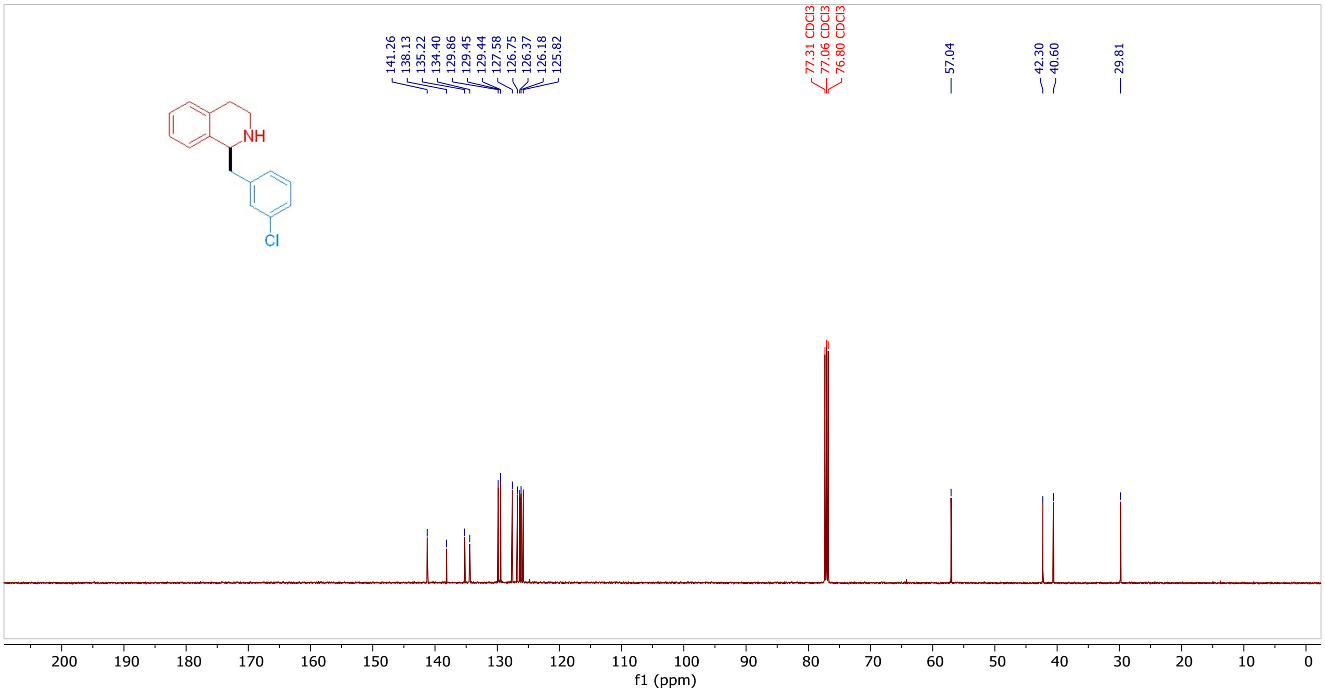

^{1}H NMR (500 MHz, $CDCl_3$) spectrum of **3ah**

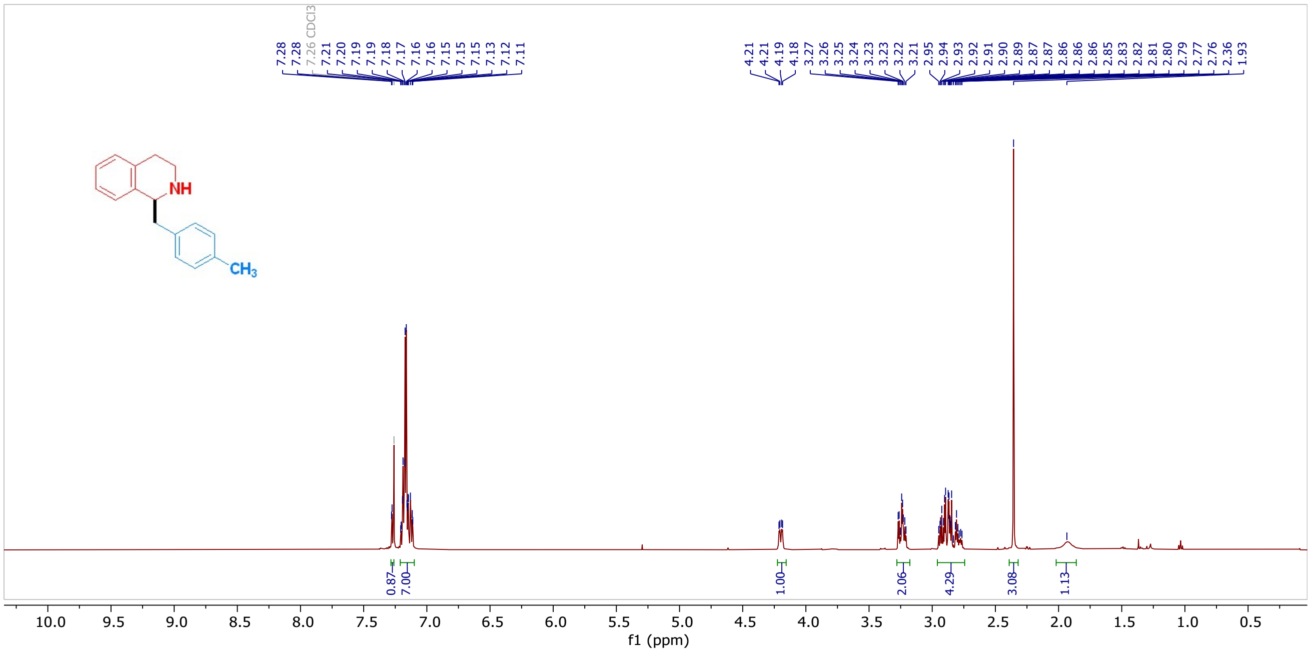

$^{13}C\{1H\}$ NMR (126 MHz, $CDCl_3$) spectrum of **3ah**

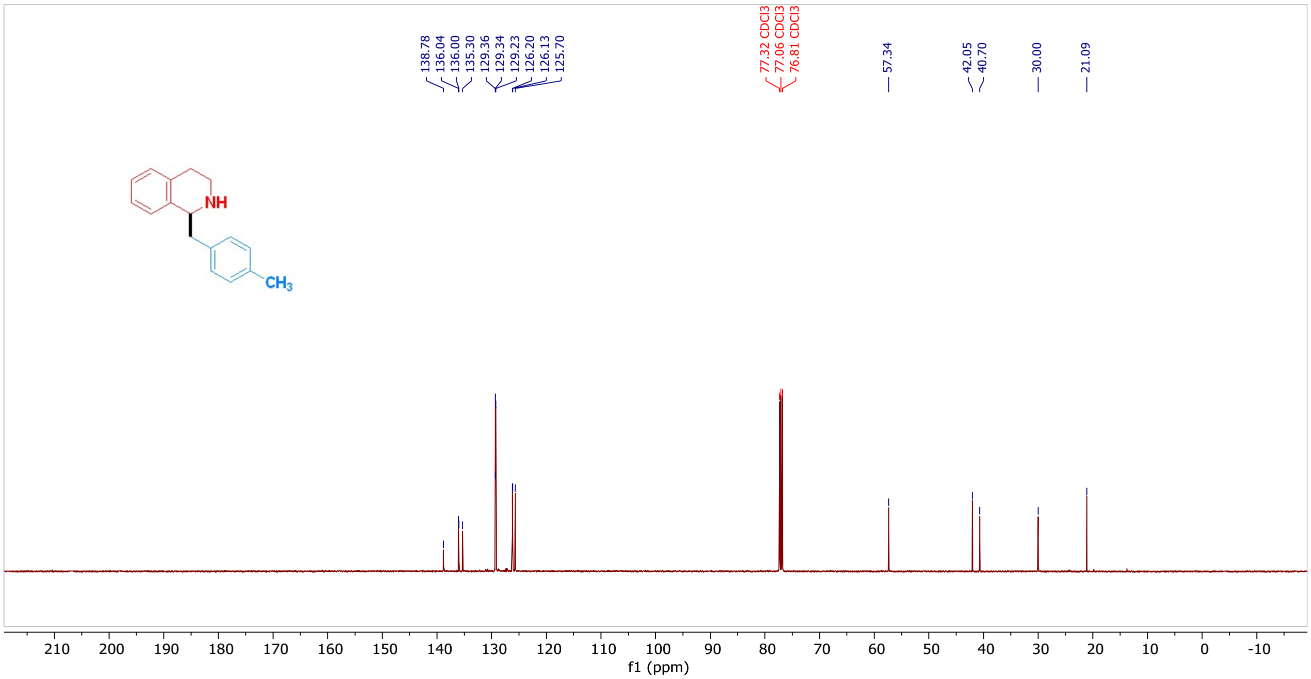

^{1}H NMR (500 MHz, $CDCl_3$) spectrum of **3ai**

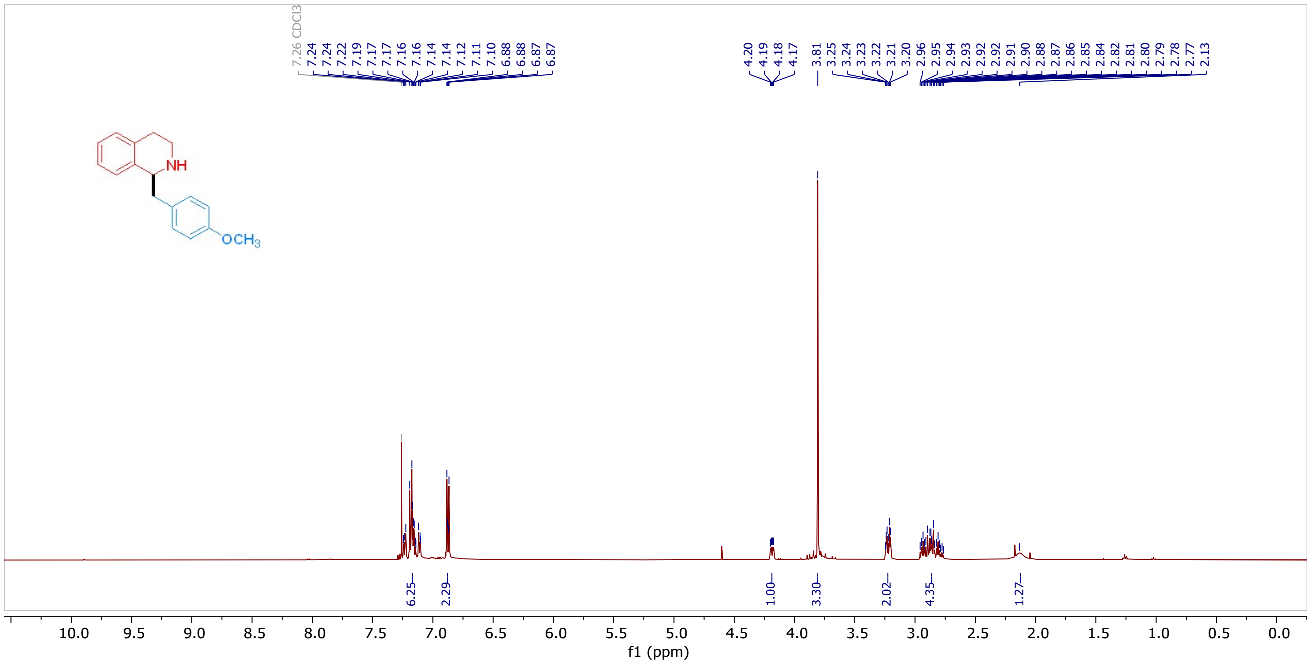

^{13}C{^{1}H} NMR (126 MHz, $CDCl_3$) spectrum of **3ai**

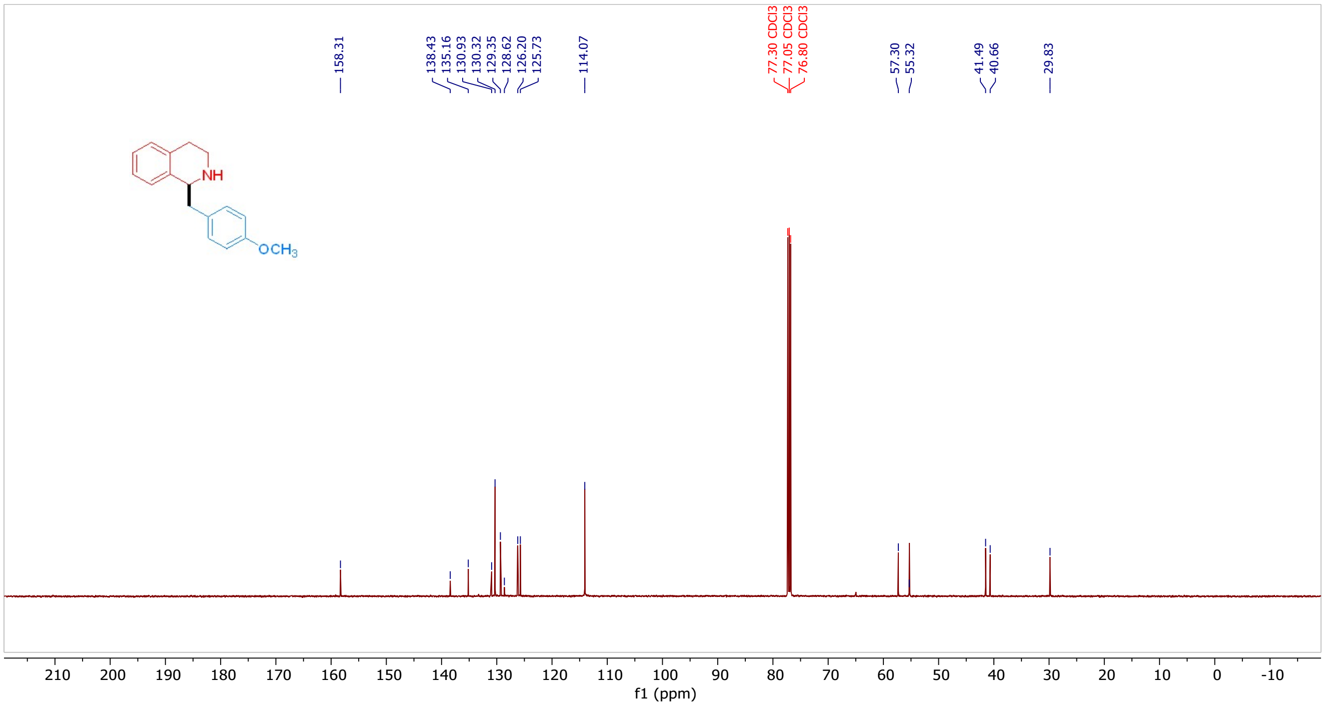

^{1}H NMR (500 MHz, $CDCl_3$) spectrum of **3aj**

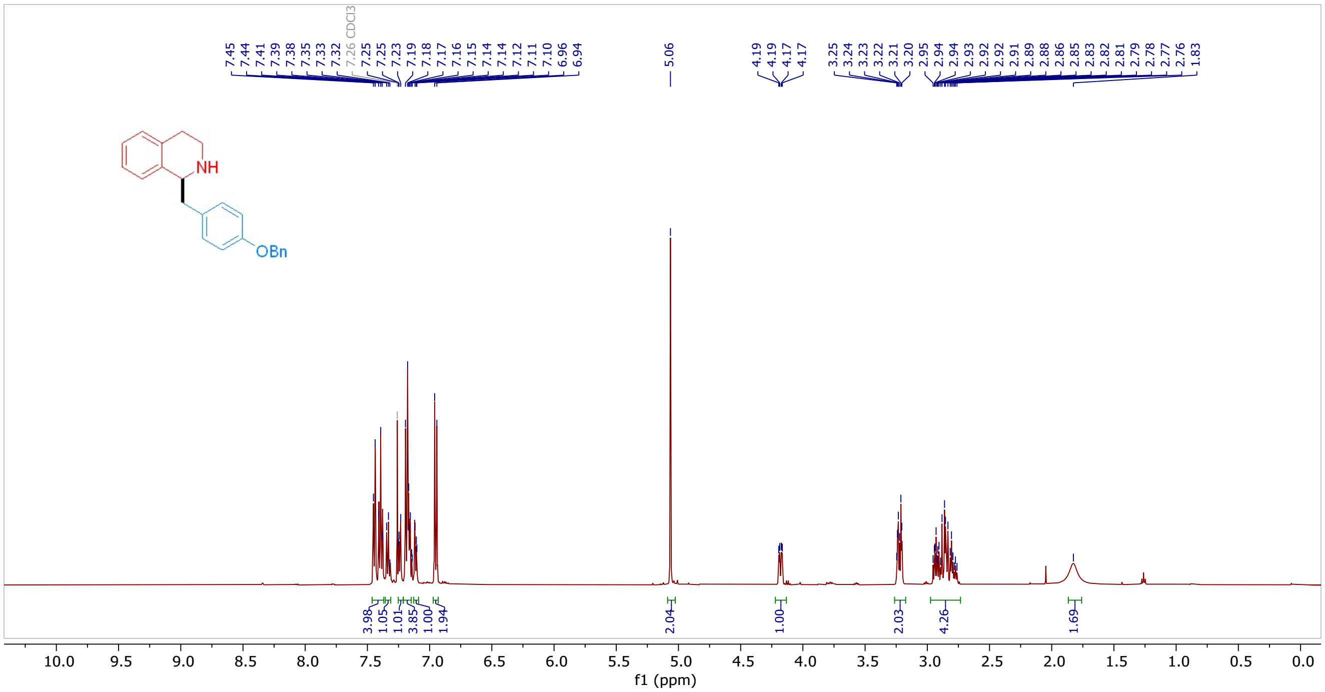

$^{13}C\{1H\}$ NMR (126 MHz, C spectrum of **3aj**

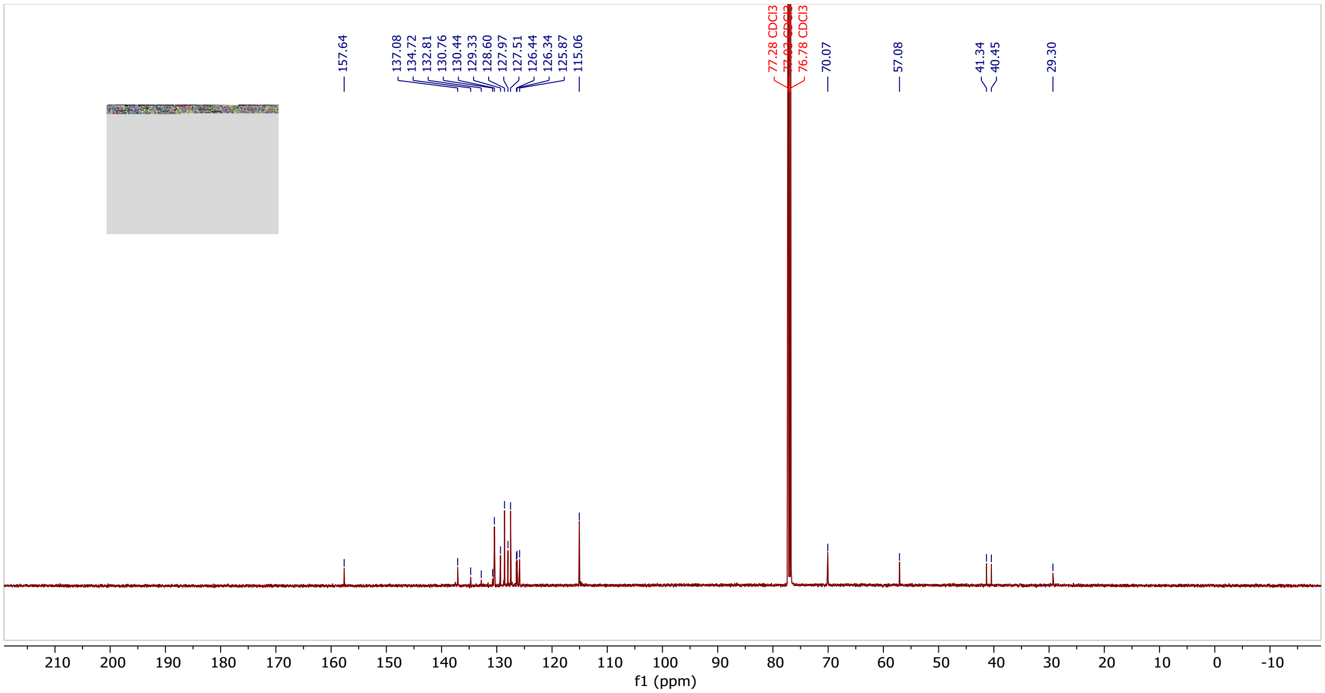

^{1}H NMR (500 MHz, $CDCl_3$) spectrum of **3ak**

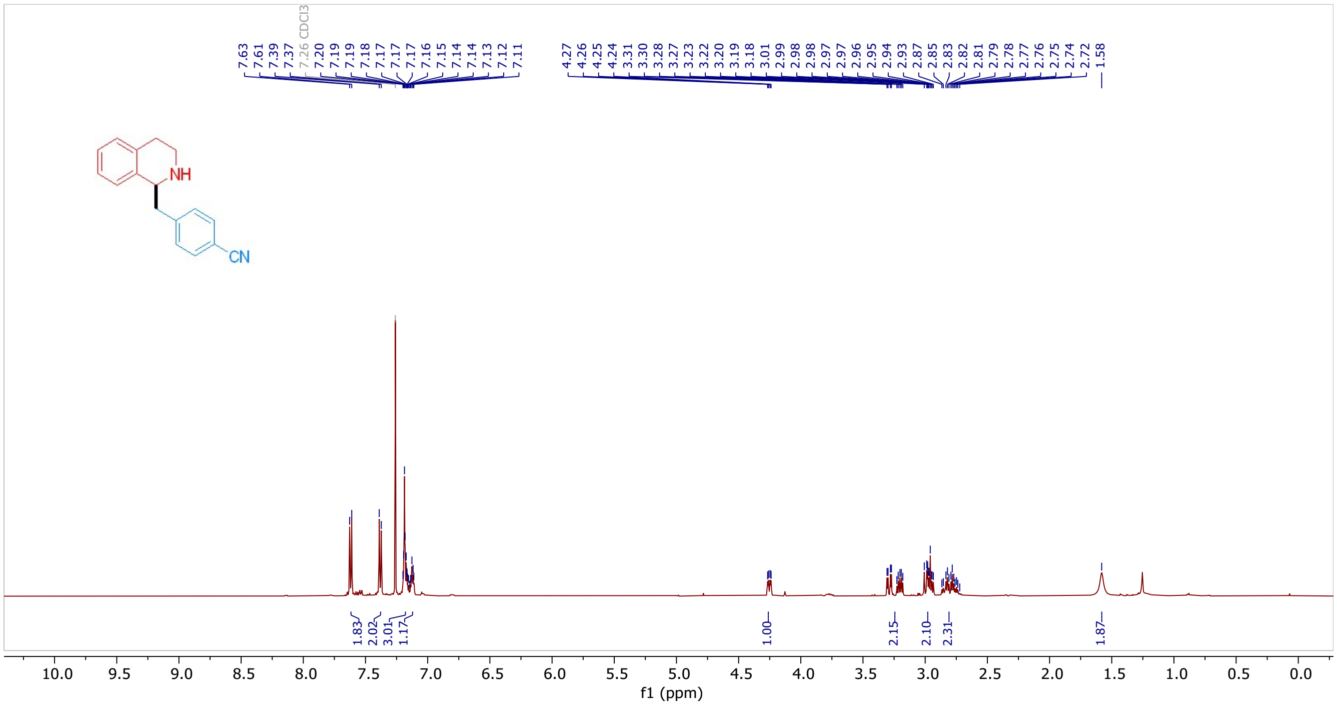

$^{13}C\{^{1}H\}$ NMR (126 MHz, $CDCl_3$) spectrum of **3ak**

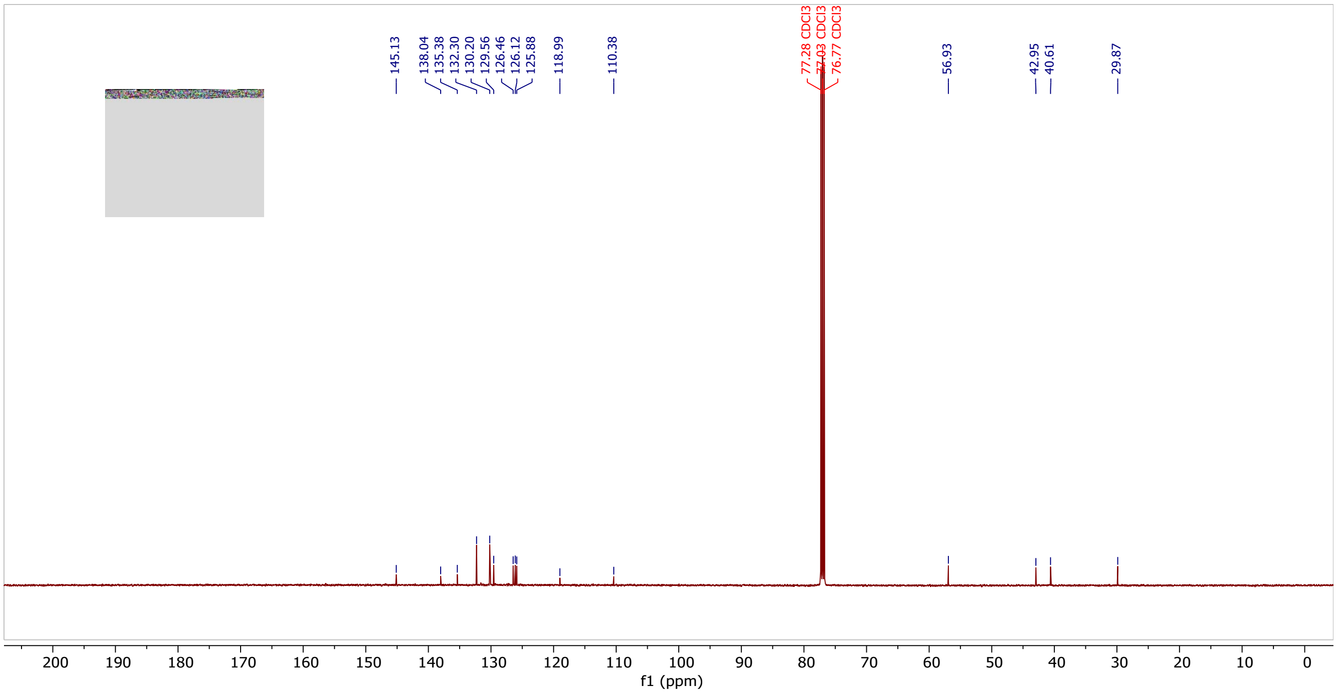

^{1}H NMR (500 MHz, $CDCl_3$) spectrum of **3al**

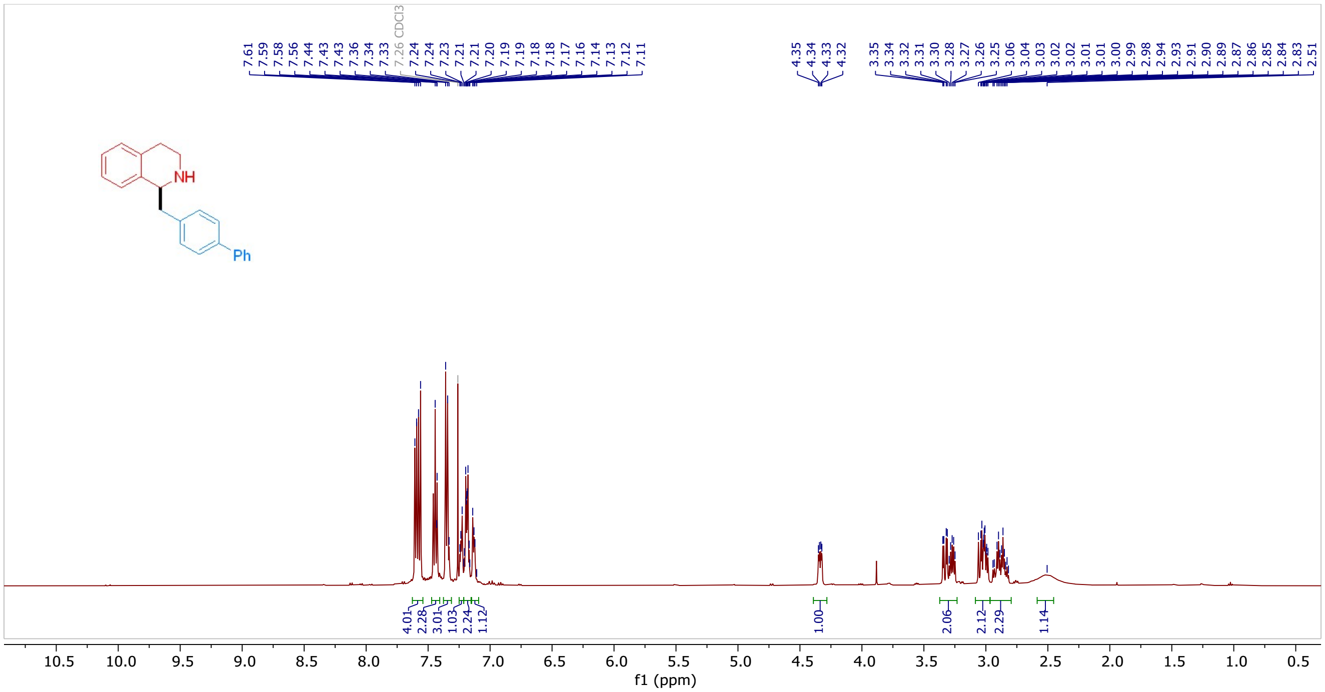

$^{13}C\{^{1}H\}$ NMR (126 MHz, $CDCl_3$) spectrum of **3al**

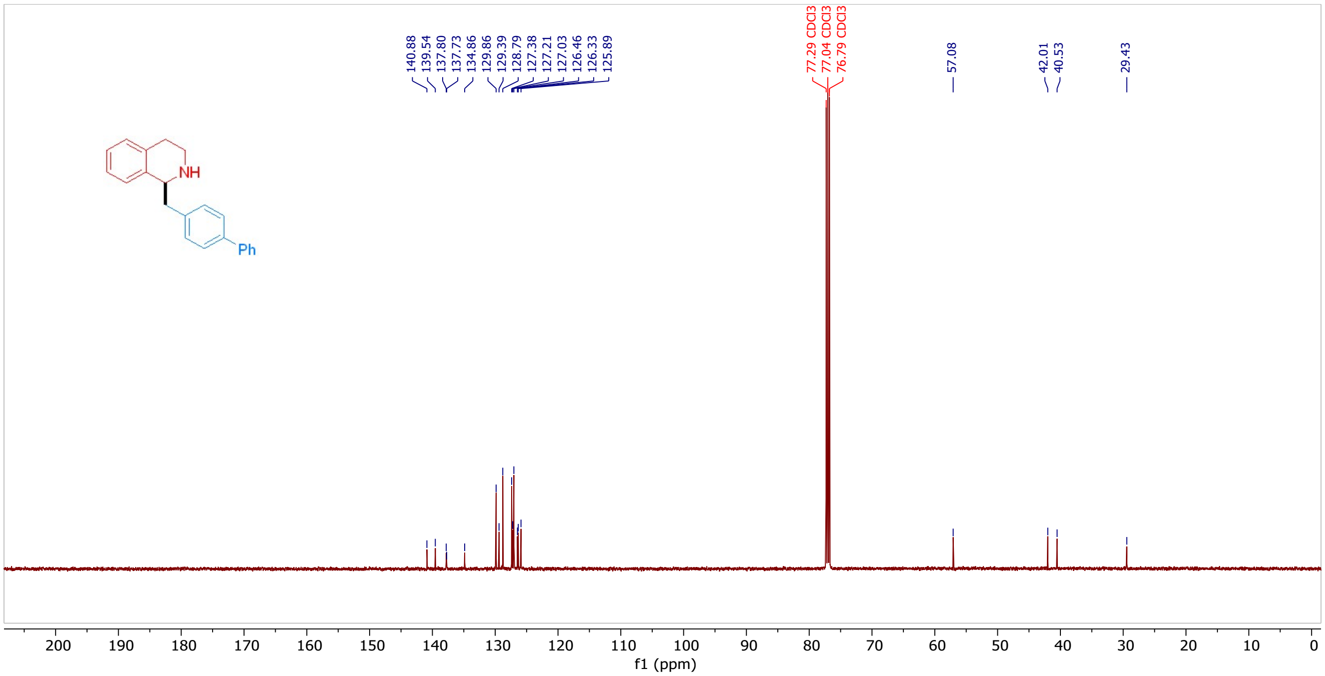

^{1}H NMR (500 M Hz, $CDCl_3$) spectrum of **3am**

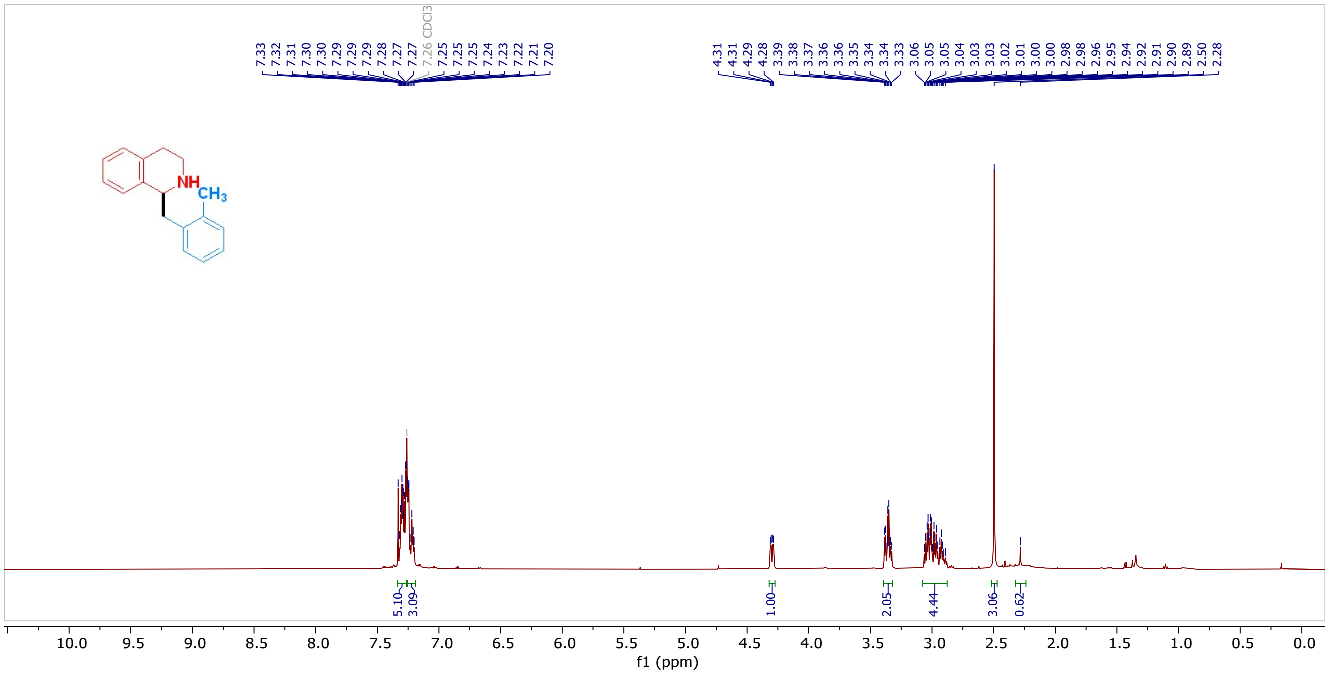

$^{13}C\{^{1}H\}$ NMR (126 MHz, $CDCl_3$) spectrum of **3am**

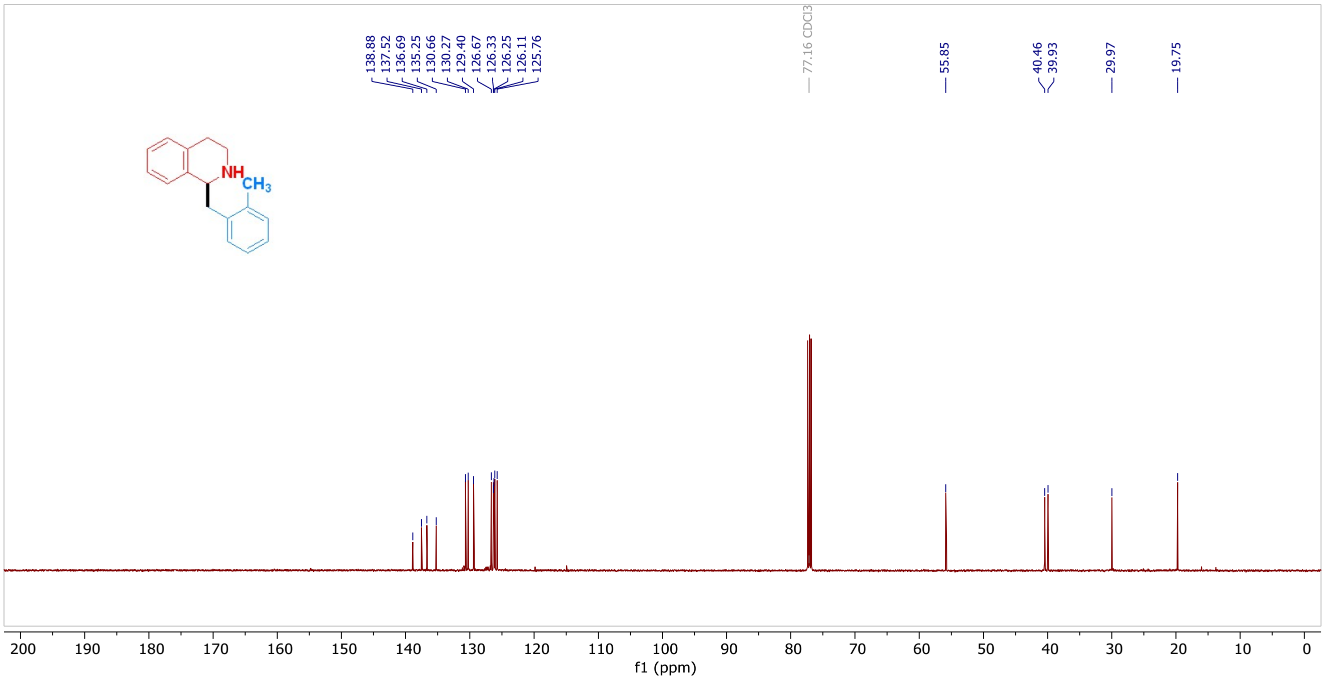

^{1}H NMR (500 MHz, $CDCl_3$) spectrum of **3an**

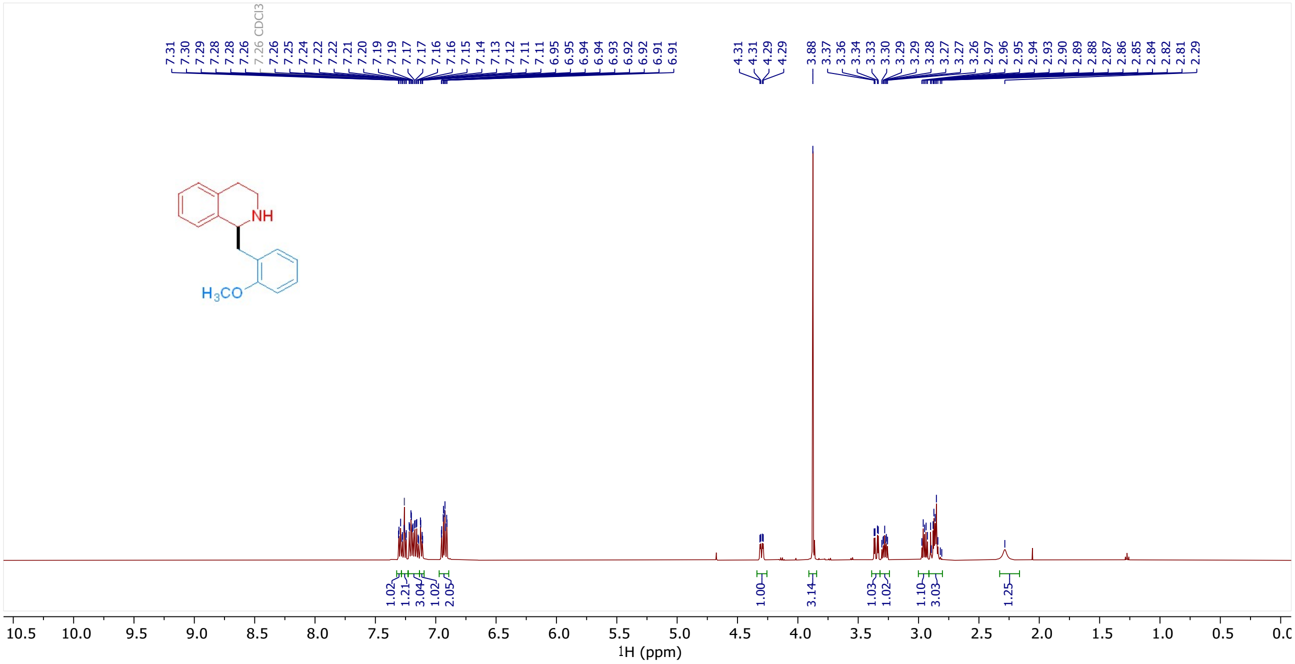

^{13}C{^{1}H} NMR (126 MHz, $CDCl_3$) spectrum of **3an**

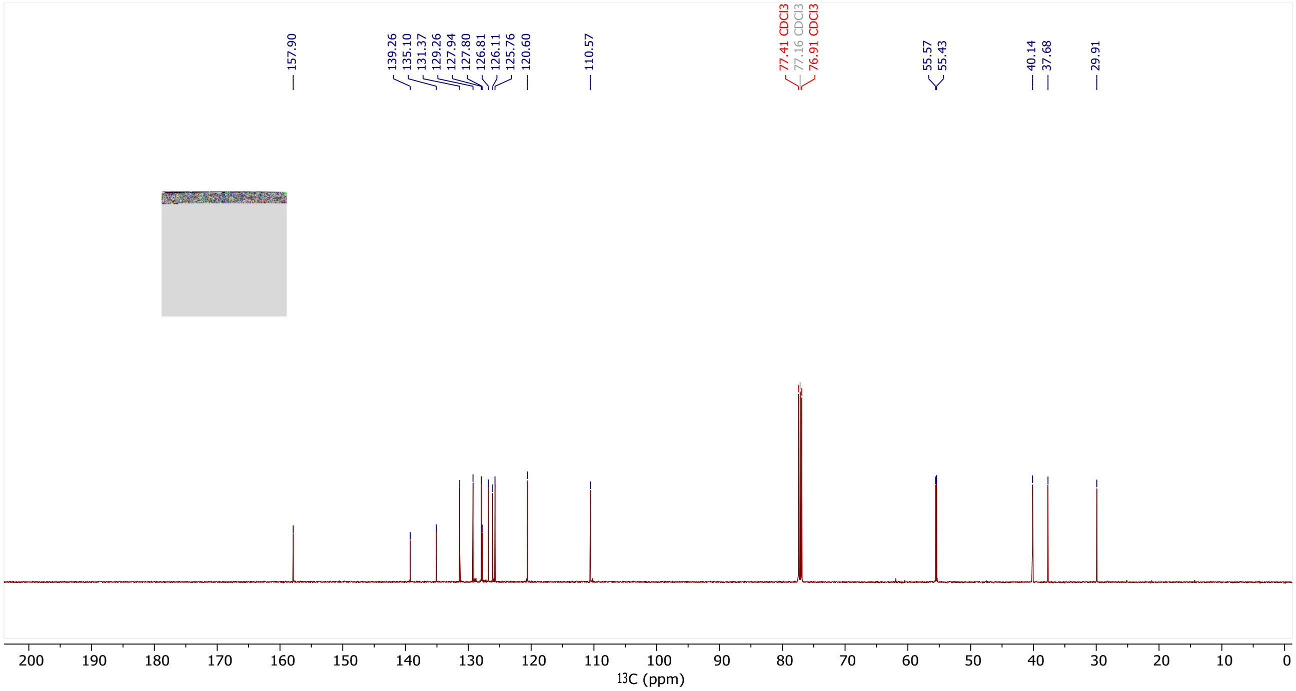

^{1}H NMR (500 MHz, $CDCl_3$) spectrum of **3ao**

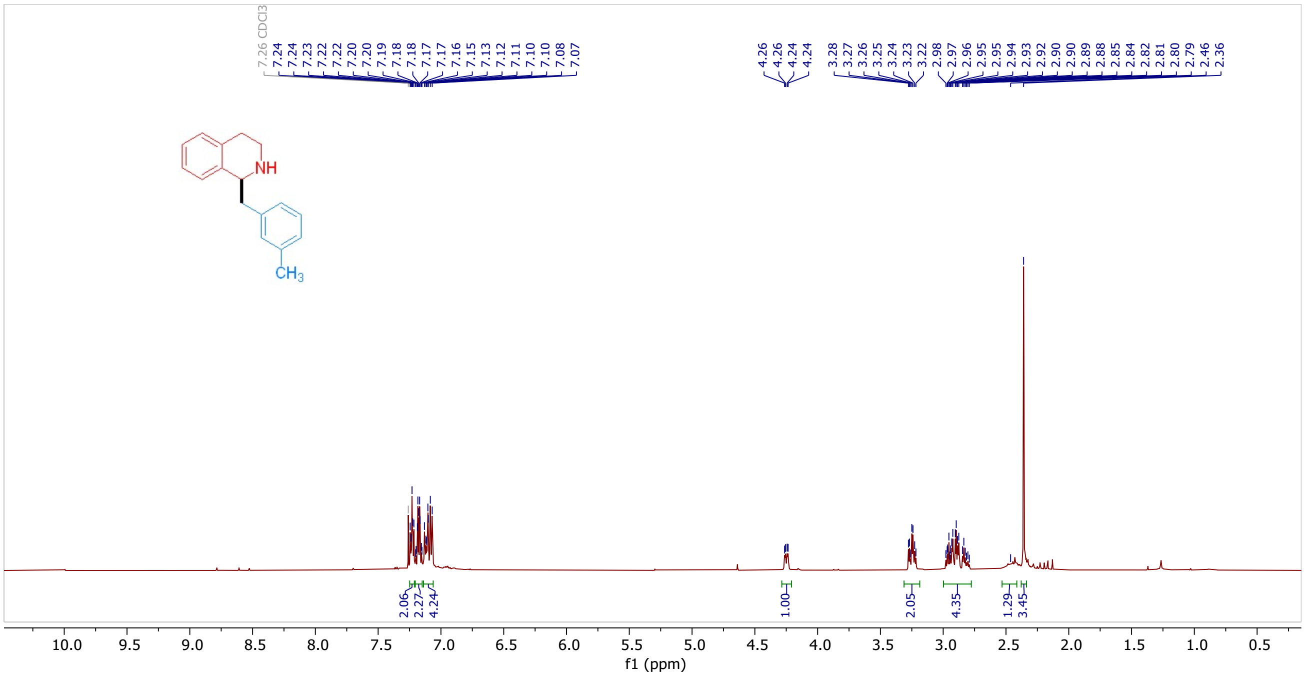

$^{13}C\{^1H\}$ NMR (126 MHz, $CDCl_3$) spectrum of **3ao**

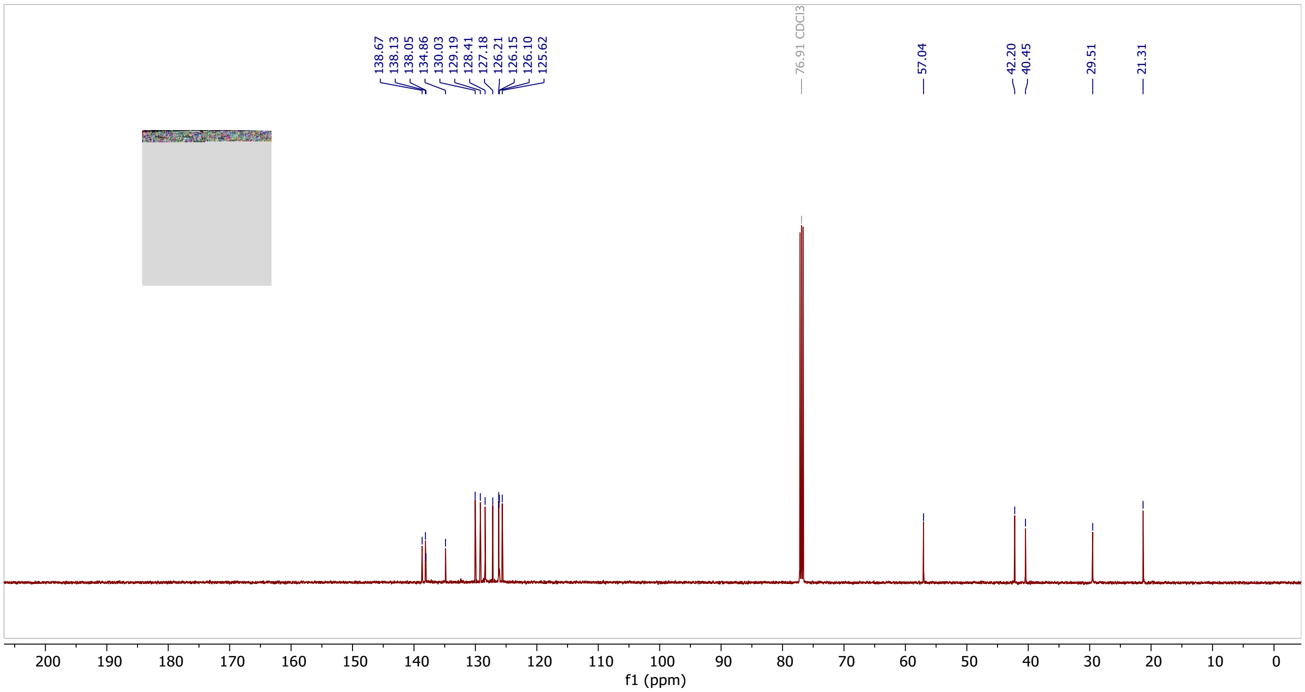

^1H NMR (500 MHz, $CDCl_3$) spectrum of **3ap**

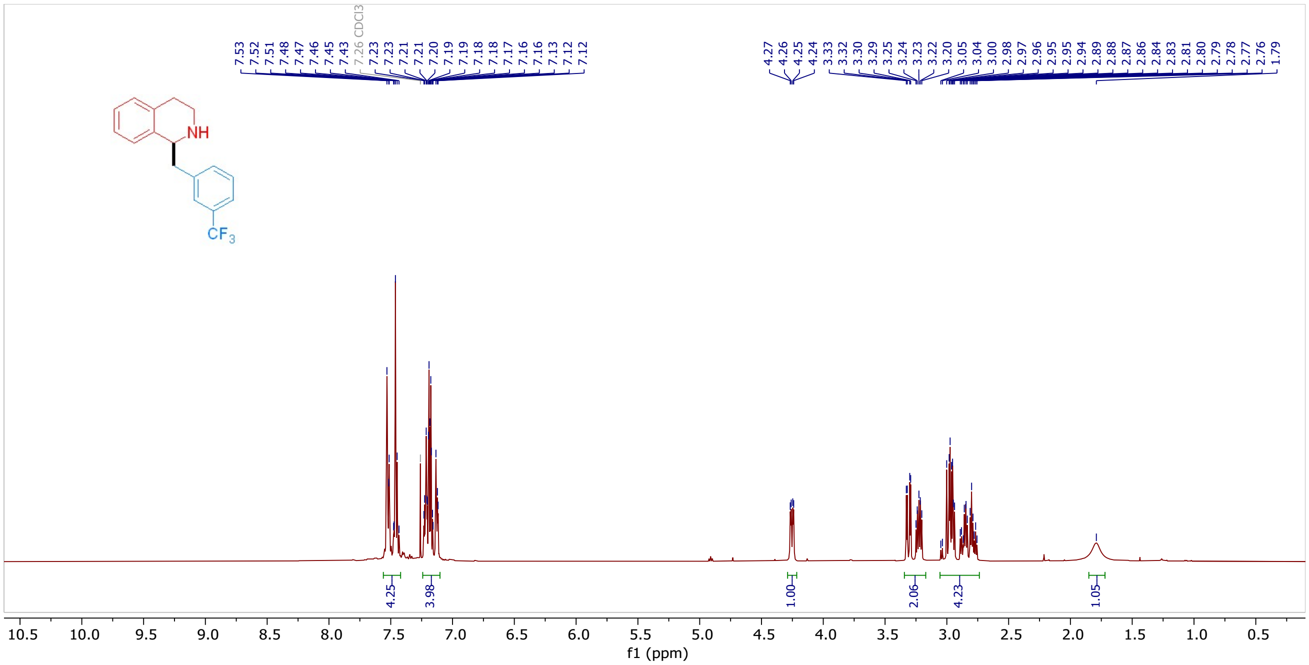

$^{13}C\{^{1}H\}$ NMR (126 MHz, $CDCl_3$) spectrum of **3ap**

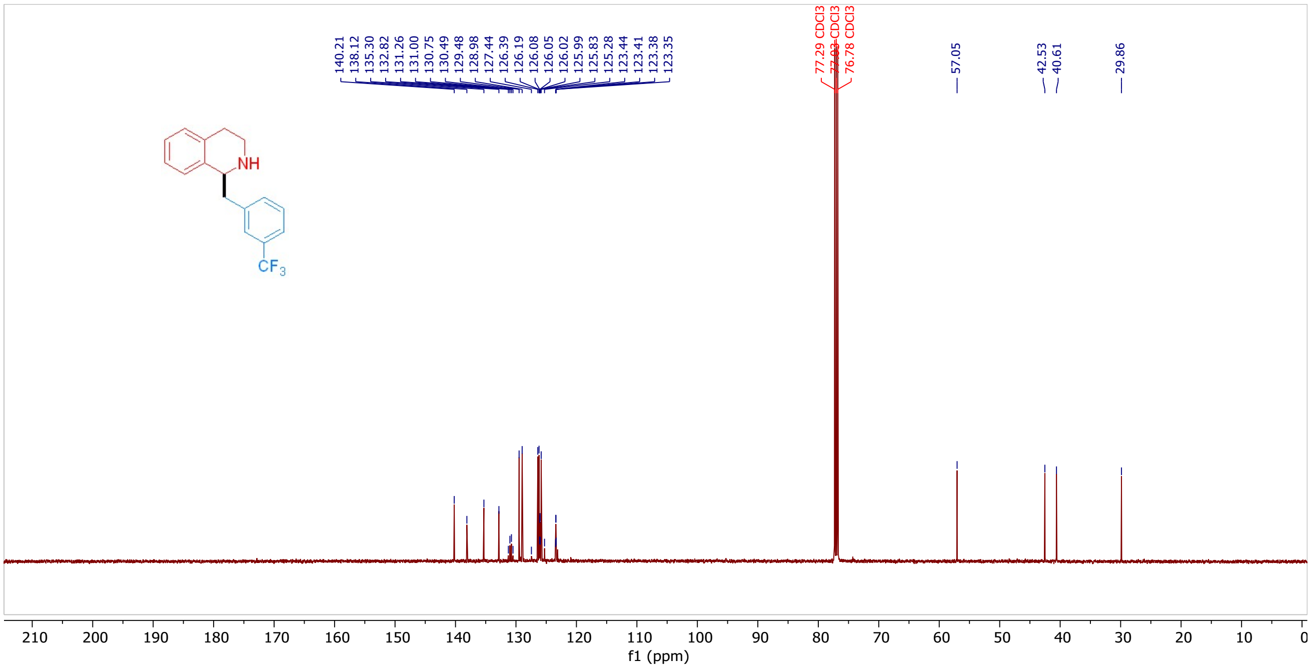

^{19}F NMR (471 MHz, $CDCl_3$) spectrum of **3ap**

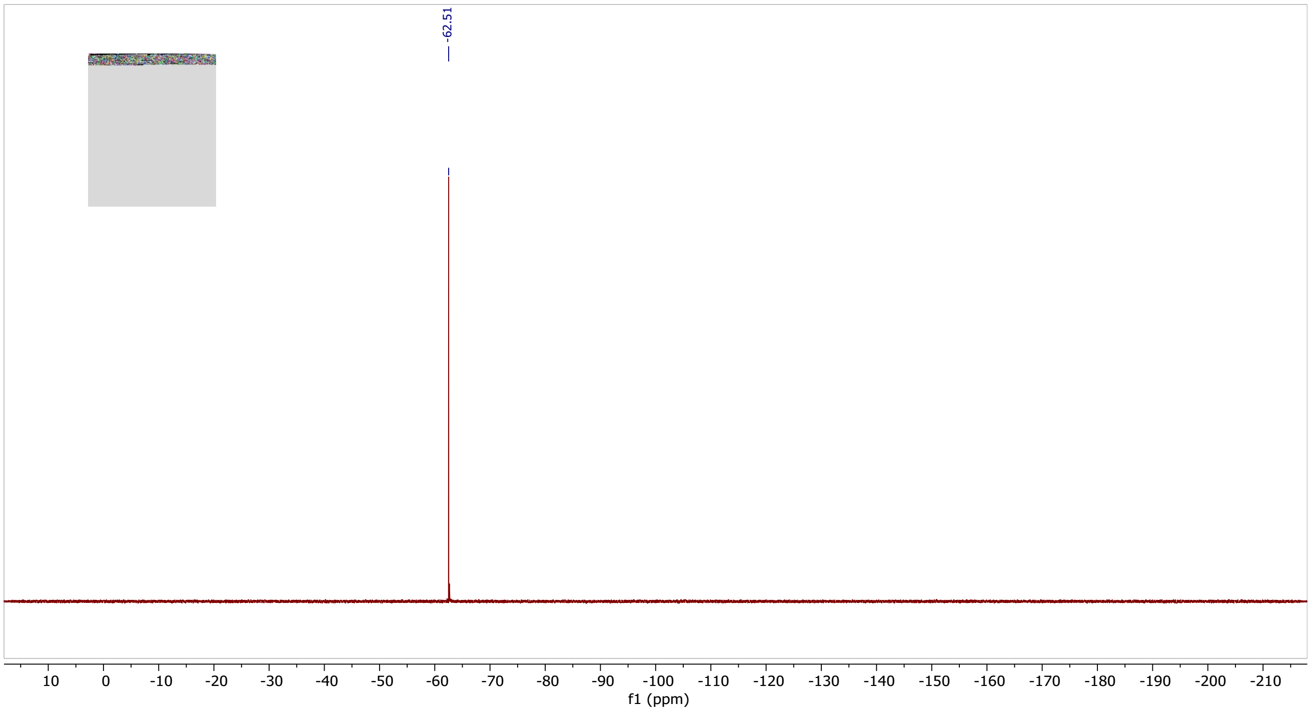

^{1}H NMR (500 MHz, $CDCl_3$) spectrum of **3as**

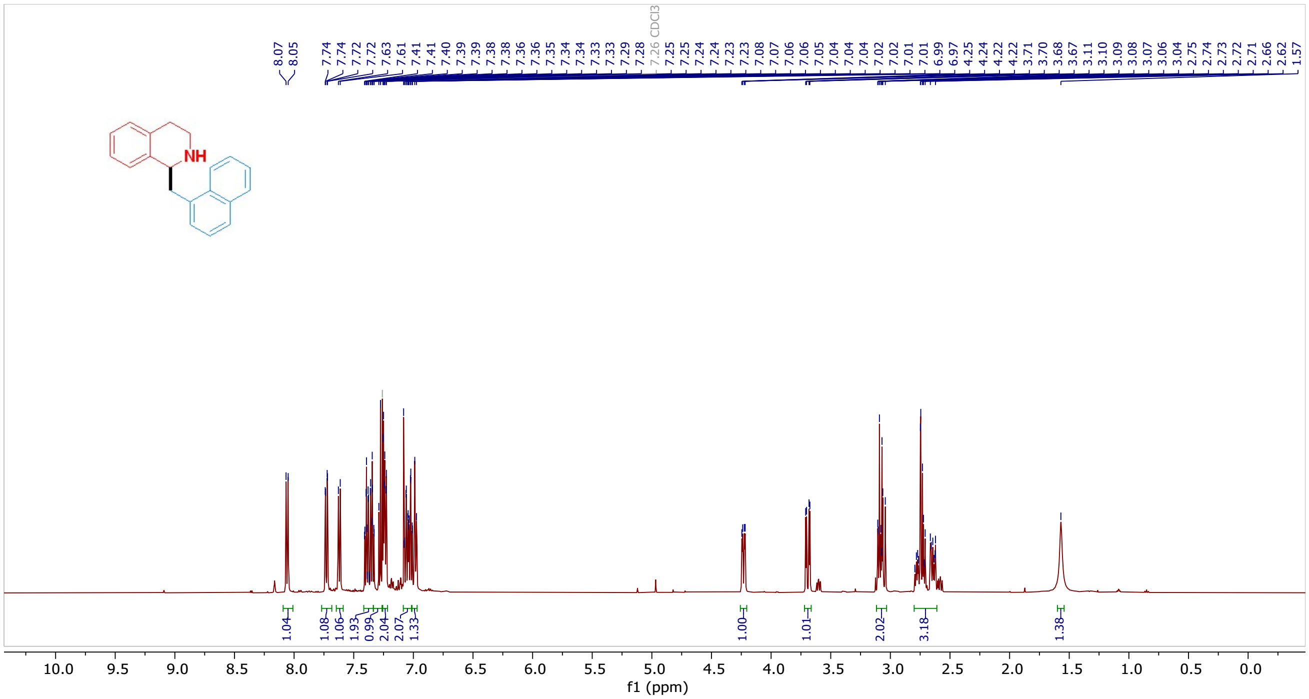

$^{13}C\{^{1}H\}$ NMR (126 MHz, $CDCl_3$) spectrum of **3as**

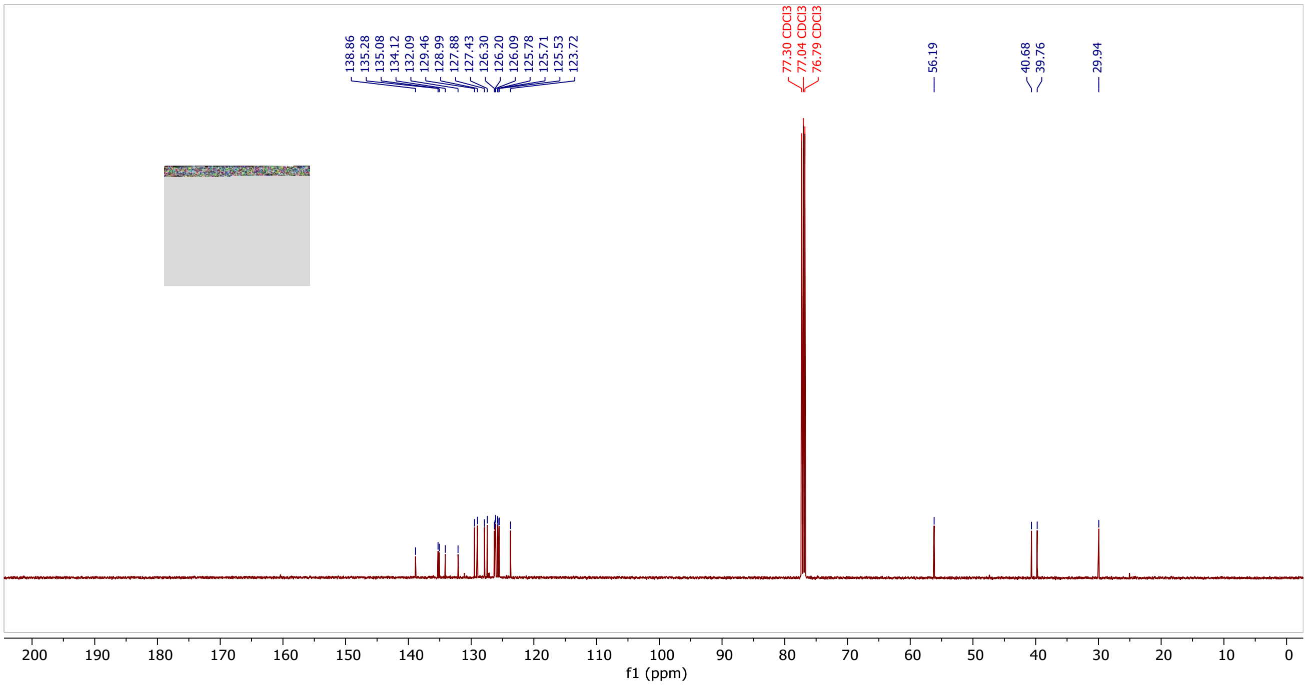

^{1}H NMR (500 MHz, $CDCl_3$) spectrum of **3at**

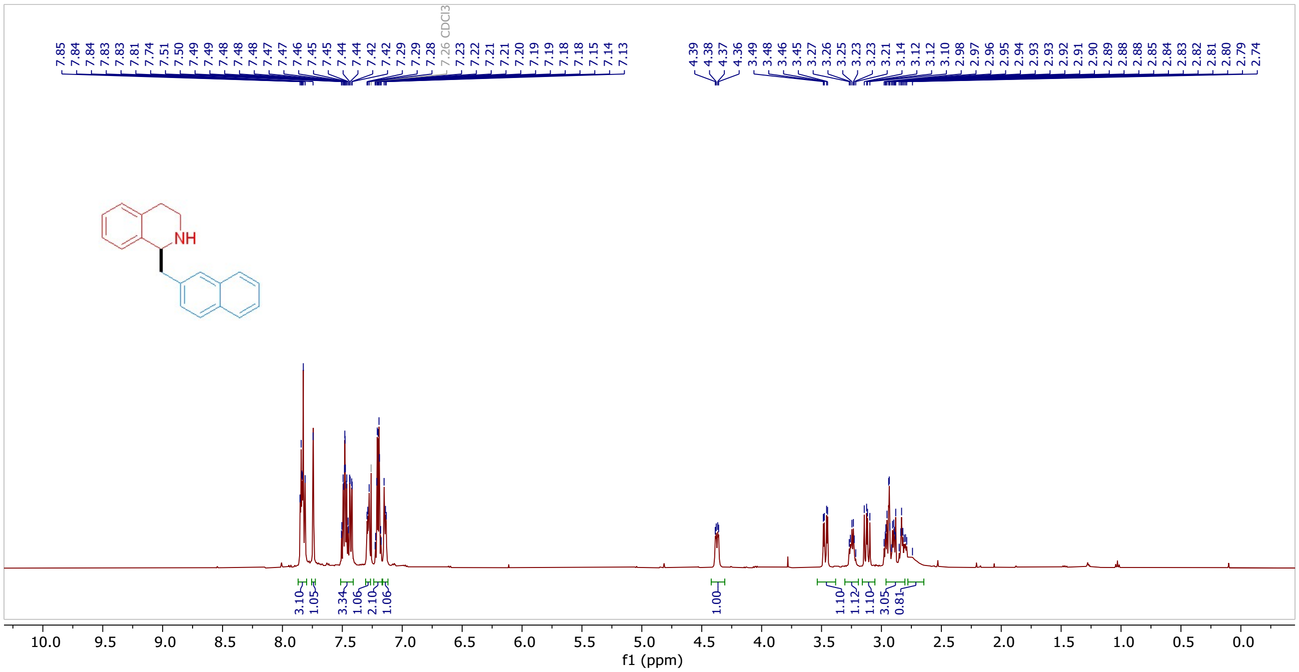

$^{13}C\{^{1}H\}$ NMR (126 MHz, $CDCl_3$) spectrum of **3at**

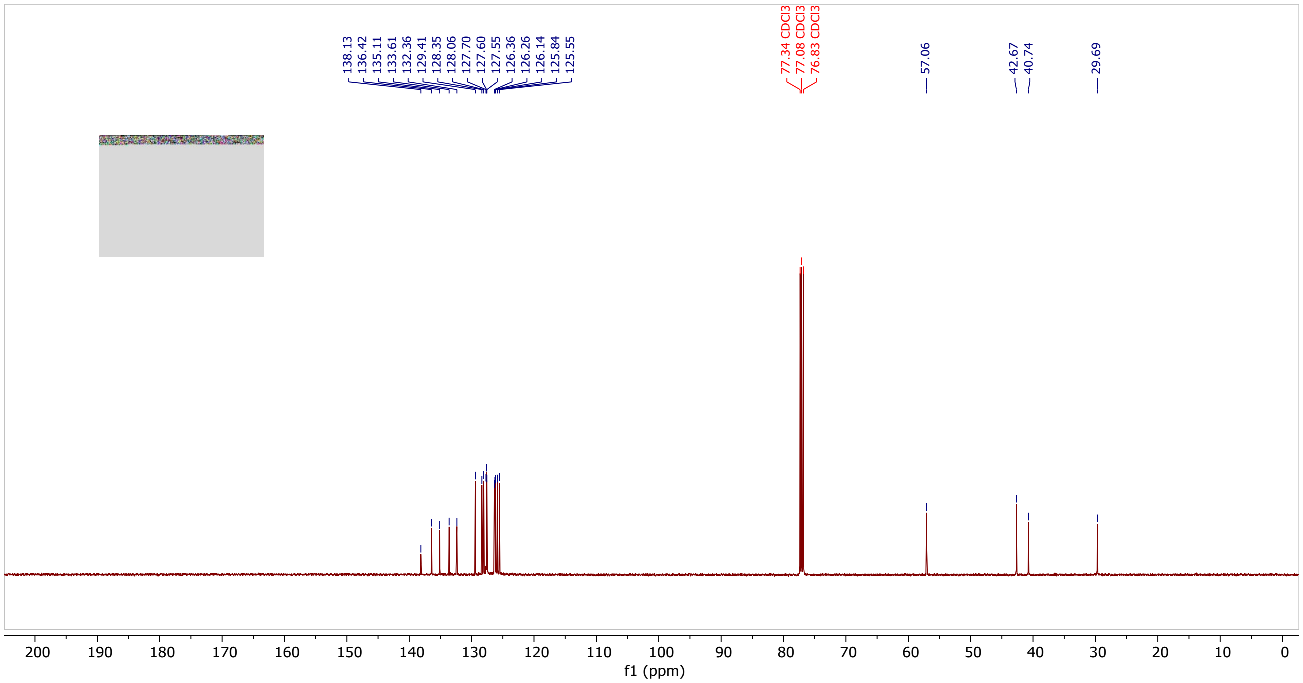

^{1}H NMR (500 MHz, $CDCl_3$) spectrum of **3au**

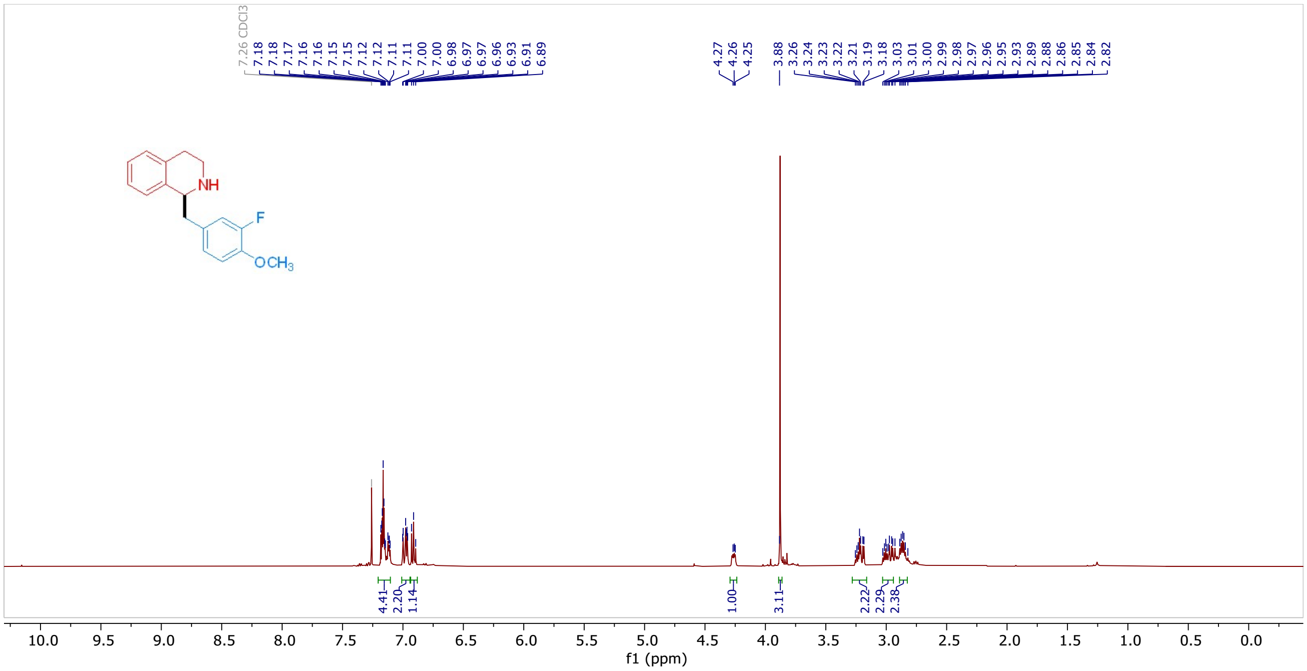

$^{13}C\{^{1}H\}$ NMR (126 MHz, $CDCl_3$) spectrum of **3au**

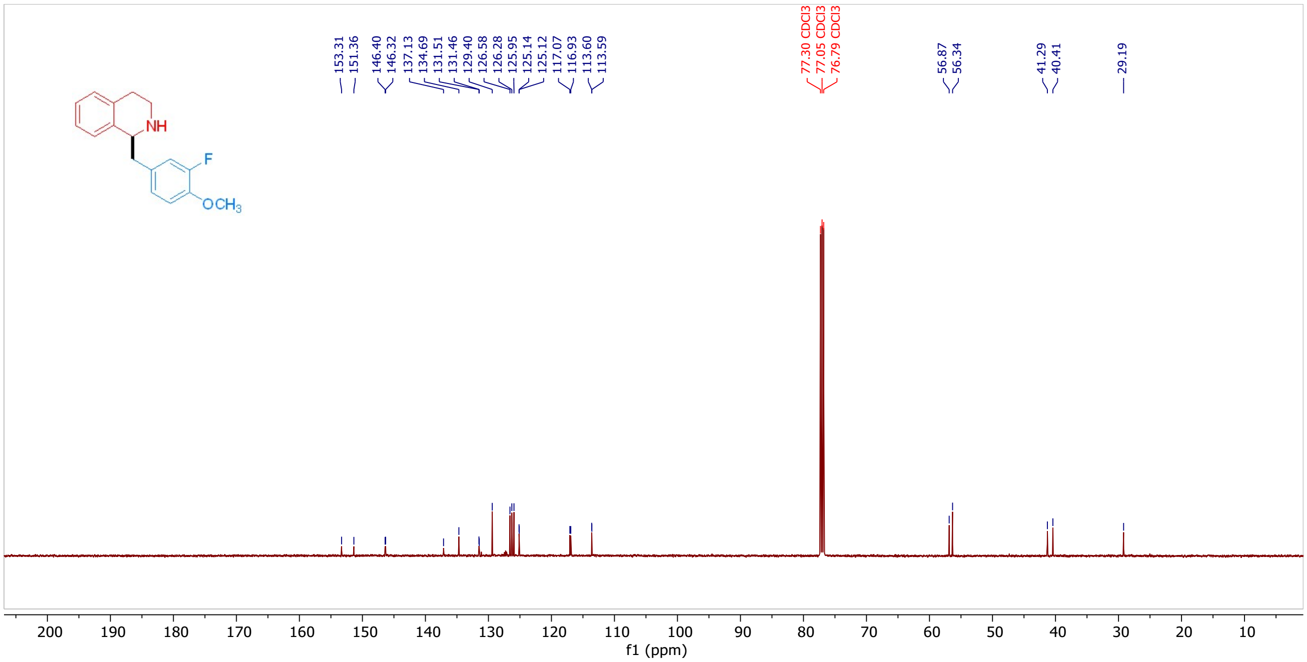

^{19}F NMR (471 MHz, $CDCl_3$) spectrum of **3au**

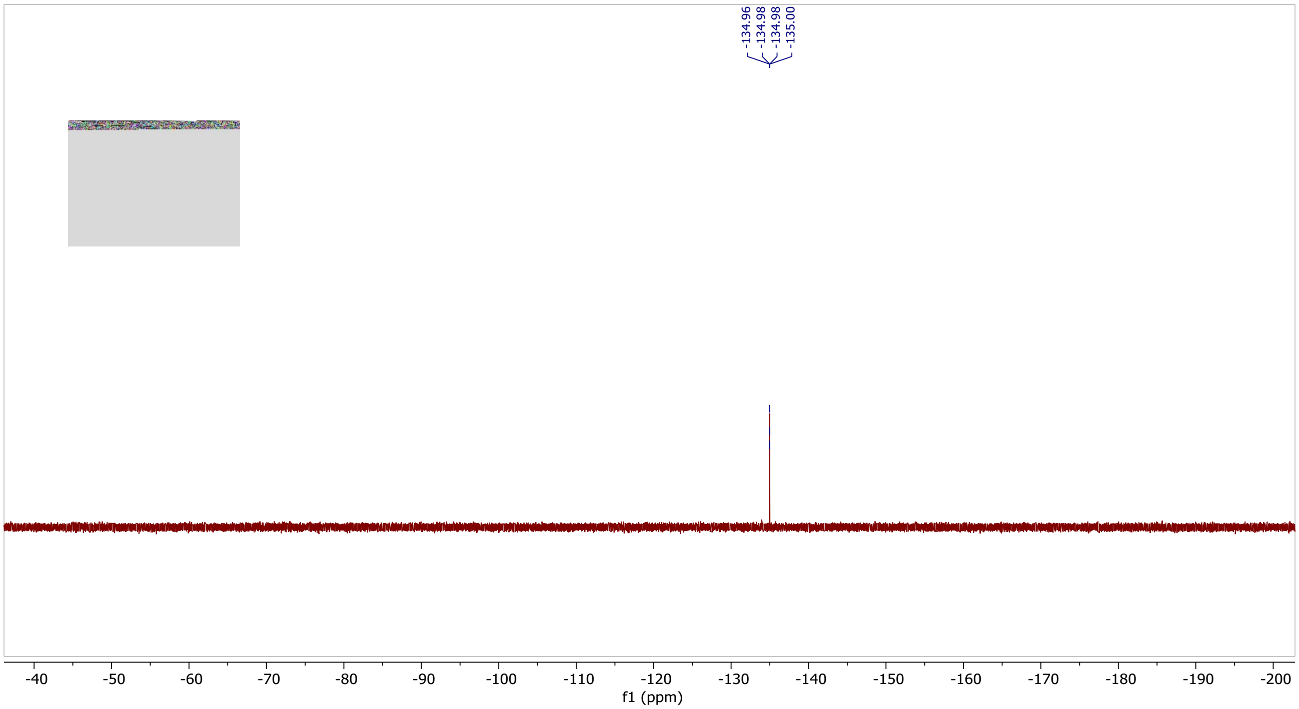

^{1}H NMR (500 MHz, $CDCl_3$) spectrum of **3av**

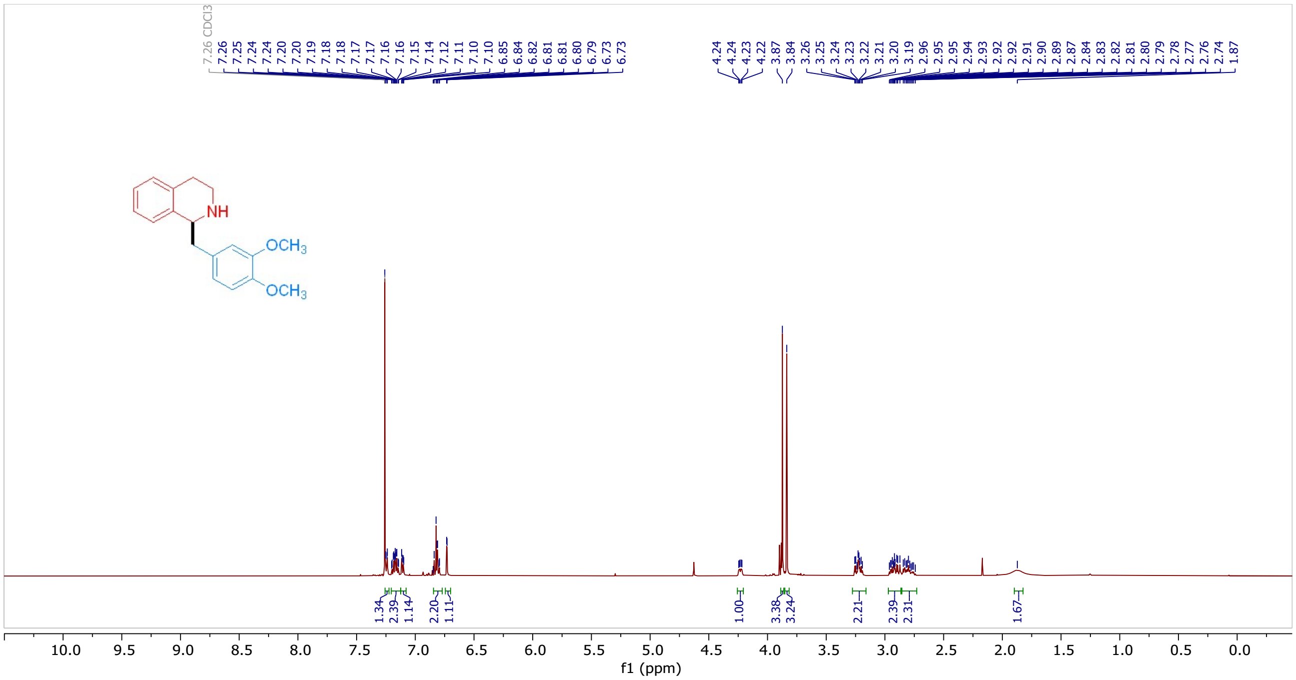

$^{13}C\{^{1}H\}$ NMR (126 MHz, $CDCl_3$) spectrum of **3av**

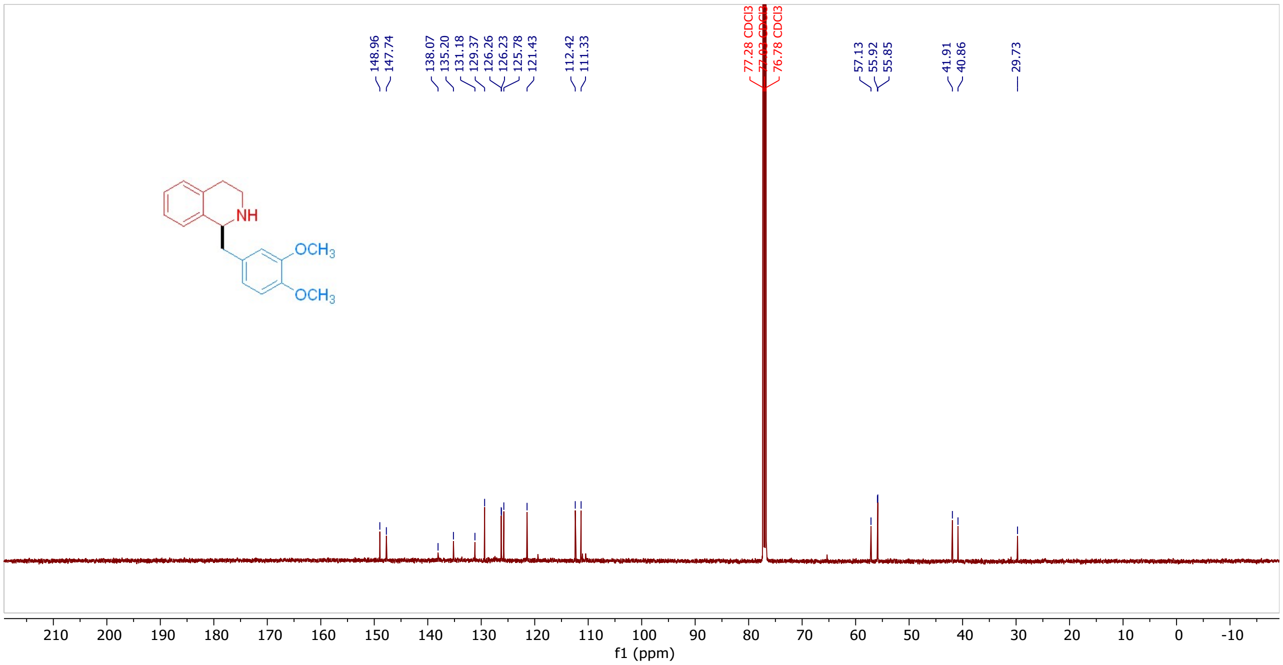

^{1}H NMR (500 MHz, $CDCl_3$) spectrum of **3aw**

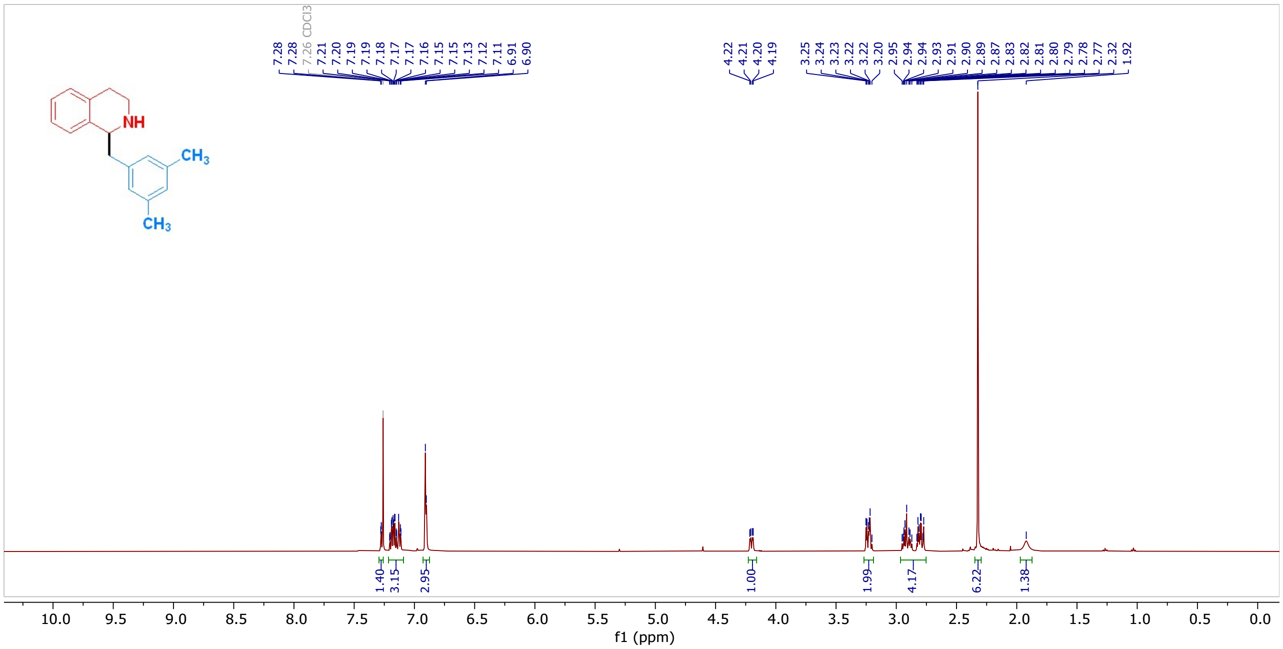

$^{13}C\{^{1}H\}$ NMR (126 MHz, $CDCl_3$) spectrum of **3aw**

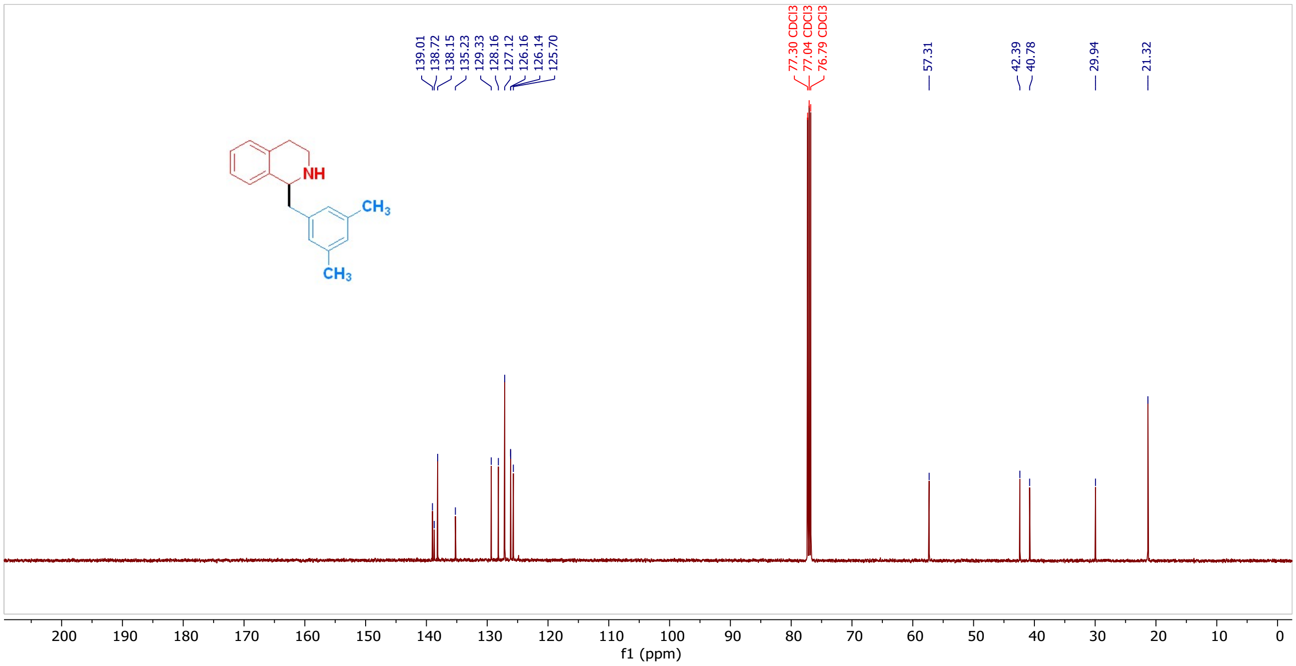

^{1}H NMR (500 MHz, $CDCl_3$) spectrum of **3ax**

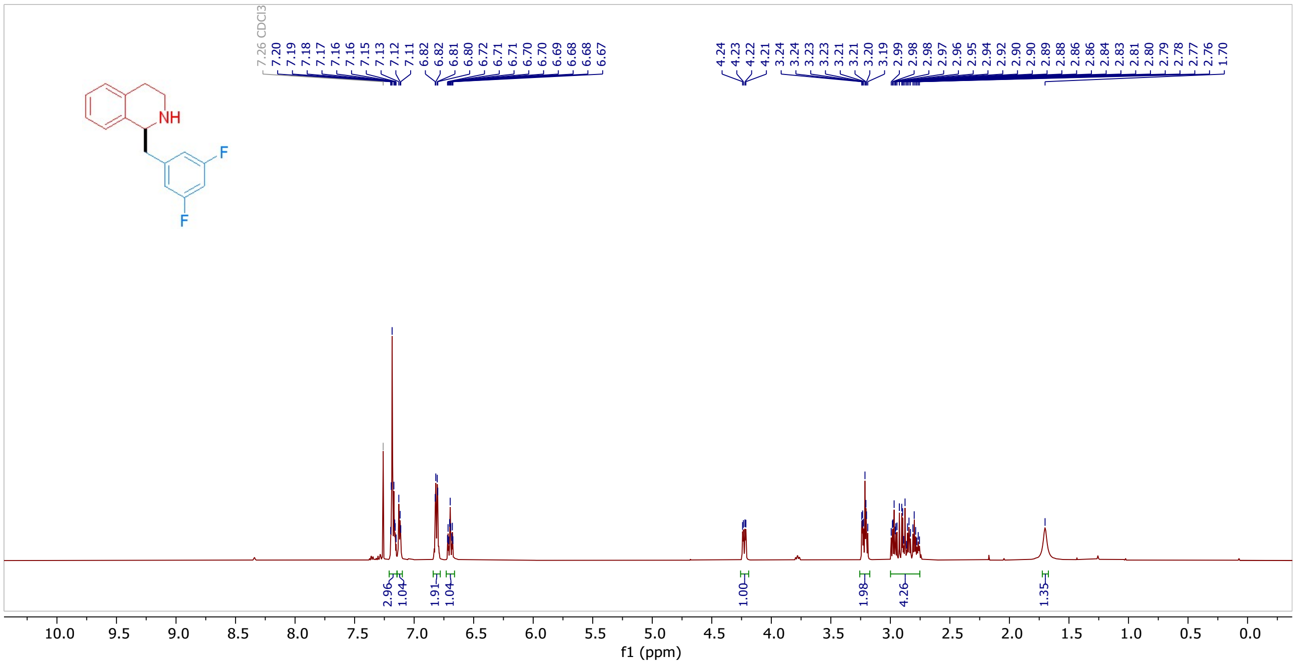

$^{13}C\{^{1}H\}$ NMR (126 MHz, $CDCl_3$) spectrum of **3ax**

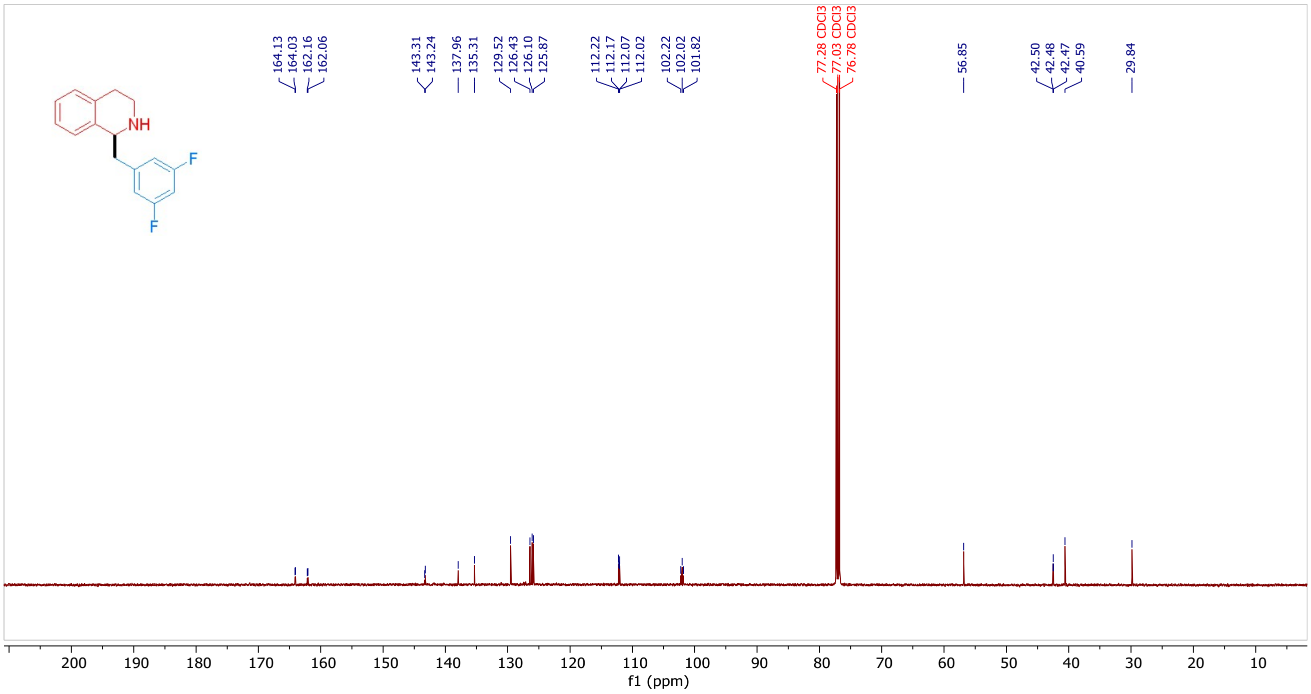

^{19}F NMR (471 MHz, $CDCl_3$) spectrum of **3ax**

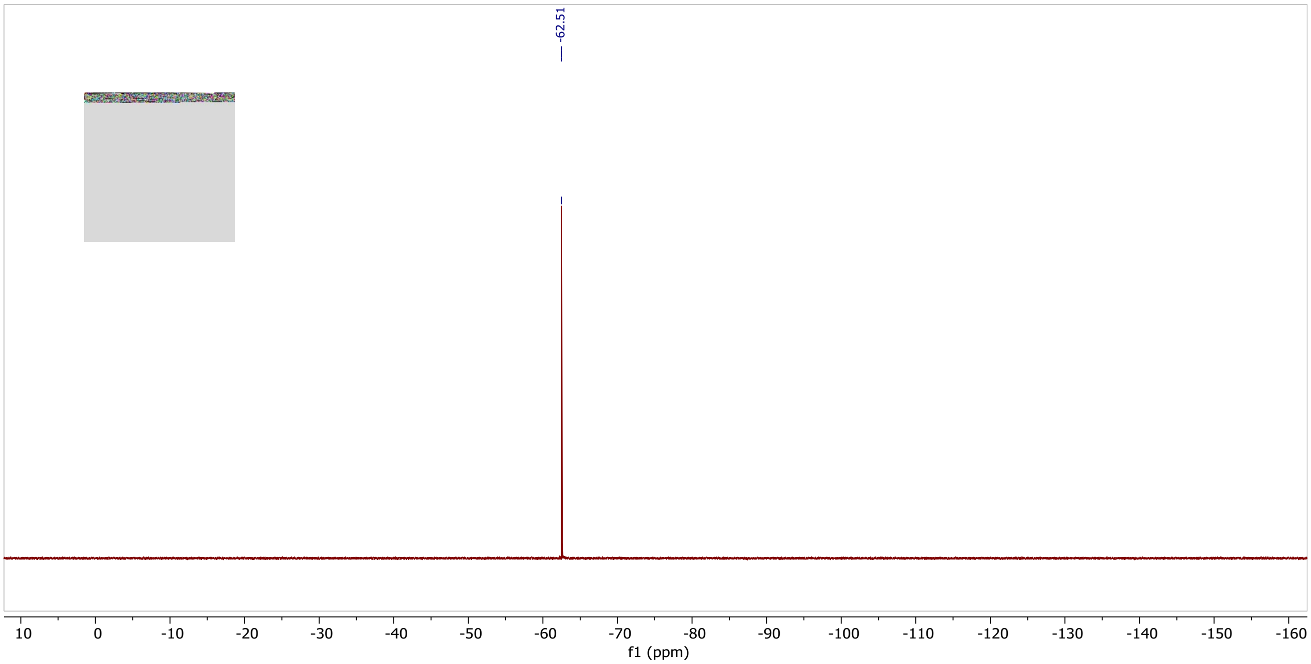

^{1}H NMR (500 MHz, $CDCl_3$) spectrum of **3ay**

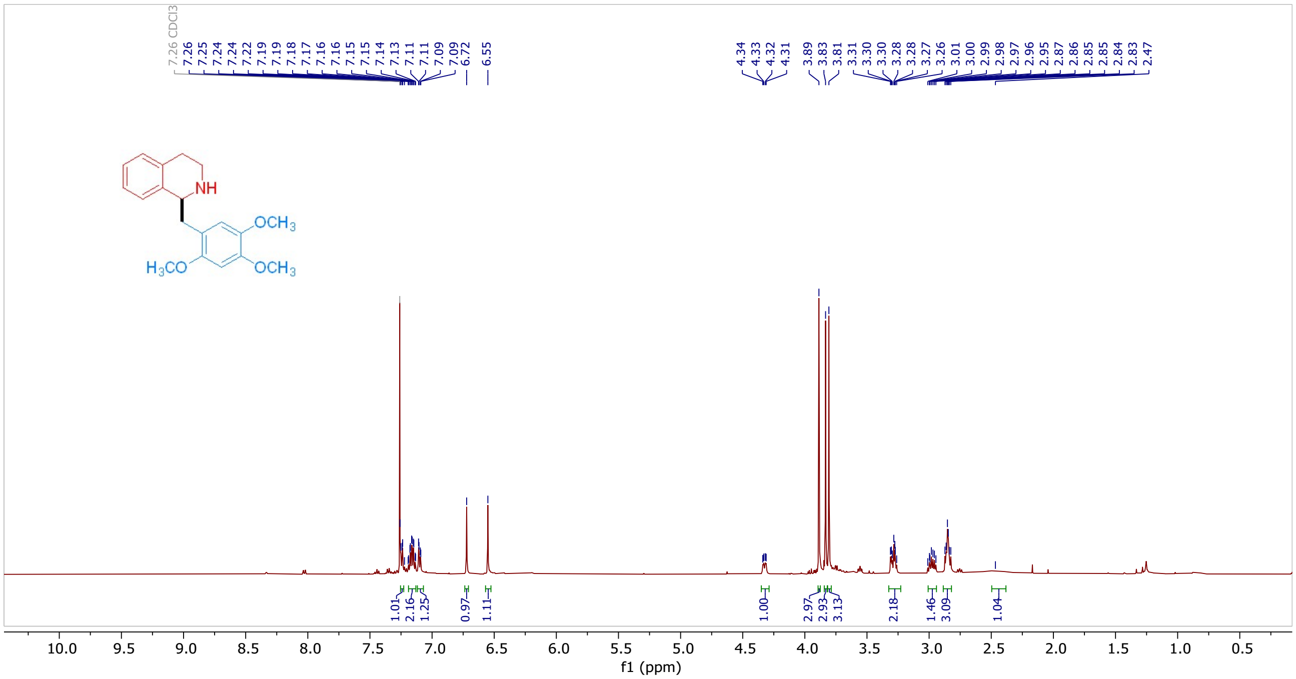

$^{13}C\{^{1}H\}$ NMR (126 MHz, $CDCl_3$) spectrum of **3ay**

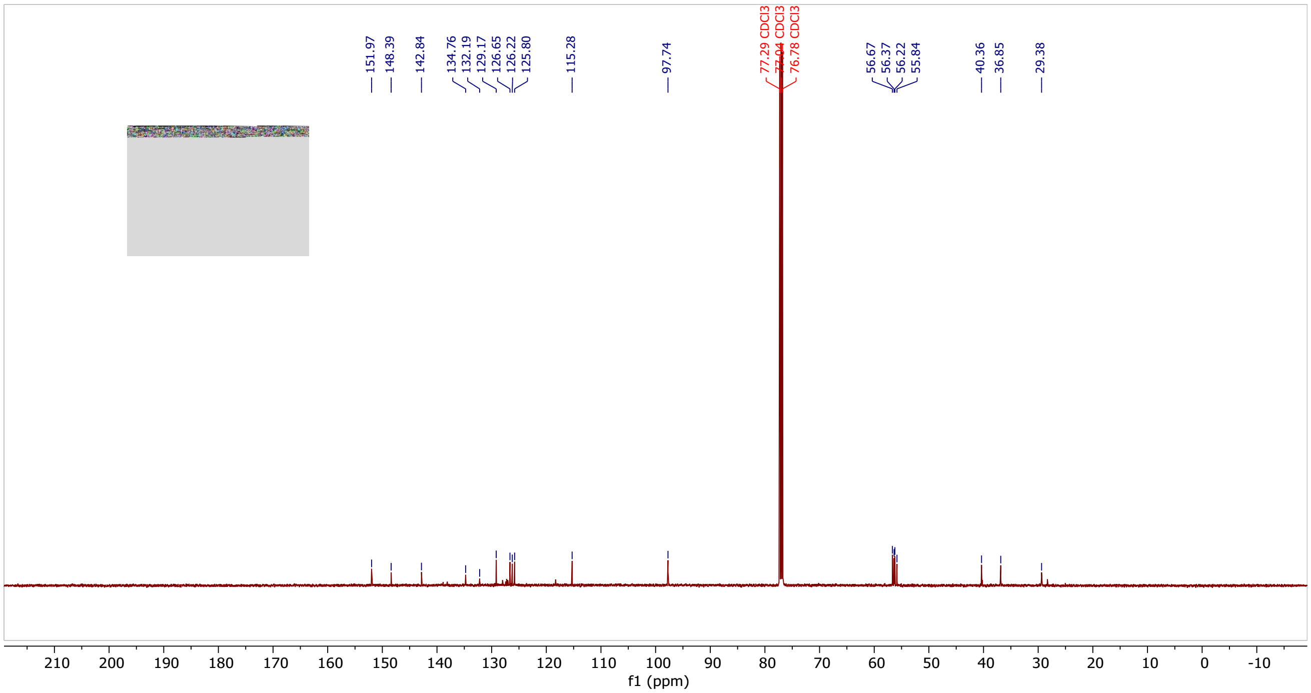

^{1}H NMR (500 MHz, $CDCl_3$) spectrum of **3az**

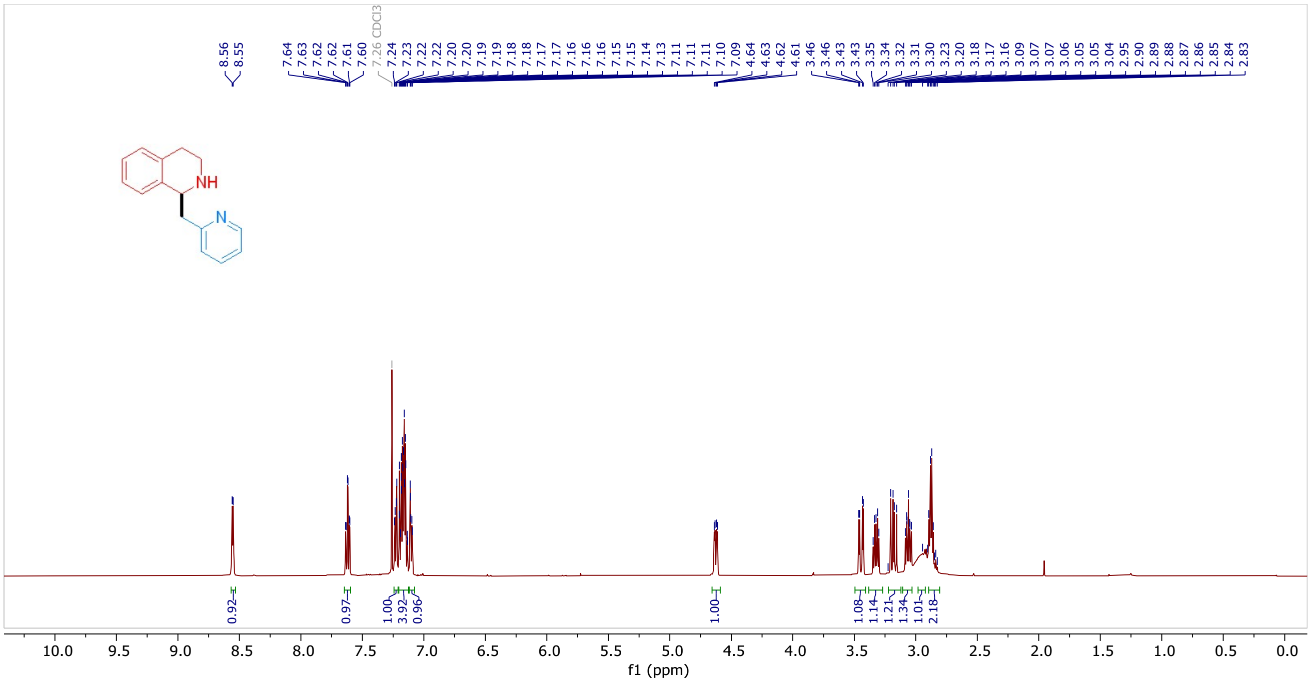

$^{13}C\{^{1}H\}$ NMR (126 MHz, $CDCl_3$) spectrum of **3az**

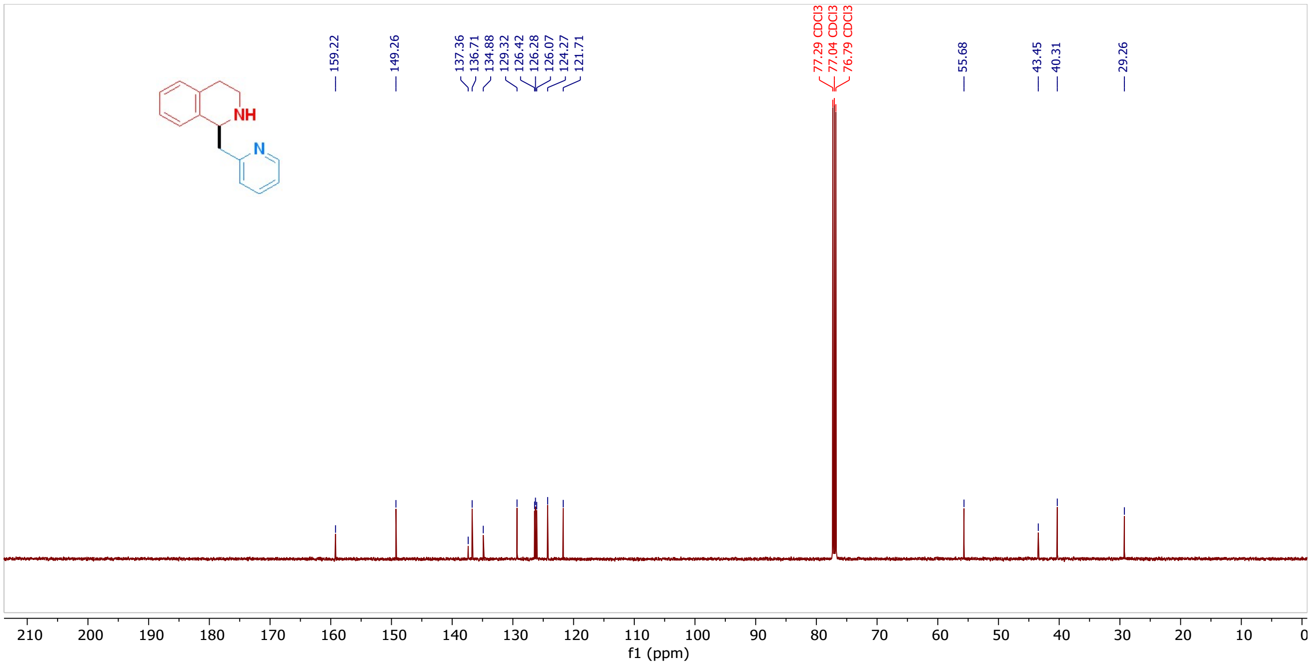

^{1}H NMR (500 MHz, $CDCl_3$) spectrum of **3ba**

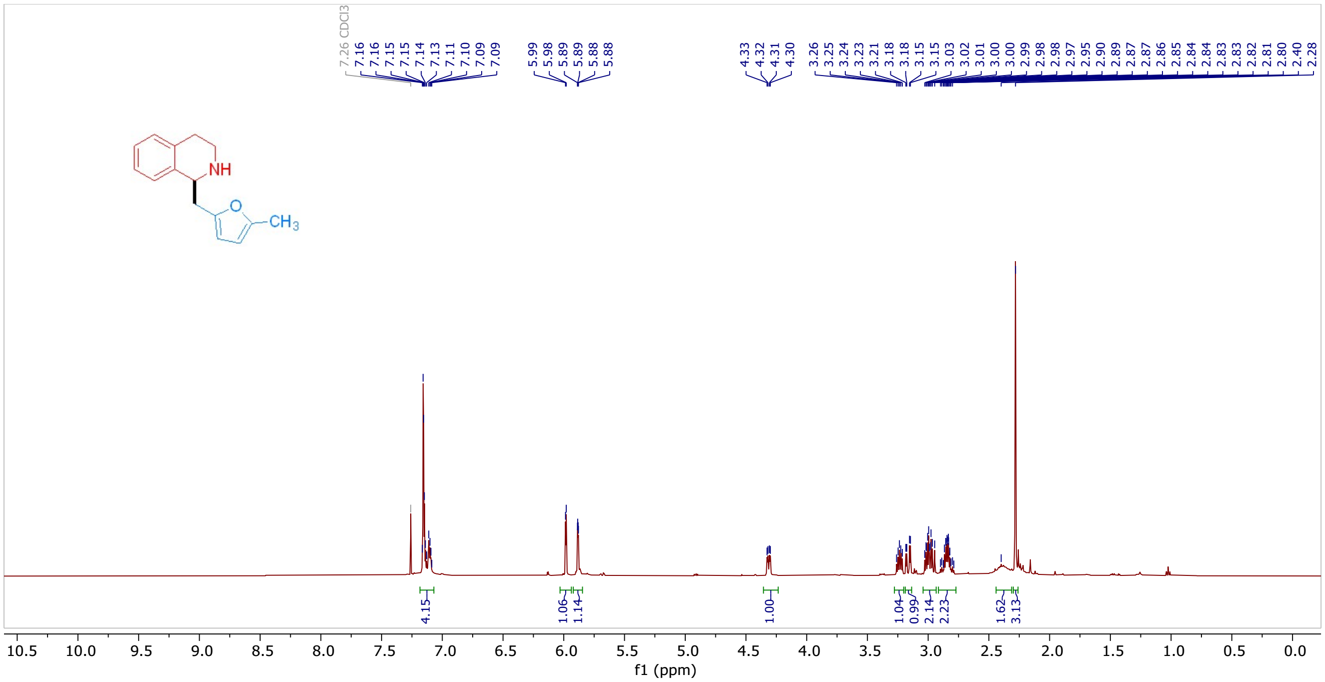

$^{13}C\{^1H\}$ NMR (126 MHz, $CDCl_3$) spectrum of **3ba**

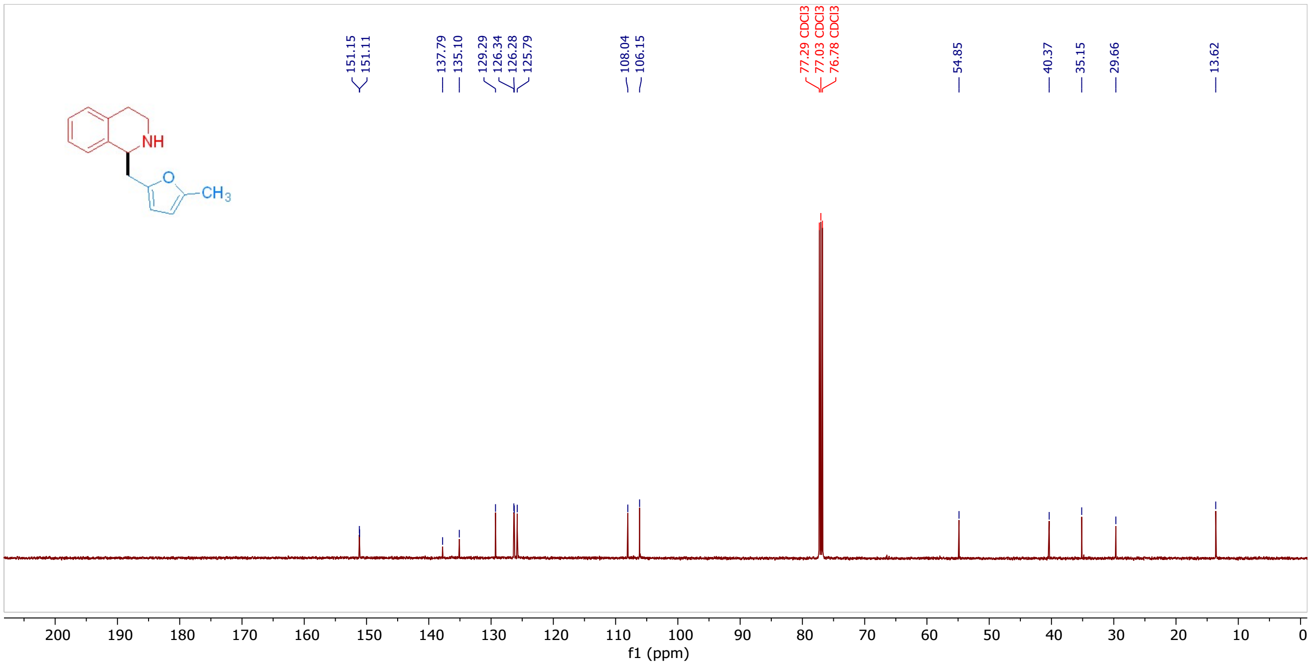

^{1}H NMR (500 MHz, $CDCl_3$) spectrum of **3bb**

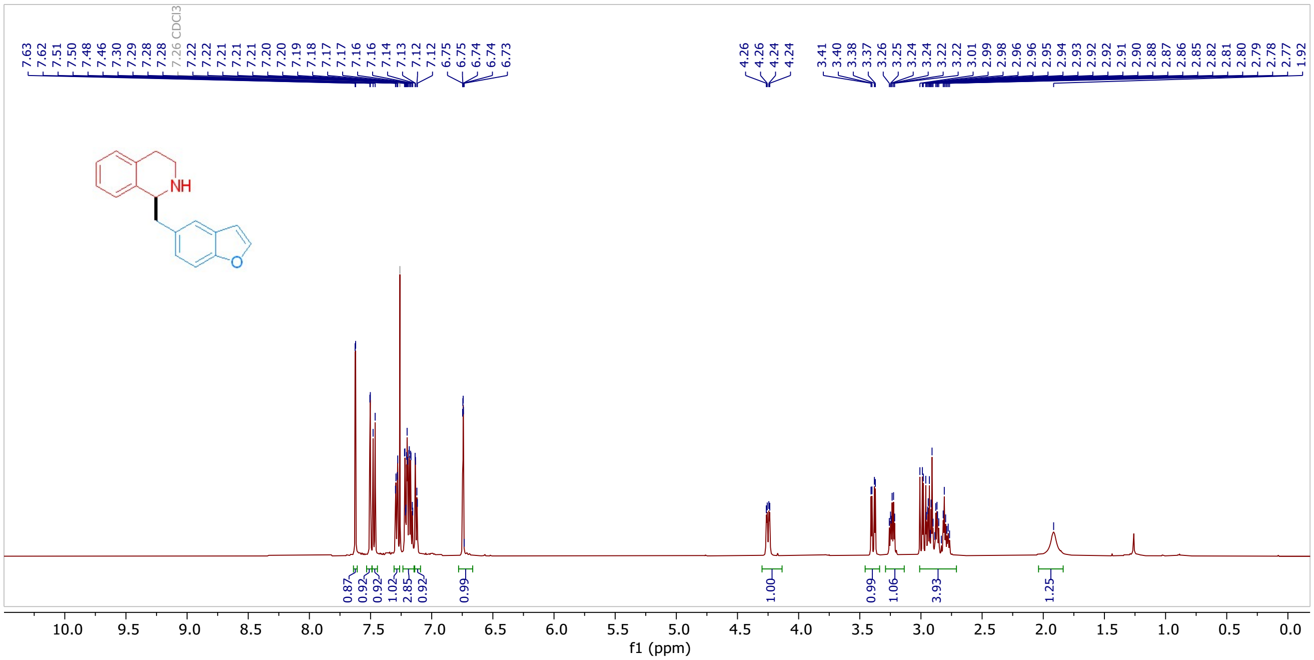

$^{13}C\{^{1}H\}$ NMR (126 MHz, $CDCl_3$) spectrum of **3bb**

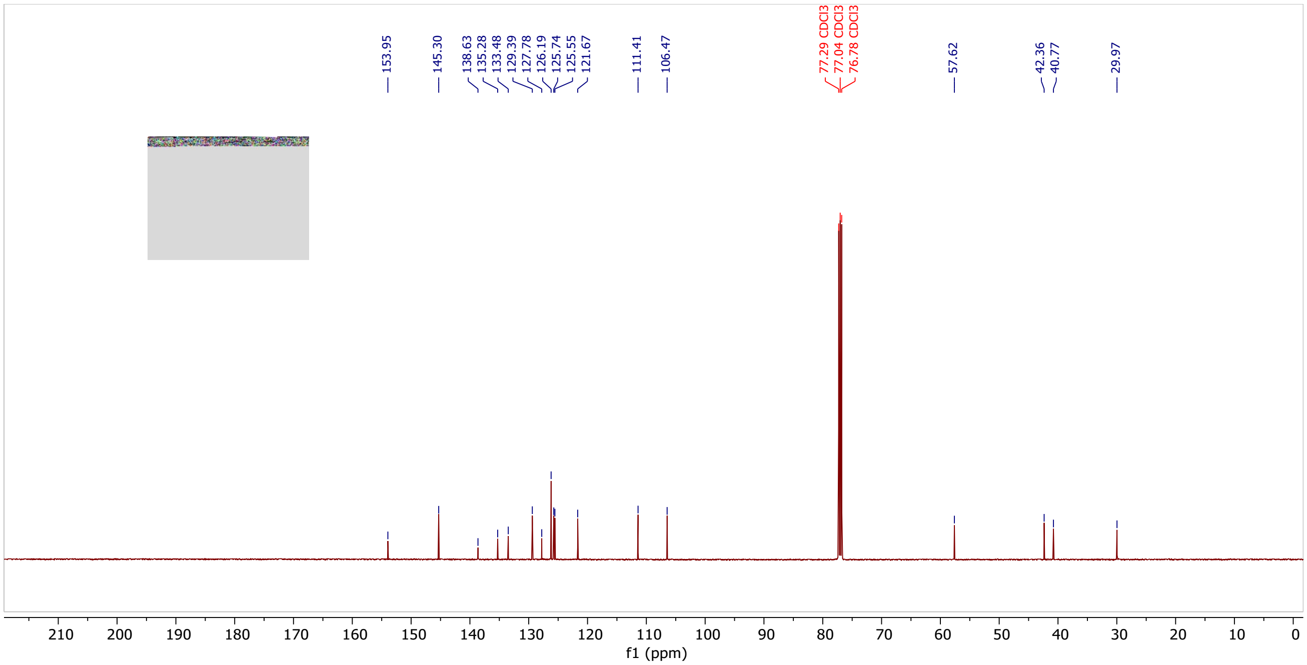

^{1}H NMR (500 MHz, $CDCl_3$) spectrum of **3be**

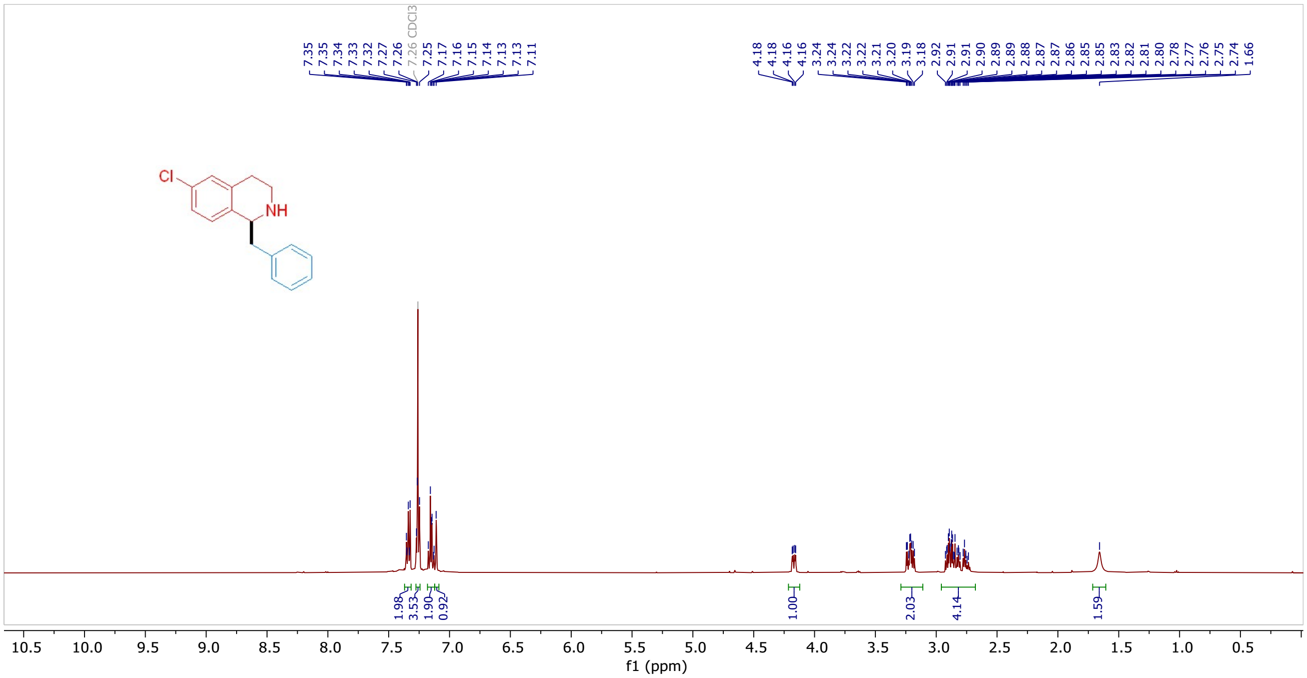

$^{13}C\{^{1}H\}$ NMR (126 MHz, $CDCl_3$) spectrum of **3be**

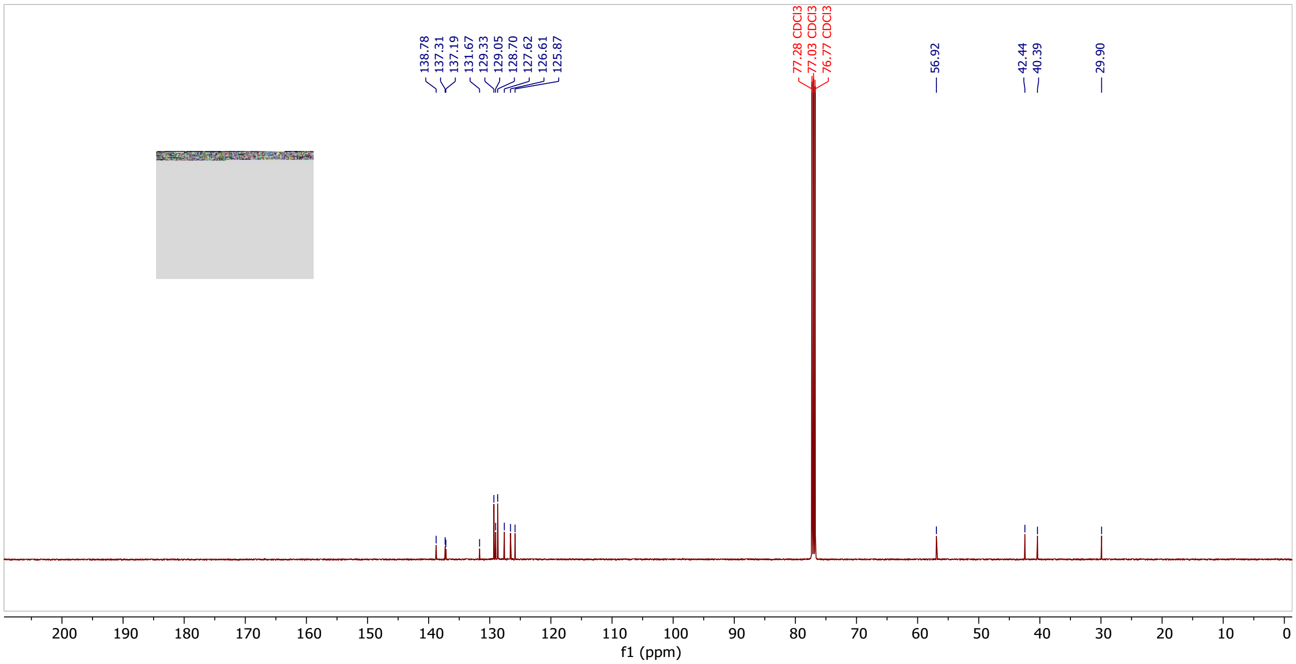

^{1}H NMR (500 MHz, $CDCl_3$) spectrum of **3bf**

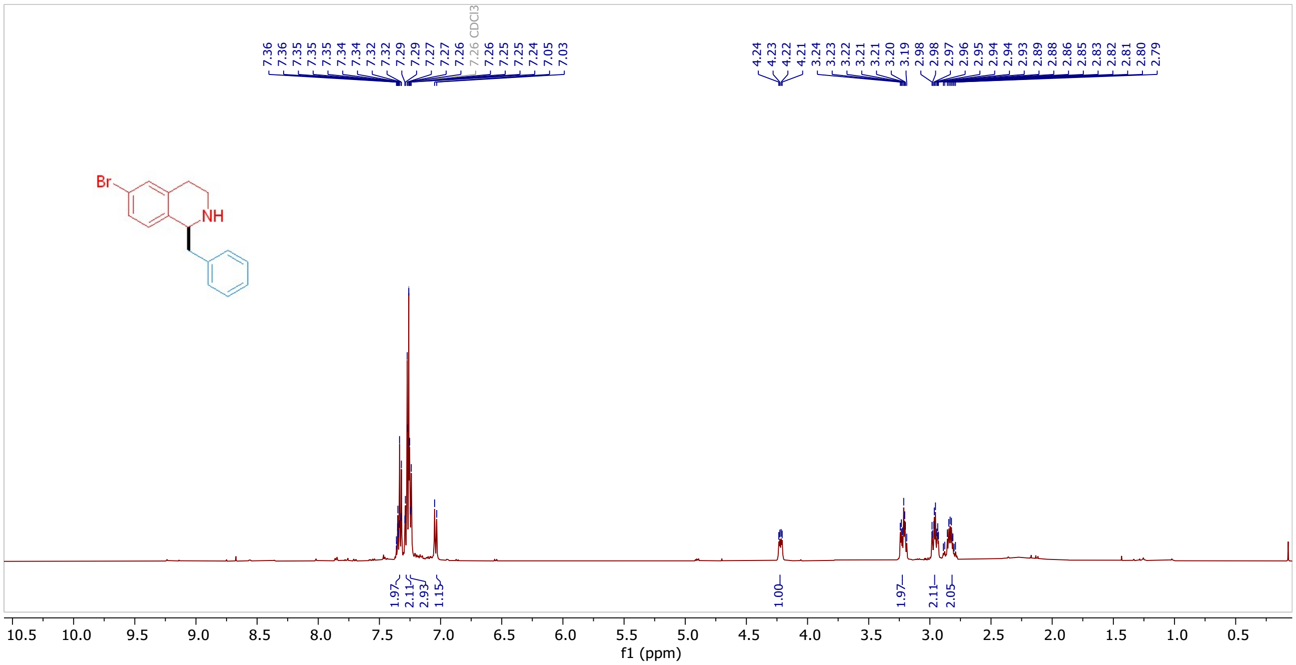

$^{13}C\{^{1}H\}$ NMR (126 MHz, $CDCl_3$) spectrum of **3bf**

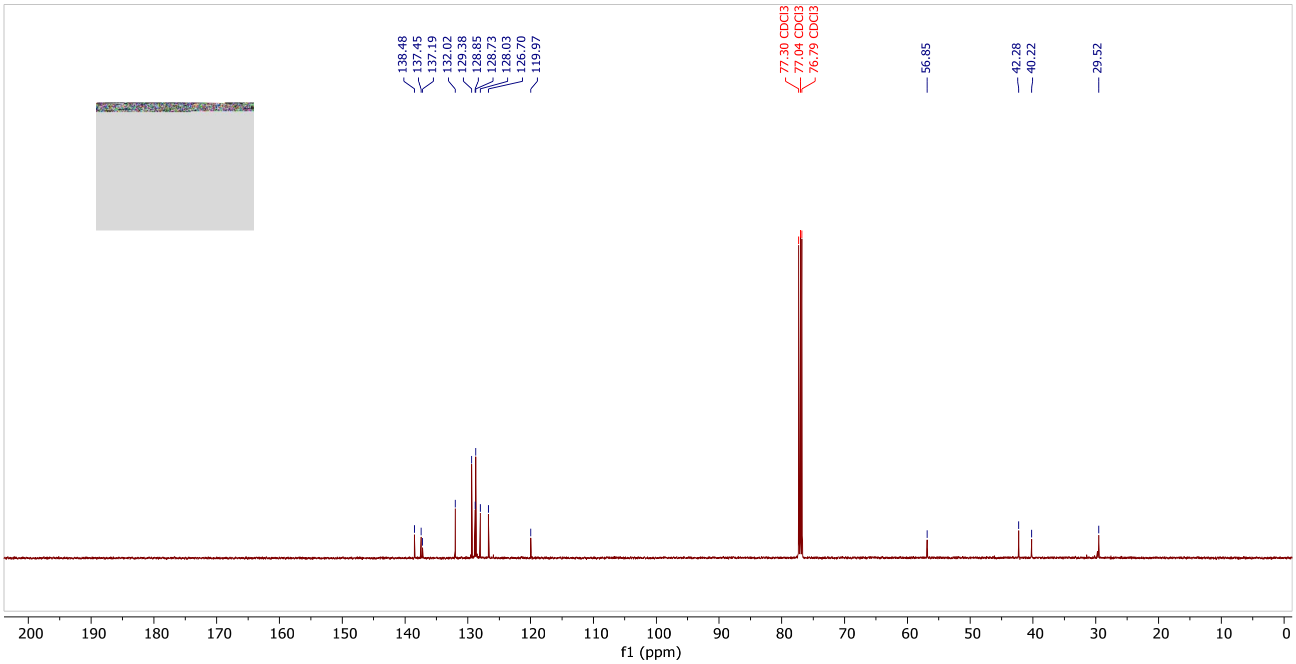

^{1}H NMR (500 MHz, $CDCl_3$) spectrum of **3bg**

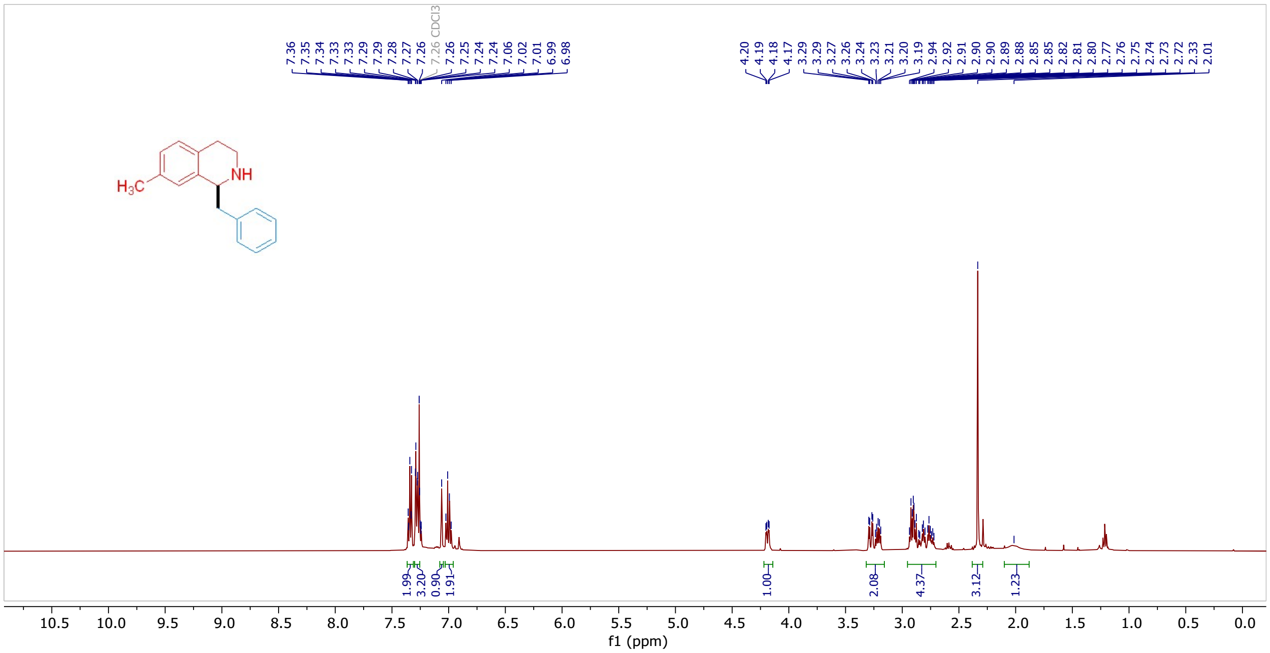

$^{13}C\{^1H\}$ NMR (126 MHz, $CDCl_3$) spectrum of **3bg**

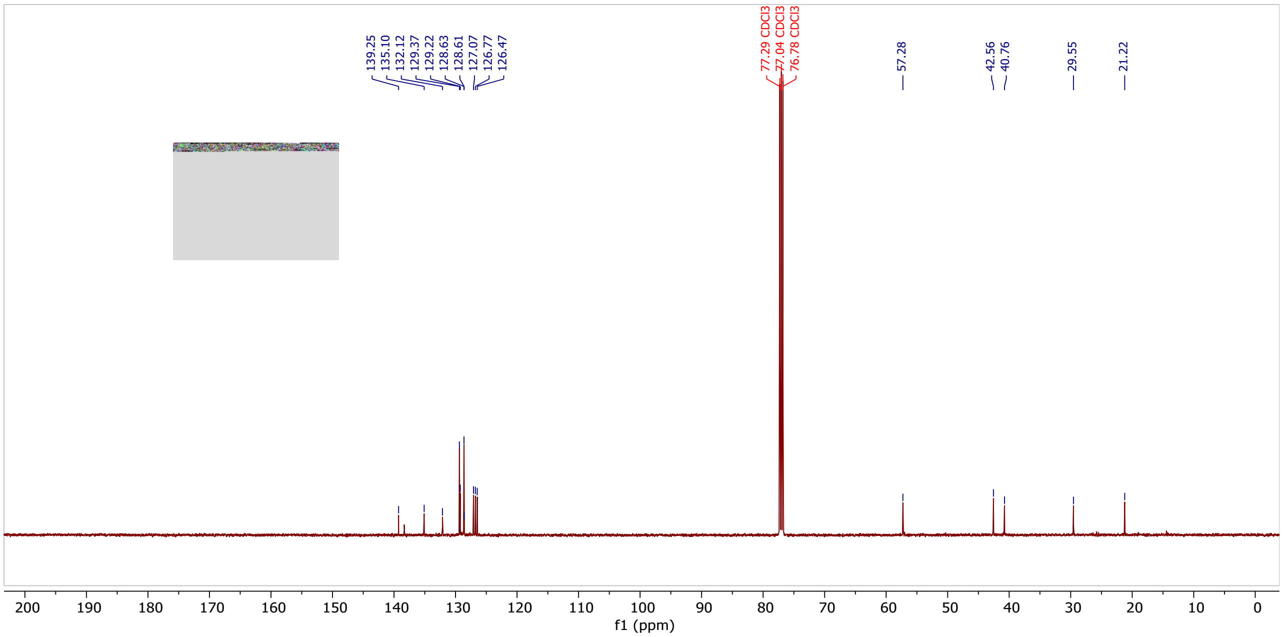

^1H NMR (500 MHz, $CDCl_3$) spectrum of **3bh**

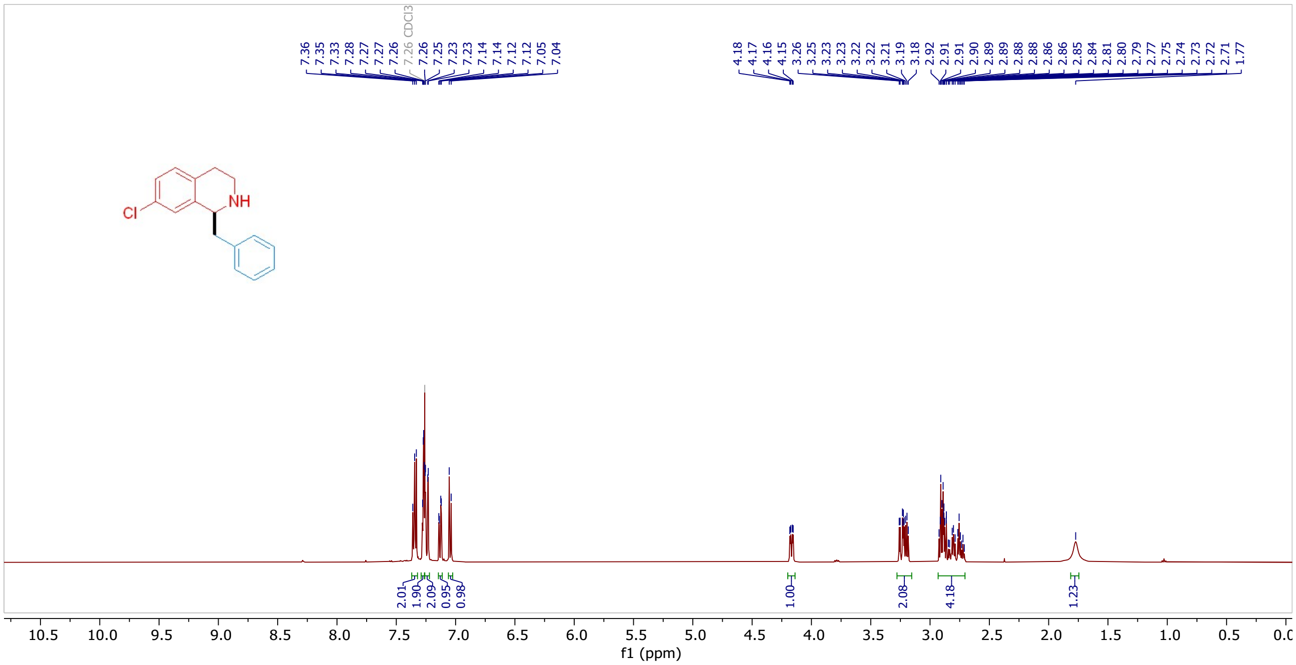

$^{13}C\{^{1}H\}$ NMR (126 MHz, $CDCl_3$) spectrum of **3bh**

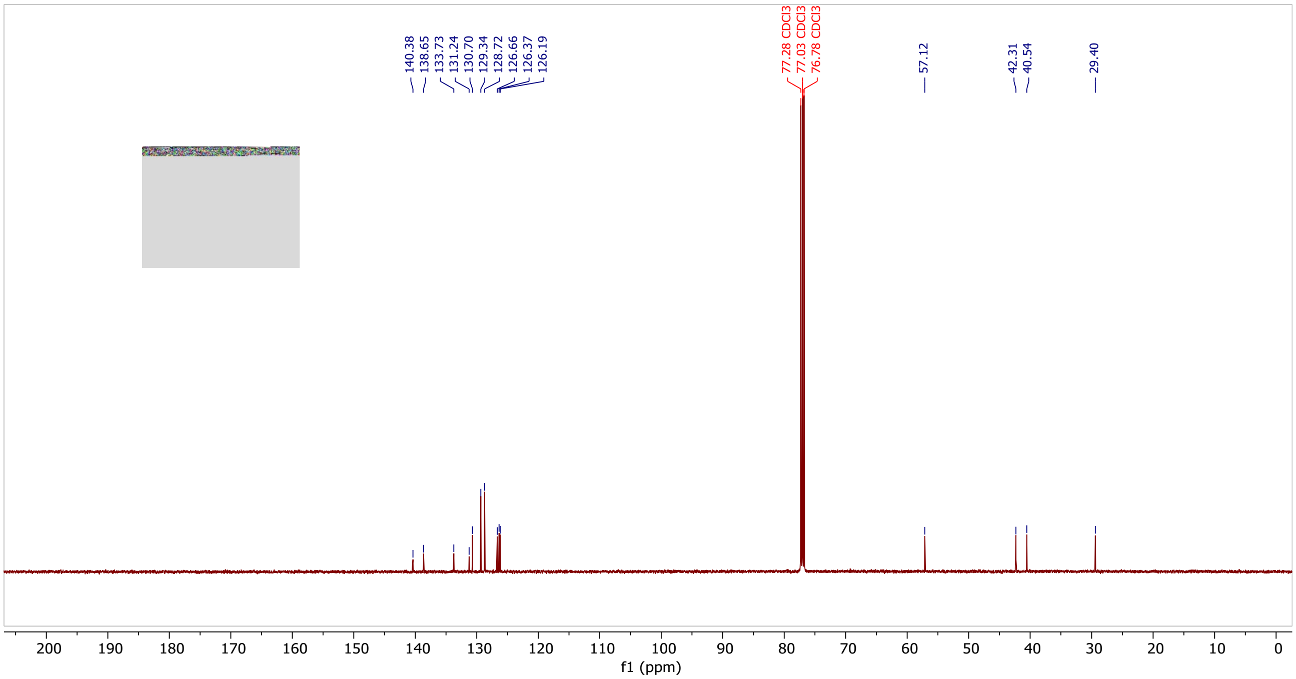

^{1}H NMR (500 MHz, $CDCl_3$) spectrum of **3bk**

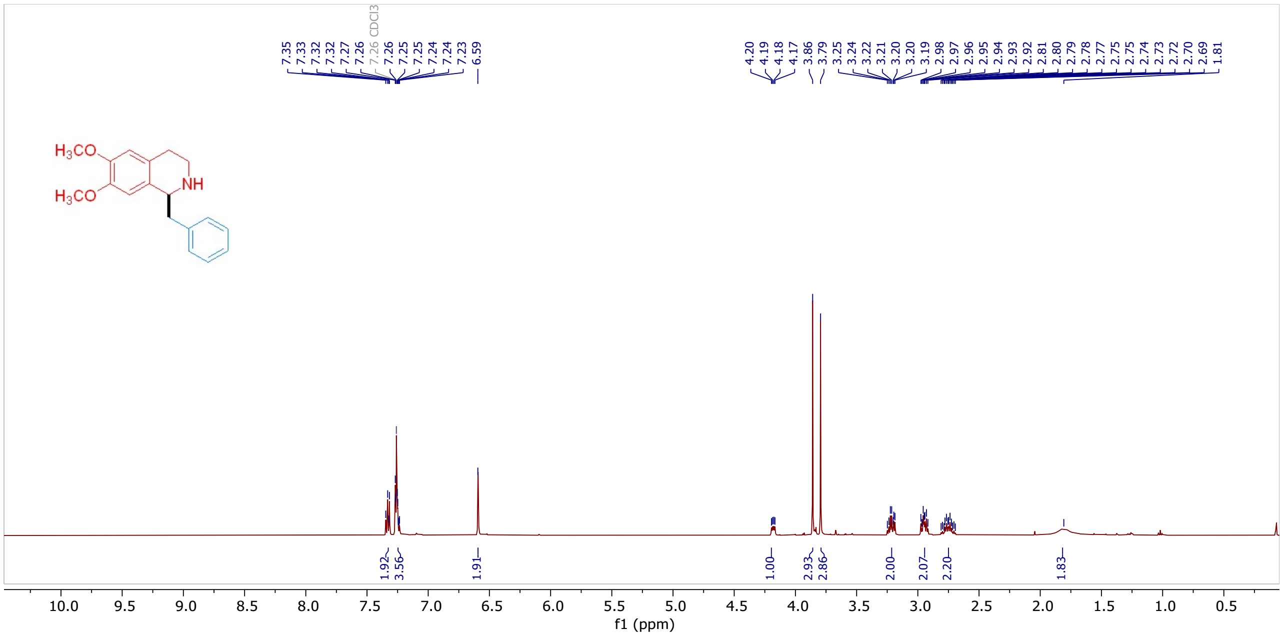

$^{13}C\{^1H\}$ NMR (126 MHz, $CDCl_3$) spectrum of **3bk**

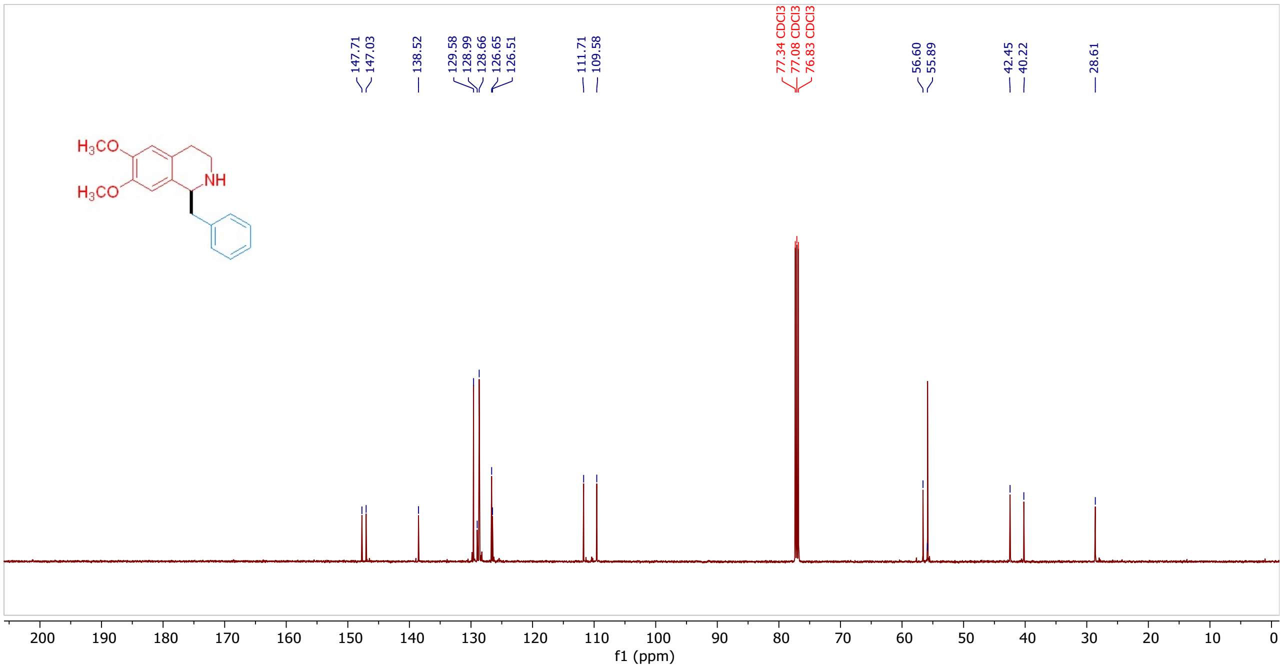

^{1}H NMR (500 MHz, $CDCl_3$) spectrum of **3bl**

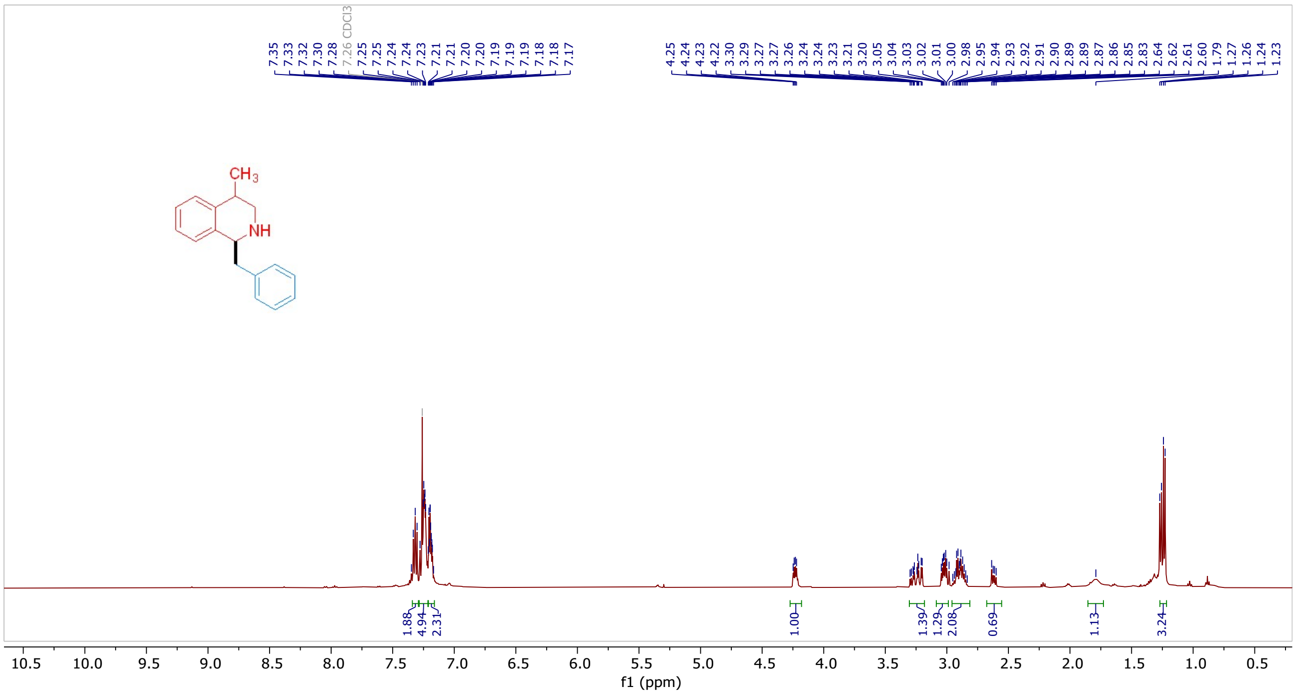

$^{13}C\{^{1}H\}$ NMR (126 MHz, $CDCl_3$) spectrum of **3bl**

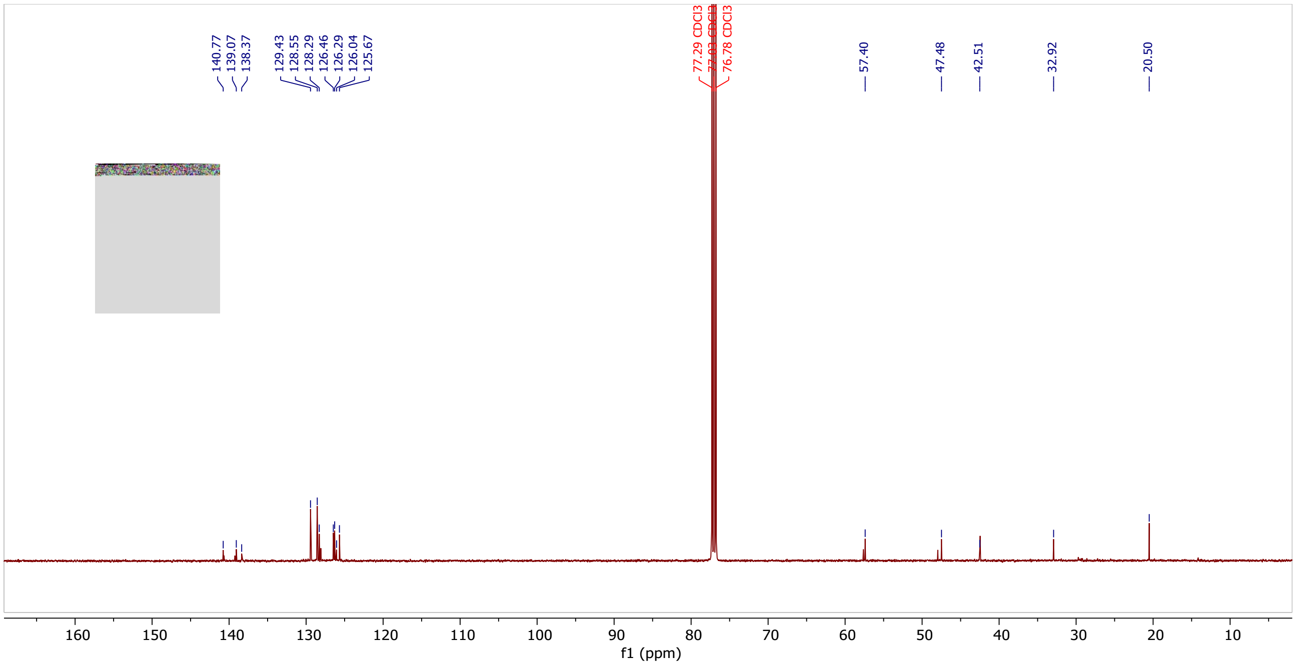

^{1}H NMR (500 MHz, $CDCl_3$) spectrum of **3bm**

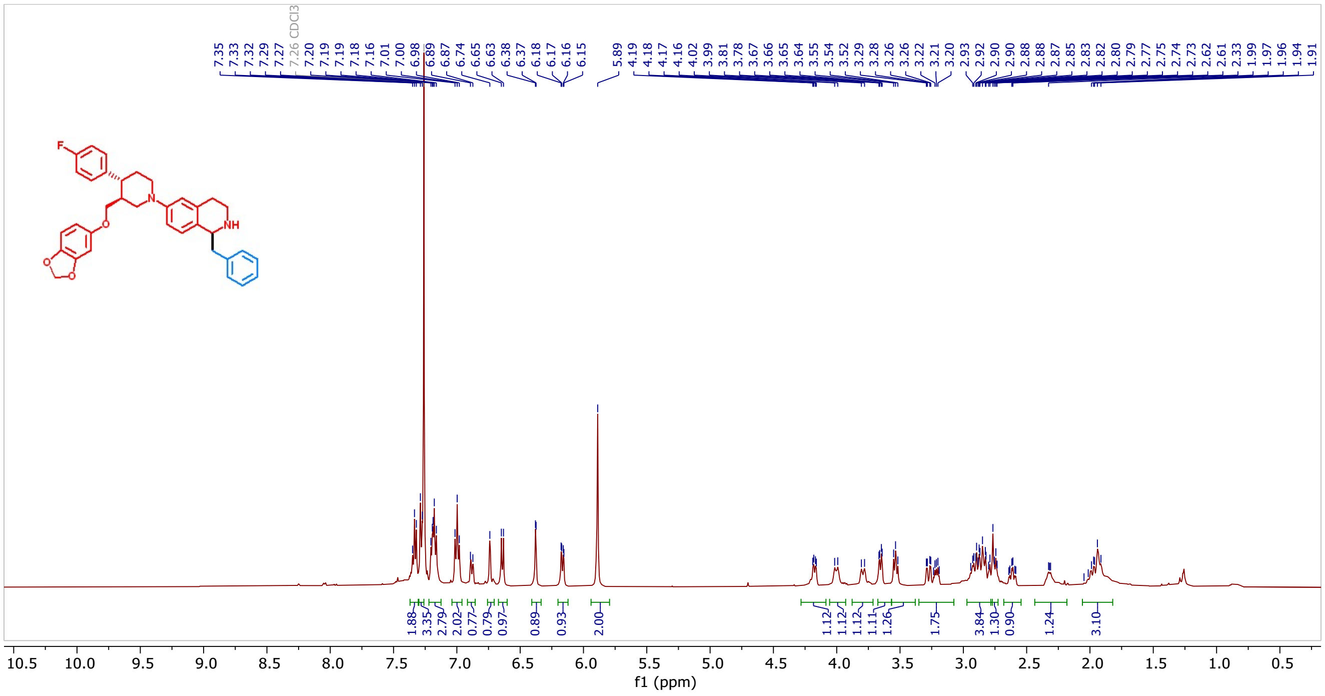

$^{13}C\{^{1}H\}$ NMR (126 MHz, $CDCl_3$) spectrum of **3bm**

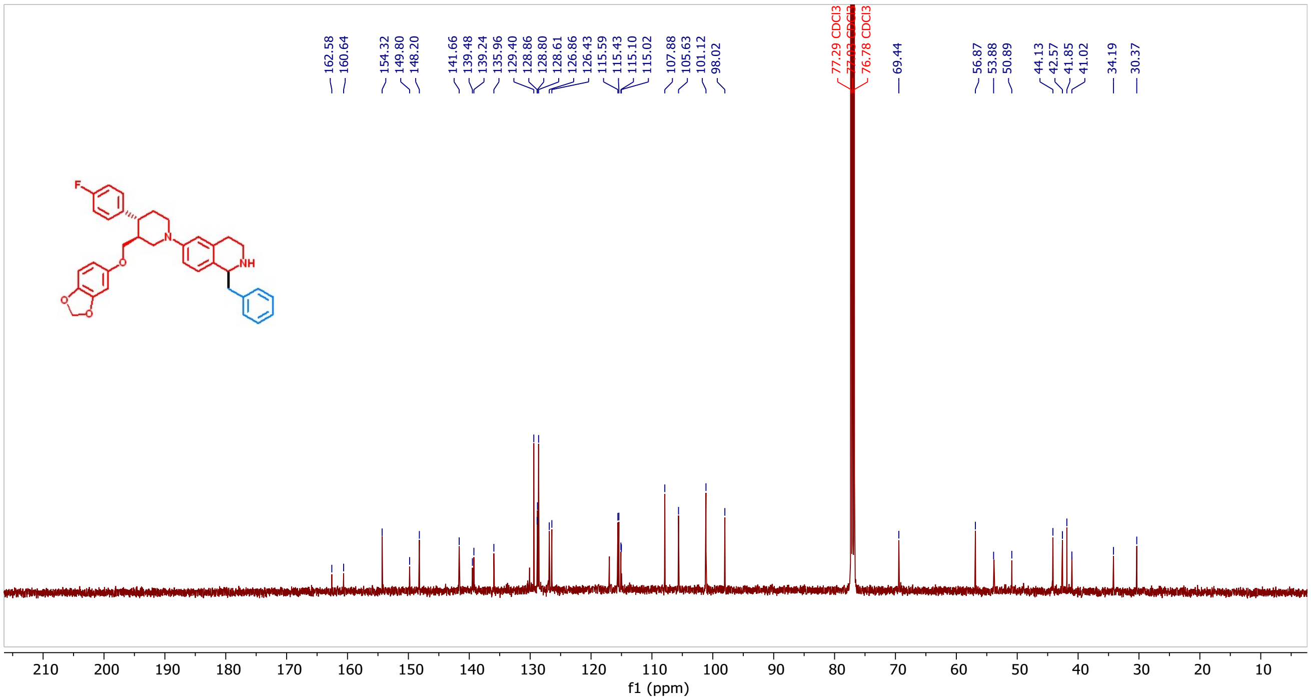

^{19}F NMR (471 MHz, $CDCl_3$) spectrum of **3bm**

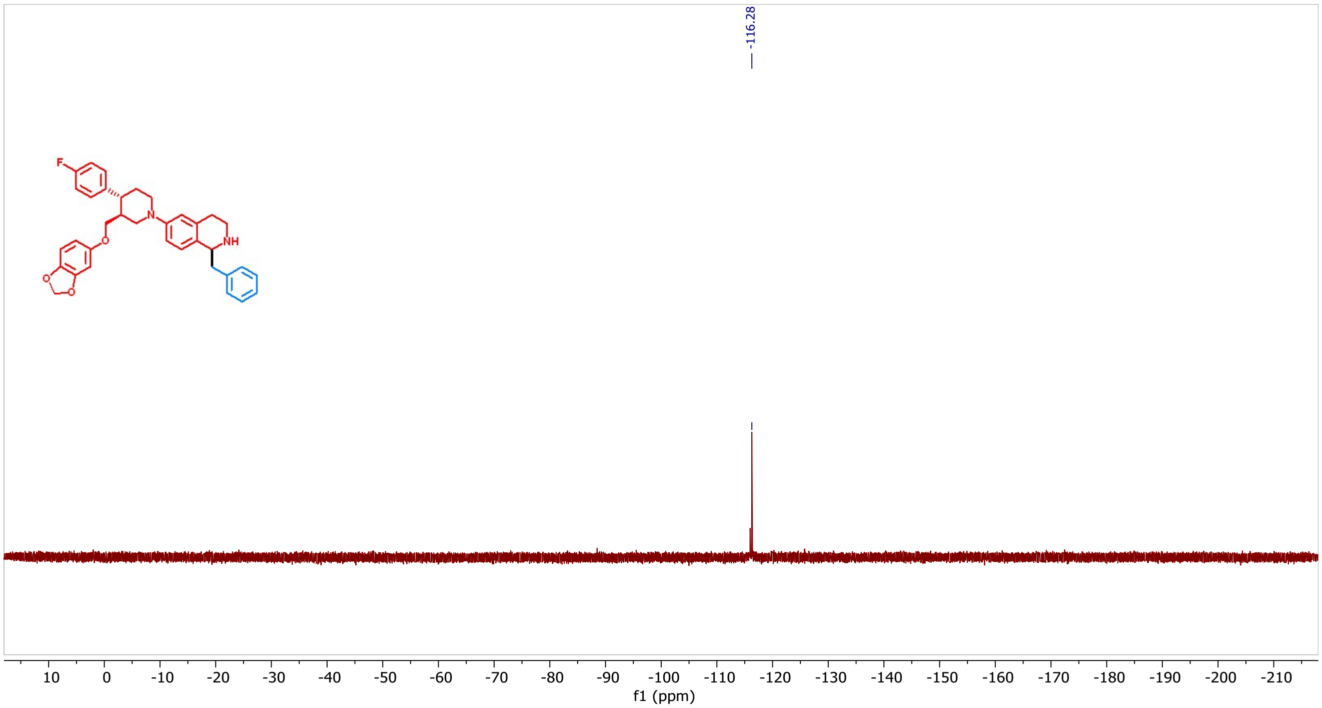

^{1}H NMR (500 MHz, $CDCl_3$) spectrum of **3bn**

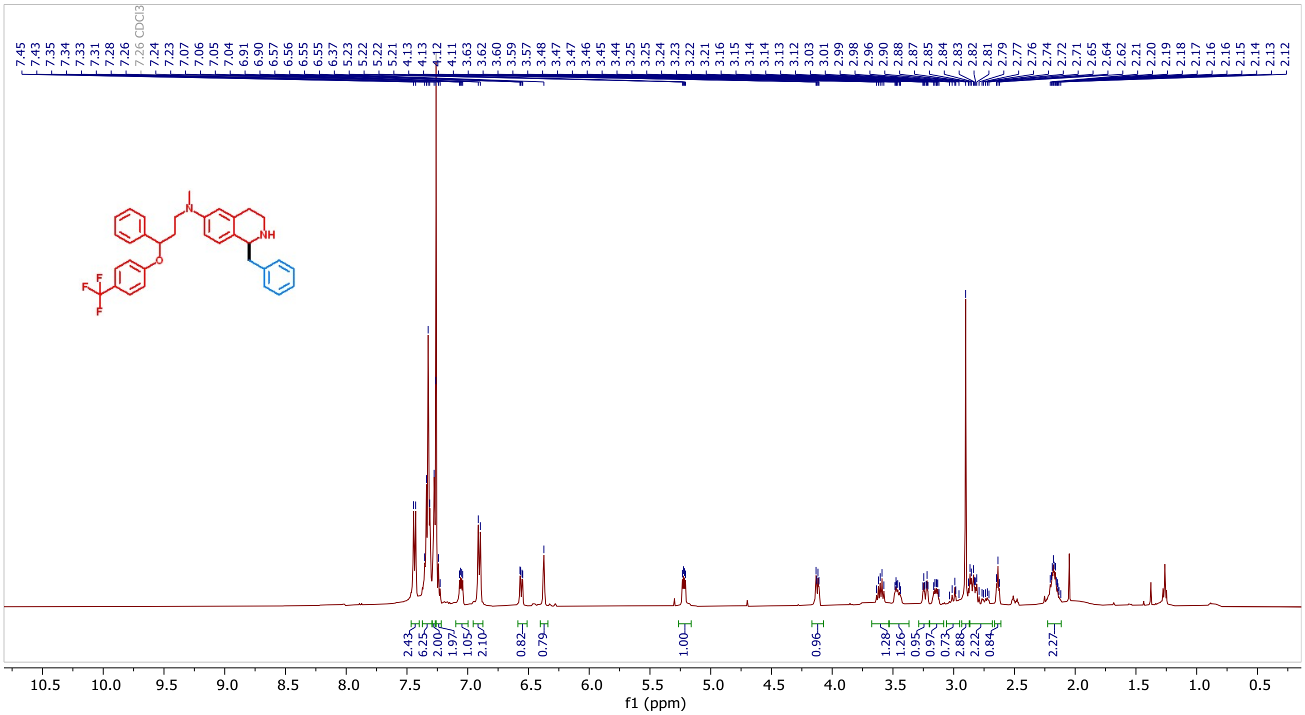

^{1}H NMR (500 MHz, $CDCl_3$) spectrum of **3bn**

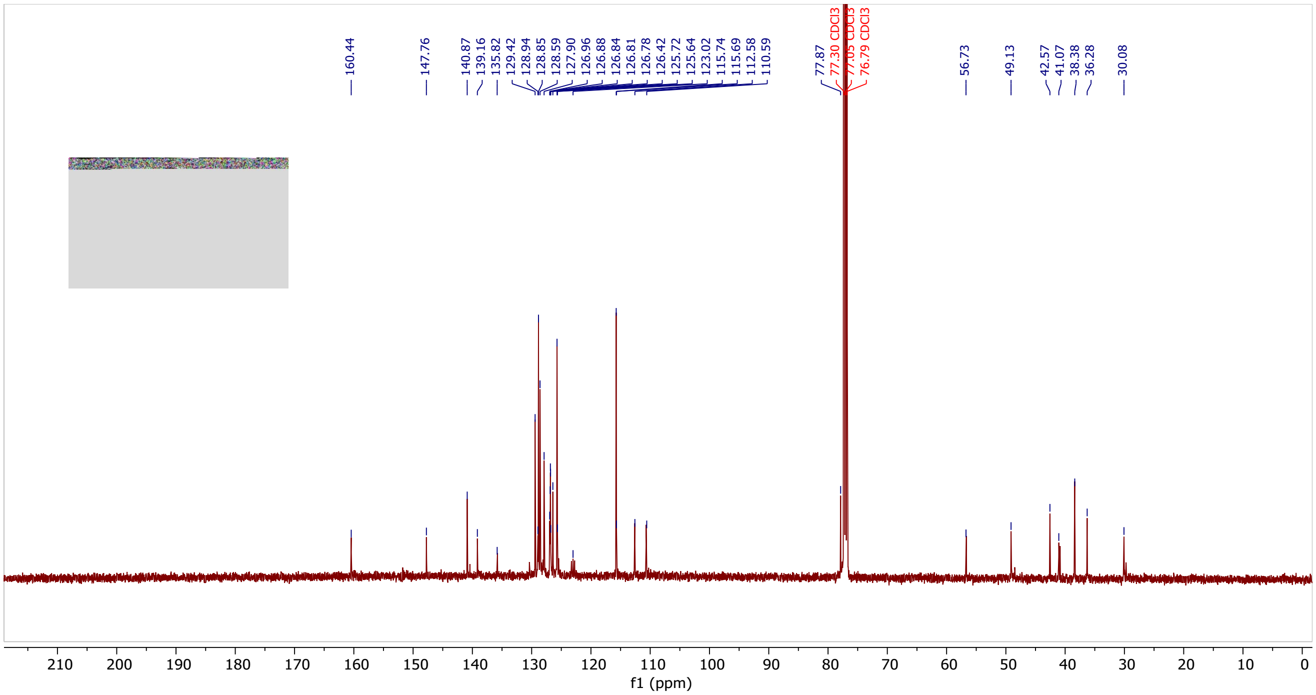

^{19}F NMR (471 MHz, $CDCl_3$) spectrum of **3bn**

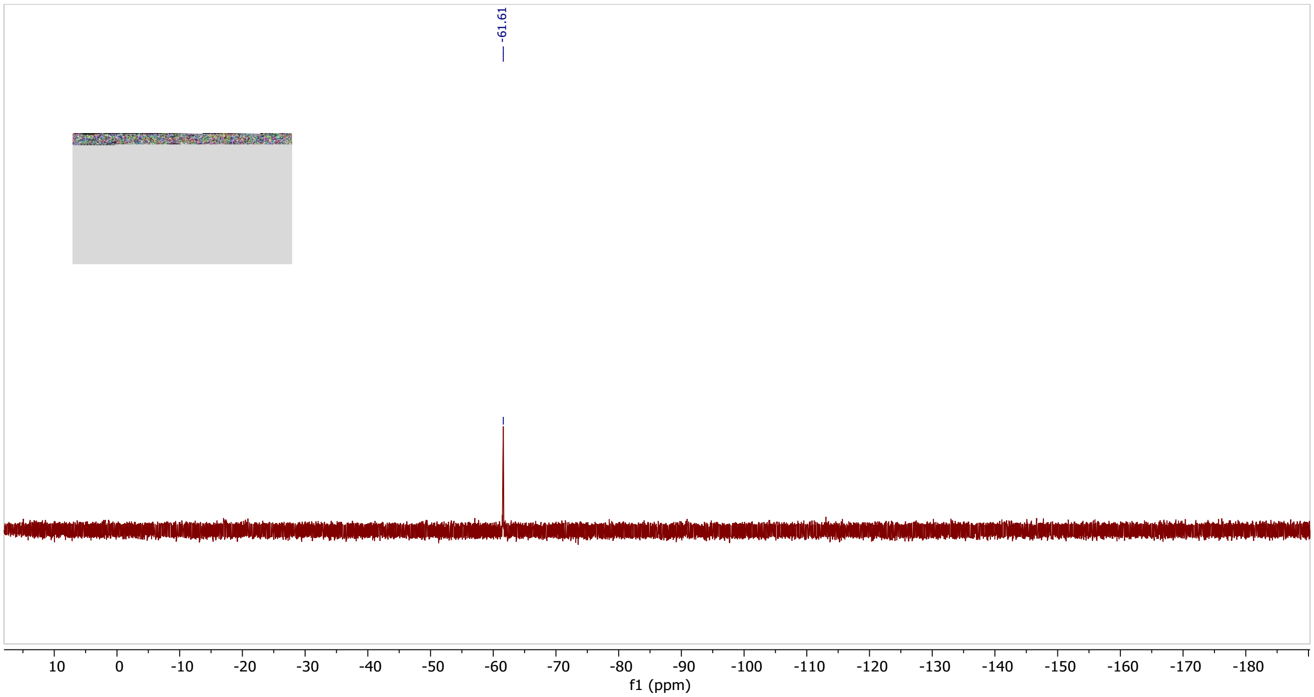

^{1}H NMR (500 MHz, $CDCl_3$) spectrum of **norlaudanosine (3bo)**

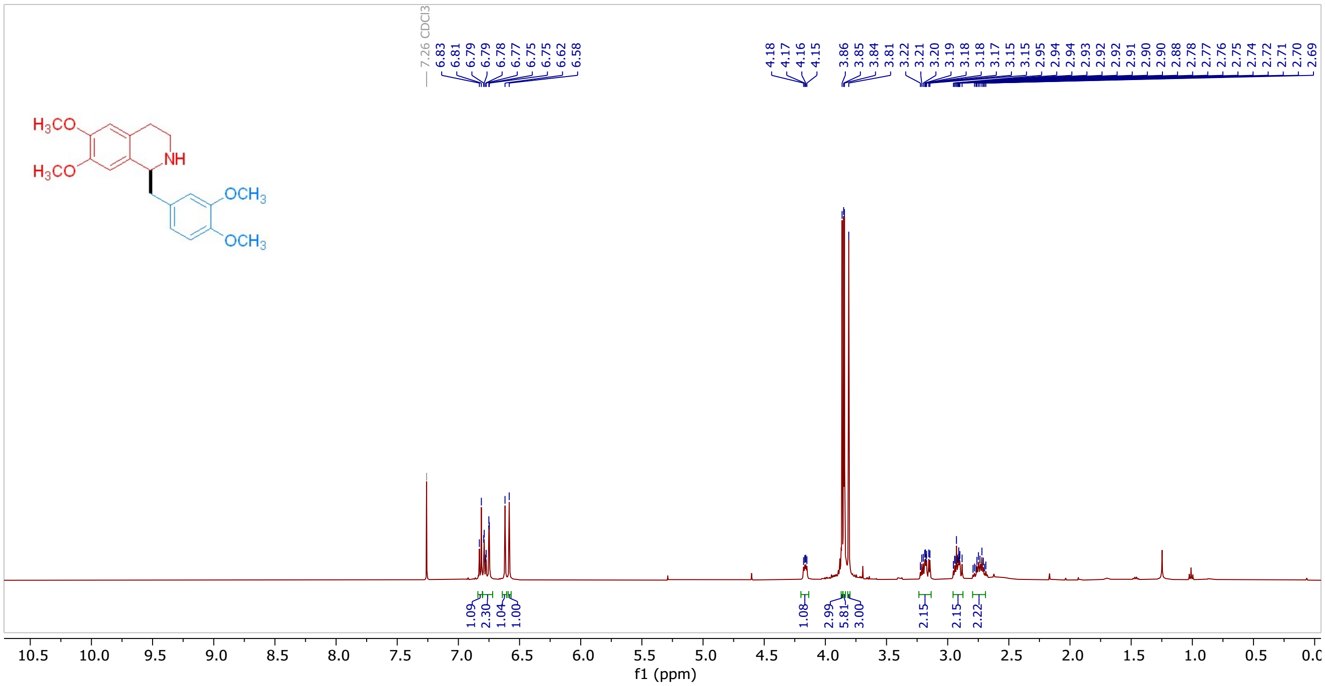

^{13}C{^{1}H} NMR (126 MHz, $CDCl_3$) spectrum of **norlaudanosine (3bo)**

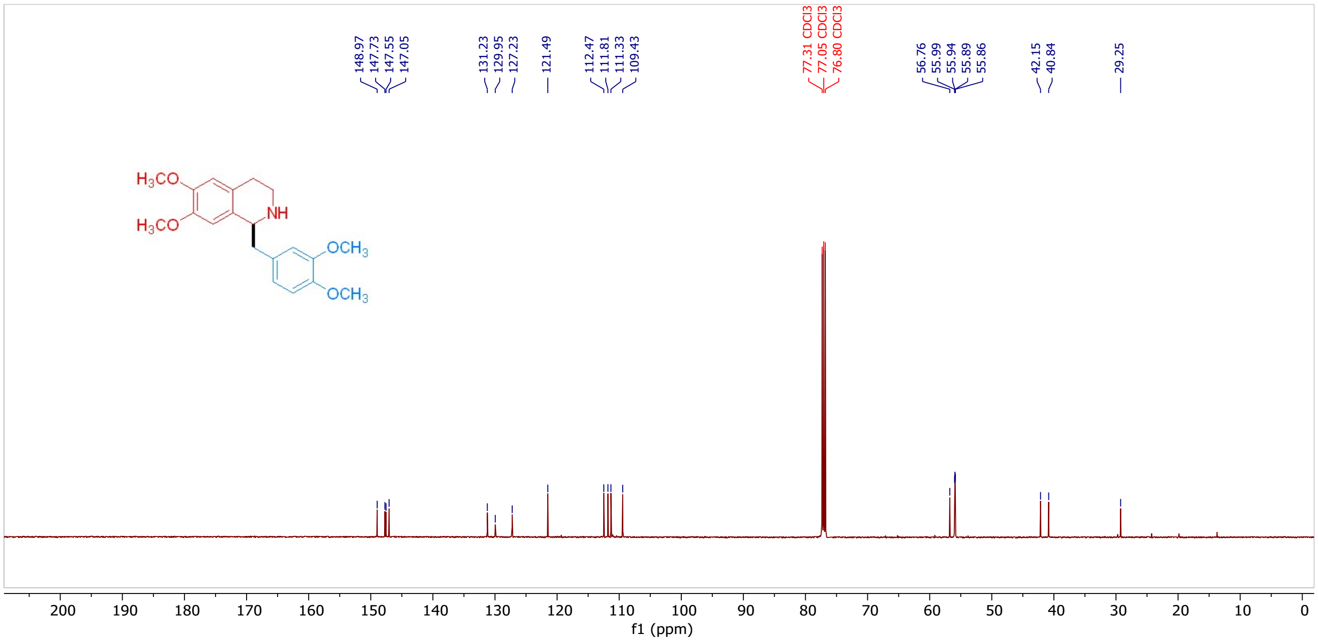

^{1}H NMR (500 MHz, $CDCl_3$) spectrum of **laudanosine (3bp)**

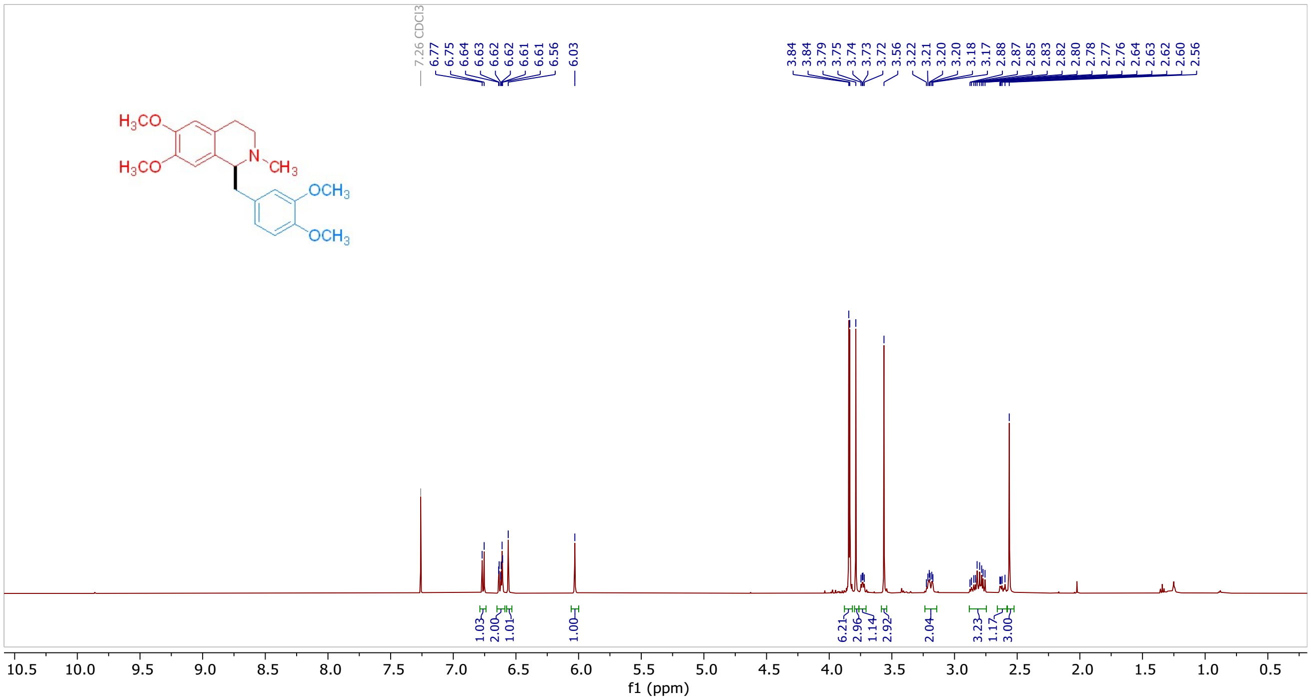

$^{13}C\{^{1}H\}$ NMR (126 MHz, $CDCl_3$) spectrum of **laudanosine (3bp)**

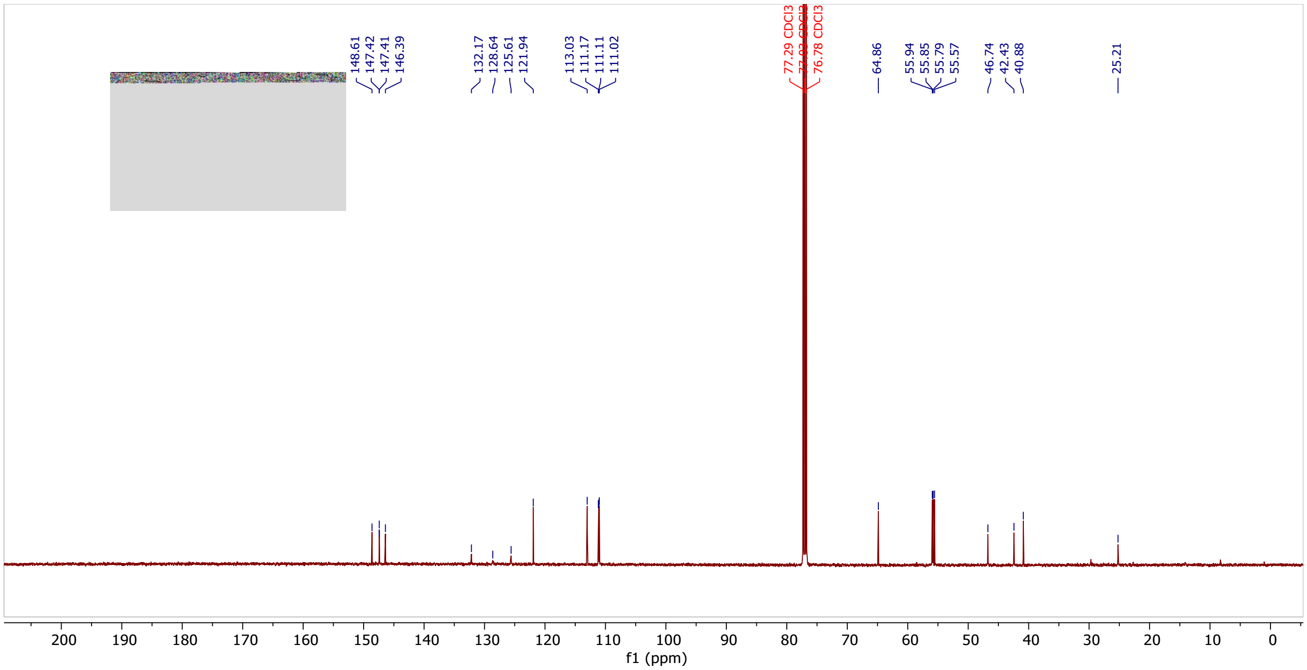

^1H NMR (500 MHz, $CDCl_3$) spectrum of **xylopinine (3bq)**

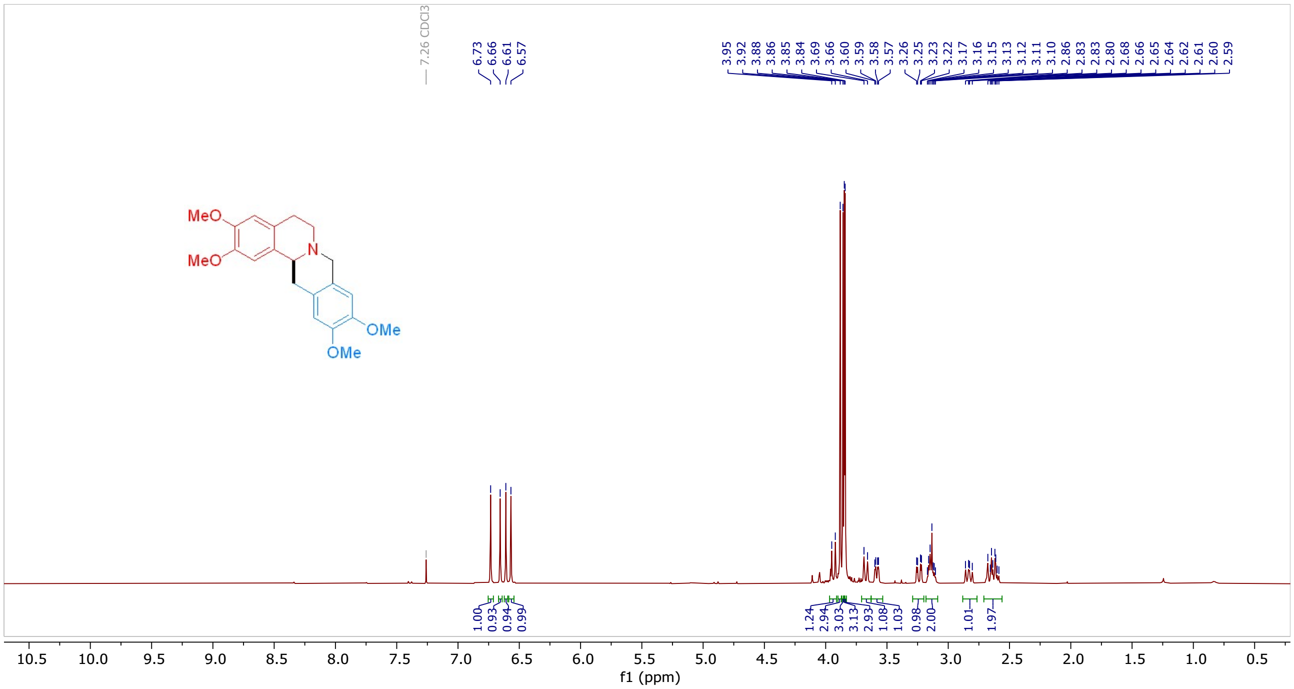

$^{13}C\{^1H\}$ NMR (126 MHz, $CDCl_3$) spectrum of **xylopinine (3bq)**

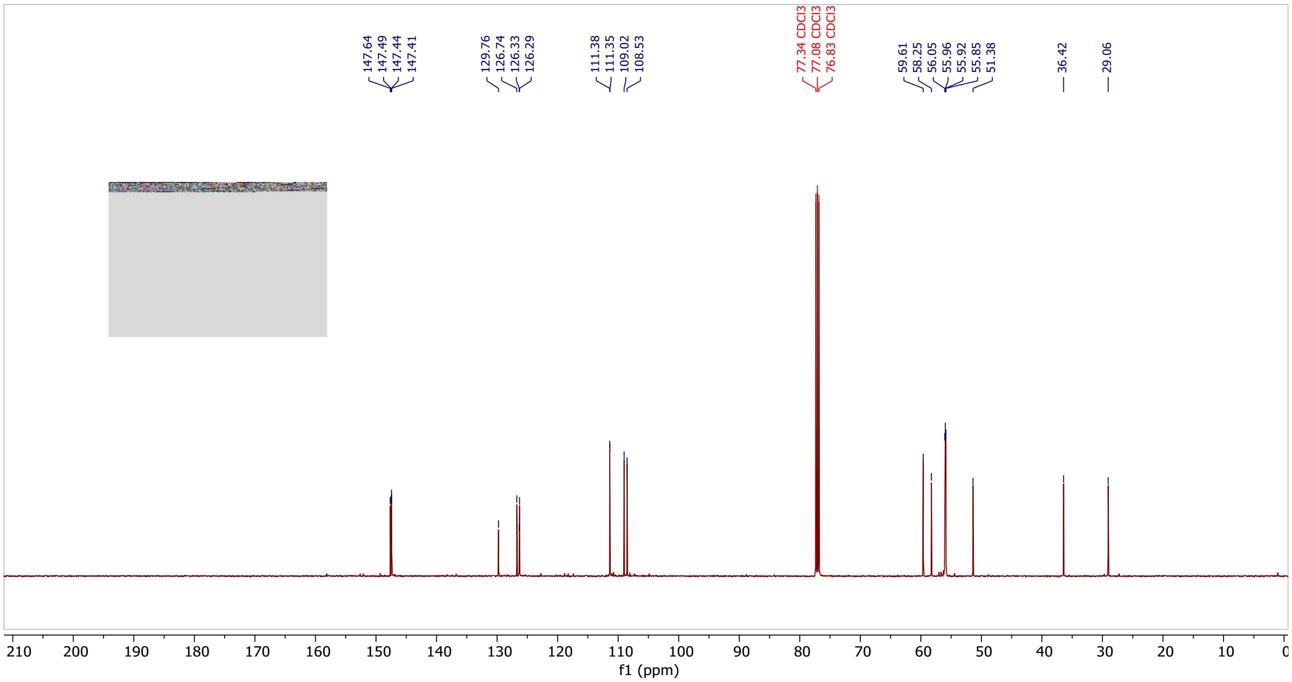

Supplement: Supplementary file 1 [file SC-OLF-D5SC08310A-s001.pdf]
